# The Synthesis of Triazolium Salts as Antifungal Agents: A Biological and In Silico Evaluation

**DOI:** 10.3390/antibiotics11050588

**Published:** 2022-04-27

**Authors:** Serghei Pogrebnoi, Oleg Radul, Eugenia Stingaci, Lucian Lupascu, Vladimir Valica, Livia Uncu, Anastasia Smetanscaia, Anthi Petrou, Ana Ćirić, Jasmina Glamočlija, Marina Soković, Athina Geronikaki, Fliur Z. Macaev

**Affiliations:** 1 Laboratory of Organic Synthesis, Institute of Chemistry, 3 Str. Academiei, MD-2028 Chisinau, Moldova; richserg@gmail.com (S.P.); OlegRadul2022@gmail.com (O.R.); stingacieugenia@gmail.com (E.S.); lucian.lupascu@ichem.md (L.L.); 2 Scientific Center for Drug Research, “Nicolae Testemitanu” State University of Medicine and Pharmacy, MD-2004 Chisinau, Moldova; vvalica@usmf.md (V.V.); livia.uncu@usmf.md (L.U.); anastasia.smetanscaia@gmail.com (A.S.); 3 Department of Pharmacy School of Health, Aristotle University of Thessaloniki, 54124 Thessaloniki, Greece; anthi.petrou.thessaloniki1@gmail.com; 4 Mycological Laboratory, Department of Plant Physiology, Institute for Biological Research, Siniša Stanković, University of Belgrade, Bulevar Despota Stefana 142, 11000 Belgrade, Serbia; rancic@ibiss.bg.ac.rs (A.Ć.); jasna@ibiss.bg.ac.rs (J.G.); mris@ibiss.bg.ac.rs (M.S.)

**Keywords:** triazolium salts, antifungal, microdilution method, docking, CYP51

## Abstract

The control of fungal pathogens is increasingly difficult due to the limited number of effective drugs available for antifungal therapy. In addition, both humans and fungi are eukaryotic organisms; antifungal drugs may have significant toxicity due to the inhibition of related human targets. Furthermore, another problem is increased incidents of fungal resistance to azoles, such as fluconazole, ketoconazole, voriconazole, etc. Thus, the interest in developing new azoles with an extended spectrum of activity still attracts the interest of the scientific community. Herein, we report the synthesis of a series of triazolium salts, an evaluation of their antifungal activity, and docking studies. Ketoconazole and bifonazole were used as reference drugs. All compounds showed good antifungal activity with MIC/MFC in the range of 0.0003 to 0.2/0.0006–0.4 mg/mL. Compound **19** exhibited the best activity among all tested with MIC/MFC in the range of 0.009 to 0.037 mg/mL and 0.0125–0.05 mg/mL, respectively. All compounds appeared to be more potent than both reference drugs. The docking studies are in accordance with experimental results.

## 1. Introduction

In recent years, the frequency and severity of life-threatening fungal infections have increased, mainly in patients with impaired or compromised immunity [1], suggesting the urgent need for the development of new antifungal agents with different modes of action [2].

The majority of mycotic infections are caused by Candida and Aspergillus species. The main cases of nosocomial fungal infections are caused by *Candida albicans*, while *Aspergillus fumigatus* is most of the widespread airborne fungal pathogens [3].

The increased incidence of drug-resistant species of both yeasts and molds, which are causative pathogens of invasive, life-threatening infections, emphasizes the urgent need to develop more effective and safe innovative antifungal drugs [4]. Latest studies revealed that typical azole inhibitors fit the active site of CYP51, a member of the cytochrome P450 (CYP 450) family, through the hydrogen bonds formation, stacking, hydrophobic interactions, and heme coordination [5]. Many effective drugs presently used in clinical belong to a class of azoles (imidazole and triazoles). Nevertheless, the interest in developing new azoles and not only with an extended spectrum of activity still attracts the interest of scientists.

On the other hand, there is a significant amount of information regarding improvements made by researchers in fighting bacteria and fungi drug resistance. Thus, there is a growing interest in phage therapy [6,7]. Photodynamic therapy was used to fight against ESCAPE pathogens [8]. Combarros-Fuertes [9] reported that the combined use of honey and antibiotics reduces the concentrations of drugs necessary to achieve efficacy, thus limiting the likelihood of developing resistance and reverting the susceptibility of some resistant bacteria to antibiotics. There are some reviews regarding the antibiotics from sea microorganisms [10,11,12,13]. Casertano et al. [14], in their extensive review, reported the role of the ascidian′s secondary metabolites as an extraordinary source of novel drug lead structures, describing 160 molecules, including sulfur-containing compounds, alkaloids, meroterpenes, peptides, furanones, and other aromatic derivatives with antimicrobial activity. The activity of some of the metabolites appears to be superior compared to reference drugs.

Zalacain et al. [15] reported a novel specific metallo-β-lactamase inhibitor ANT2681, that restores the activity of meropenem to clinically effective levels against Enterobacterales. They showed that the addition of ANT2681 at 8 μg/mL decreased the MIC50/MIC90 from >32/>32 μg/mL to 0.25/8 μg/mL.

Yang et al. [16] reported that based on the virtual screening approach, they found that carnosic acid exhibited an inhibitory effect on NDM-1. According to the MIC and time-killing assays, carnosic acid can restore the antibacterial activity of meropenem and inhibit NDM-1. Thus, the authors speculated that carnosic acid could improve the antibacterial potency of meropenem by inhibiting NDM-1.

It was recently reported a new class of enzyme blockers, called indole carboxylates (InCs), which inhibit the activity of metallo-β-lactamase (MBL) resistance enzymes produced by bacteria. The results of Schofield et al. [17] revealed that InCs have a substantial potential for clinical development with β-lactam antibiotics. They are actively forwarding InCs toward clinical trials in humans, focusing on low-to-middle income countries in which NDM-mediated (New Delhi Metallo-β-lactamase) resistance is widespread.

Five member heterocycles, with two of the three heteroatoms, are important units in approved drugs. Triazole derivatives are an attractive class of compounds with s wide spectrum of biological activities such as antibacterial [6,7,8], antifungal [7,9,10,11], antitubercular [12,13], anti-inflammatory [14,18], analgesic [19], antiviral [20,21], anti–HIV [22,23], antidiabetic [24,25,26], anticancer [27,28,29], anticonvulsive [26,30,31], carbonic anhydrase inhibitory activity [32,33], antileishmanial [34,35,36] antimalarial [26,37,38]. and others [39,40,41,42].

**Figure 1:**
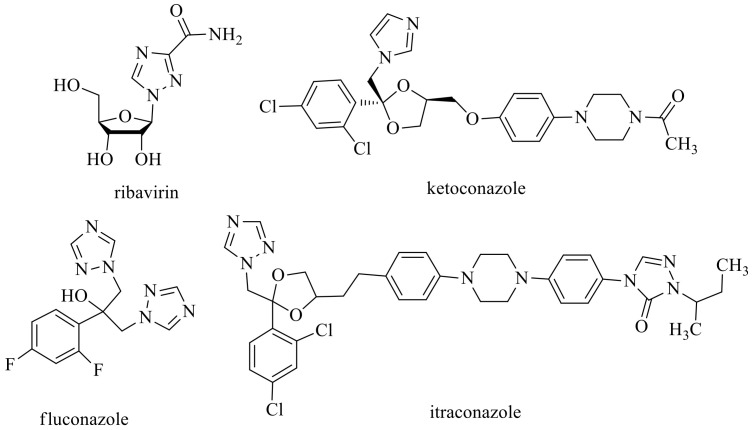
Structure of drugs with the azole moiety.

Furthermore, a triazole scaffold is present in the structure of many approved drugs, such as ribavirin, an antiviral drug for the treatment of Respiratory Syncytial Virus (RSV) infection, hepatitis C and some viral hemorrhagic fevers. In contrast, ketoconazole, fluconazole, and itraconazole are known antifungal drugs. Other drugs that include a triazole moiety are rizatriptan (for the treatment of migraine and headache), alprazolam (anxiolytic), and estazolam (tranquilizer) [39] (Figure 1). Following our research on the development of new antimicrobial agents [43,44], herein, we report the synthesis and evaluation of antifungal activity as well as docking studies of a series of triazolium salts.

## 2. Results and Discussion

### 2.1. Chemistry

**Scheme 1:**
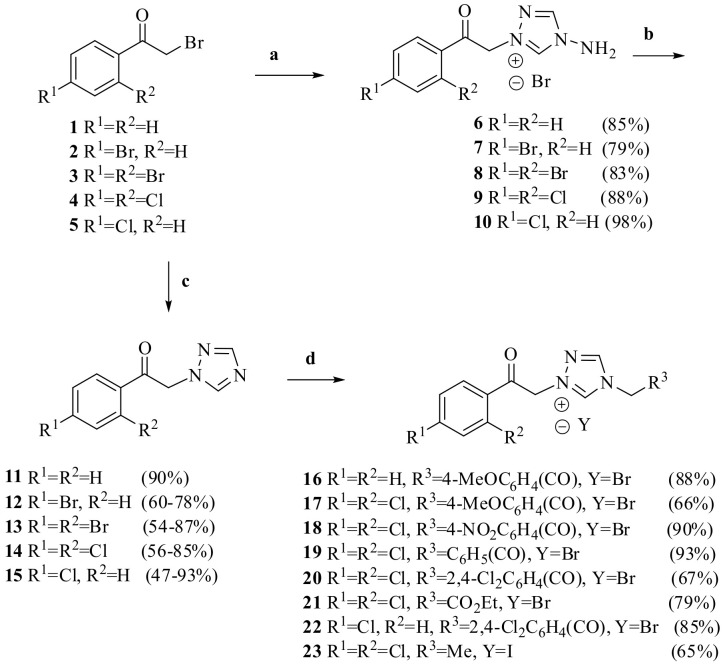
Synthesis of triazolium salts. Reagents and conditions: (**a**) 4-amino-4*H*-1,2,4-triazole, MeCN, rt, 1 h; (**b**) (1) HCl aq., NaNO_2_, 5 °C at 1 h then 3 h at rt; (2) NH4OH to pH = 8–9; (**c**) 1*H*-1,2,4-triazole, Et_3_N, MeCN, 7 h, rt; (**d**) 1-aryl-2-bromoethanones, ethyl 2-bromoacetate or iodoethane, MeCN, 7 h, reflux.

The three types of ammonium salts were synthesized by reacting 1,2,4-triazoles with a stoichiometric amount of the alkyl-, benzyl-, aryl-ethanone halides. Their chemical structures are illustrated in Scheme 1.

The triazolylmethylketones **11-15** were synthesized by two methods. Method A: involves N1-alkylation of 1*H*-1,2,4-triazole by 1-aryl-2-bromoethanones **1-5** in the presence of a base. Method B is a reaction of 4-amino-1*H*-1,2,4-triazole and corresponding 1-aryl-2-bromoethanones in acetonitrile following the removal of an amino group. According to method A, the reaction proceeds in one step, while method B consists of two, but the yield of the desired product is almost twice as high. For example, the yield of 1-(4-chlorophenyl)-2-(1*H*-1,2,4-triazol-1-yl) ethanone **15** by method A is 47%, whereas by method B is 81%. Therefore, we chose method B for the synthesis of triazolylmethylketones **11-15** as the most practical. The obtained triazolylmethylketones **11-15** were quaternized with 1-aryl-2-bromoethanones, ethyl 2-bromoacetate, or ethyl iodide in acetonitrile at reflux. The final products **16-23** yield from 67% to 93%. Based on our experiences in preparing the quaternary ammonium salts **16-23**, it was expected that 1-benzyl-1*H*-1,2,4-triazole also afford the triazolium salts **24-26**.

**Scheme 2:**
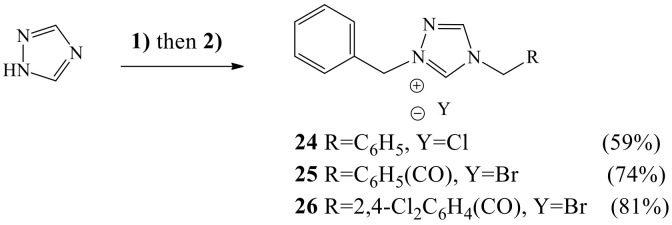
Synthesis of 1-Benzyl-substituted triazolium salts.Reagents and conditions: (**1**) benzyl chloride, K_2_CO_3_, acetone, rt, 24 h; (**2**) benzyl chloride, phenacyl bromide or 2,4-dichlorophenacyl bromide, acetone, reflux 8 h.

Therefore, a reaction sequence 1*H*-1,2,4-triazole→potassium 1,2,4-triazol-1-ide →1-benzyl-1*H*-1,2,4-triazole →1-benzyl-substituted triazolium salts **24-26** was realized in which a benzyl fragment was introduced first at position 1 of 1*H*-1,2,4-triazole followed by the addition of benzyl-, phenacyl- and 2,4-dichlorophenacyl fragments of target substances (Scheme 2).

The structure of the obtained compounds was supported by IR, ^1^H, and ^13^C NMR spectroscopic data and by elemental analysis. The IR spectrum of salts showed absorption bands in the range of 1652–1726 cm^−1^ for compounds **6-23, 25,** and **26**, which is characteristic of the stretching vibration of the carbonyl group. The presence of absorption bands in the range of 1504–1595 cm^−1^ for the compounds **6-26** cm^−1^ suggests the presence of a C=N bond. The examination of the ^13^C NMR spectra of the discussed compounds further confirmed the formation of 1*H*-1,2,4-triazole functionalized salts. The peaks in the ^13^C NMR spectra at 189.0–191.0 ppm are typical of the carbonyl nucleus (Supplementary Materials).

In the ^1^H-NMR spectra, the protons of the triazole ring appeared as broad singlets at 9.33–9.43 ppm and 10.17–10.61 ppm, while the methylene group’s proton is environmental dependent, and their chemical shifts varied between 3.90 to 6.42 for most of the compounds. Other items analysed in the NMR spectra examined the partially decoupled spectra and appear in the experimental section. The purity of the triazolium salts was confirmed by HPLC with acetonitrile/water = 70:30, methanol/water = 70:30, and methanol as the mobile phase at a flow rate of 1.0 mL/min over the range of 2.5 mg/L, 50 mg/L, and 100 mg/L. The HPLC analyses were carried out at room temperature under isocratic conditions. The purity of salts with 1-phenylethanone moiety at the 1-arylethanone of triazole ring **6, 16, 25** was ≥ 98.70%. The HPLC chromatograms of the triazolium salts **6-10** and **16-26** appear as supporting information.

### 2.2. Biological Evaluation

All synthesized compounds were tested for their antifungal activity by the microdilution method against the panel of eight fungi. All compounds showed good antifungal activity with a MIC/MFC in the range of 0.0003–0.2/0.0006–0.4 mg/mL (0.0005–0.055/0.001–1, 116 mmol/mL) (Table 1). The order of antifungal activity can be presented as: **19 > 8 > 17 > 22 > 20 > 7 > 9 > 21 > 18 > 16 > 23 > 24 > 6 > 25**.

Compound **19** exhibited the best activity among all tested with MIC and MFC in the range of 0.009–0.037 mg/mL and 0.0125–0.05 mg/mL, respectively, while the lowest activity was displayed by compound **25** (MC/MFC in arrange of 0.025–0.20/0.05–0.40 mg/mL). Ketoconazole showed antifungal potential with MIC/MFC at 0.28–1.88/0.38–2.82 mg/mL, while MIC and MFC of bifonazole were in the range of 0.32–0.64/0.64–0.81 mg/mL. Thus, all compounds appeared to be more active than ketoconazole and bifonazole. Generally, compound **15** displayed up to ten times stronger antifungal potential than Ketoconazole and two to twelve times higher than Bifonazole. Compound 19 was a more powerful antifungal agent than the reference drugs. It possessed even up to 50 to 209 times stronger inhibitory and fungicidal potential against all tested micromycetes. Generally, all tested compounds displayed significantly higher antifungal activities than the reference compounds Ketoconazole and Bifonazole.

In general, fungi showed different sensitivity towards compounds tested. Thus, the sensitivity of compounds against the most resistant fungus, *A. fumigatus* can be presented as: **7 = 8 > 19 = 17 > 9 = 18 = 21 = 22 > 20 = 16 > 6 = 24 = 23 = 25**, while towards the most sensitive fungal specie *T. viride* as: **20 > 8 = 19 = 22 = 17 > 21 > 9 = 18 > 24 > 6 = 16 = 25 > 23 > 7**. Those compounds with the lowest activity were **24**, **23,** and **25** against *A. fumigatus*, which showed two-to-five times better antifungal potential compared to both Ketoconazole and Bifonazol. Differences in sensitivity were observed not only among different species, but also for each fungus. At the same time, almost all *Aspergillus* species except of *A. versicolor* were found to be sensitive towards compound **7**, while Penicillium species mostly towards compound **8**.

Compounds **8, 19, 22,** and **17** (MIC /MFC at 0.009/0.0125 mg/mL) as well as compound **20** (MIC/MFC 0.0006/0.00125 mg/mL) displayed excellent activity against *T. viride*, while compound **20** showed the best activity among all tested compounds also against *A. versicolor* with MIC and MFC at 0.0003 mg/mL and 0.0006 mg/mL, respectively, followed by compound **16** (MIC/MFC at 0.0006/0.0125 mg/mL. Good activity against *A. versicolor* was also shown by compounds **8** and **19** with MIC /MFC at 0.0125/0.025 mg/mL. It should be mentioned that the same good activity was observed for compound **8** against *P. funiculosum* and *P. ochraceus*.

The study of structure–activity relationships revealed the presence of (2,4-dichlorophenyl)ethanone at N-1 of a triazole moiety as a substituent as well as 1-phenylethanone at N-4 of triazole **19** is beneficial for antifungal activity. Replacement of (2,4-dichlorophenyl)ethanone by (2,4-dibromophenyl)ethanone and acetophenone by amino group led to a less potent compound **8**.

The replacement of 1-phenylethanone in compound **19** by 1-(4-methoxyphenyl)ethanone decreased more than activity **17**. Finally, the presence of the benzyl group as a substituent at N-1 of a triazole ring and 1-phenylethanone at N-4 had a very negative effect on the antifungal activity, leading to the less active compound **25**. Among the compounds with a 2,4-dichlorobenzene moiety at the 1-arylethanone of the triazole ring, the activity order can be presented as follows: **19 > 17 > 20 > 9 > 21 > 18 > 23**. In general, it seems that these groups are the most active. Thus, the presence of 1-phenylethanone as a substituent at N-4 of triazole ring, as already mentioned, is favorable for the activity; the opposite was observed with 1-(4-nitrophenyl)ethanone **18** and ethyl group as substituents **23**. On the other hand, the dibromo-substituted compound **8** is more favorable than bromo-substituted compound **7.** In the case of an unsubstituted aromatic moiety at N-1 of triazole ring, the activity order is **16 > 24 > 6 > 25.** This fact indicates that the presence of 1-(2,4-dichlorophenyl)ethanone at N-4 of triazole has a positive influence on the antifungal activity, whereas the presence of 1-phenylethanone in compound **25** has a negative one. Thus, the activity of compounds depends not only on their nature but also on their position on the triazole ring.

### 2.3. Docking to Antifungal Targets

In order to predict the possible mechanism of antifungal activity of the compounds, all the synthesized compounds and the reference drug ketoconazole were docked to lanosterol 14α-demethylase of *C. albicans* and DNA topoisomerase IV (Table 2).

**Figure 2:**
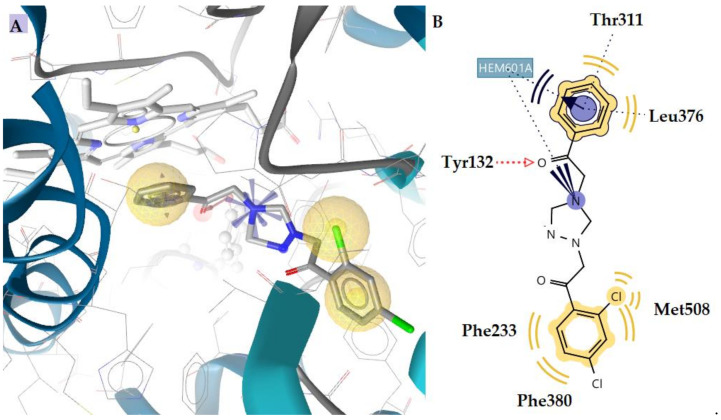
Docking pose of the most active compound **19** (**A**) and 2D diagram (**B**) in lanosterol 14α-demethylase of *C*. *albicans* (CYP51_ca_). Red-dotted arrows indicate H-bond, blue arrows positive ionizable, blue spheres indicate the aromatic, and yellow spheres indicate the hydrophobic interactions.

**Figure 3:**
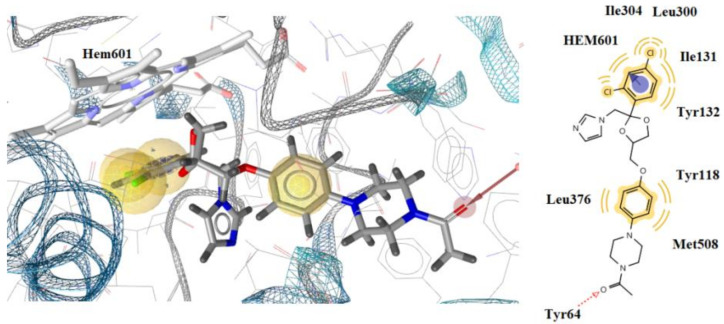
Docked conformation of reference drug ketoconazole in lanosterol 14α-demethylase of *C*. *albicans* (CYP51_ca_). Red-dotted arrows indicate H-bond, blue spheres indicate the aromatic, and yellow spheres indicate the hydrophobic interactions.

**Figure 4:**
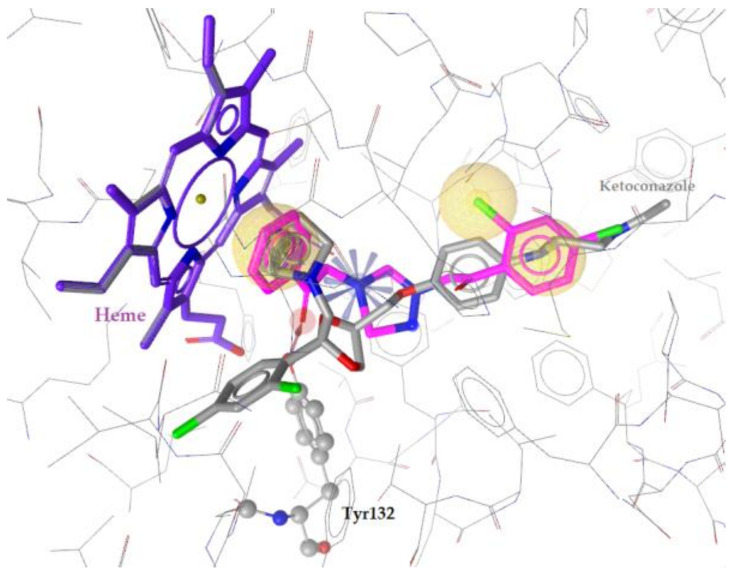
The superposition of the most active compound **19** (magenta) and Ketoconazole (grey) in lanosterol 14α-demethylase of *C*. *albicans* (CYP51_ca_). The heme group is represented in purple color.

The docking results revealed that the most active compound **19** takes place inside the enzyme interacting with the heme group of the enzyme throughout its benzene ring, forming aromatic interactions. Additional positive ionizable interactions were detected between the N atom of the thiazole ring of the compound and the Fe atom of the heme group. Moreover, hydrophobic interactions between Phe233, Thr311, Leu376, Phe380, and Met508 and the benzene rings of the compound were detected (Figure 2). Ketoconazole also interacts, thus forming interactions with its hydrophobic benzene ring and aromatic interactions (Figure 3). Indeed, the superposition of compound **19** and ketoconazole showed that they bind to the enzyme in the same manner (Figure 4). This is probably the reason why compound **19** has a high inhibition profile.

**Figure 5:**
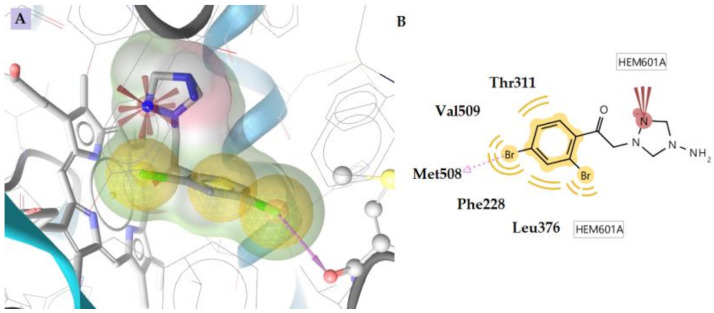
Docking pose of the compound **8** (**A**) and 2D diagram (**B**) in lanosterol 14α-demethylase of *C*. *albicans* (CYP51_ca_). Pink-dotted arrows indicate halogen-bond interaction; the red arrows indicate negatively ionizable.

Regarding the second-most active compound 8 (Figure 5), docking studies showed that it is placed inside the enzyme by the side of heme group, binding to the Fe (II) ion throughout its N atom of triazole ring. It is believed that this interaction increases its binding affinity and justifies its high antifungal activity. Furthermore, compound **2** forms one halogen bond between the Br substituent of the benzene ring and the residue of Met508. Hydrophobic interactions were also detected between residues Phe228, Thr311, Leu376, Met508, Val509, and the benzene ring of the compound **8** (Figure 5B). 

Molecular docking studies also were performed on 14-alpha demethylase (CYP51B) from the fungus *Aspergillus fumigatus* in a complex with voriconazole, because this yeast was used in the biological evaluation in order to have a clear picture of the binding mode of the compounds.

Results of docking studies are presented in Table 3 and revealed that estimated free energies of binding of the compounds were in accordance with this experimental data for this yeast. 

**Figure 6:**
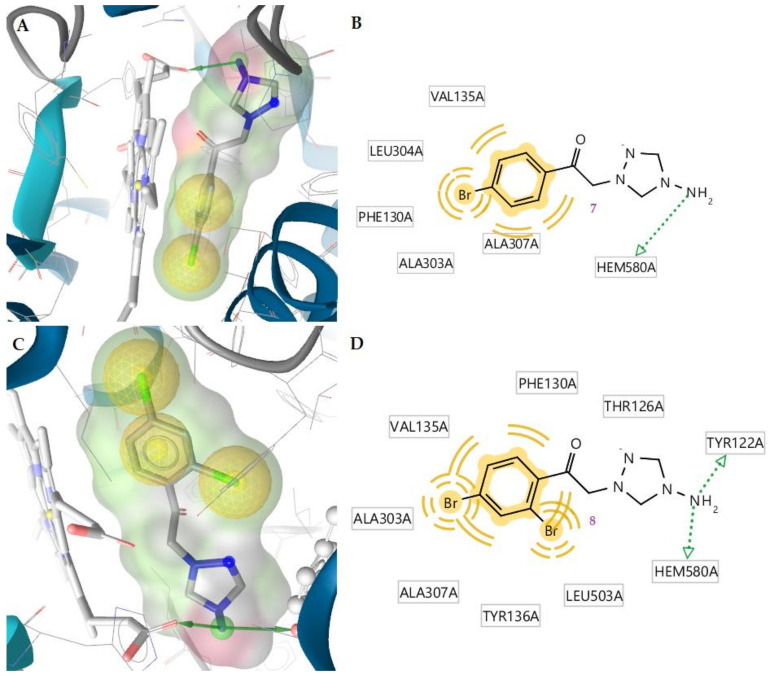
Docking pose of the compound **7** (**A**) and 2D diagram (**B**) in 14-alpha demethylase (CYP51B) from the fungus *Aspergillus fumigatus*. Docking pose of the compound **8** (**C**) and 2D diagram (**D**) in 14-alpha demethylase (CYP51B) from *Aspergillus fumigatus*. Green dotted arrows indicate hydrogen bonds, and yellow spheres indicate the hydrophobic interactions.

The most active compounds **7** and **8** exhibited low estimated free energies of binding, which are reflected in their binding mode. In particular, compound 7 interacted with the heme group of enzymes, forming a hydrogen bond with its amino group substituent and several hydrophobic interactions with residues Phe130, Val135, Ala307, Ala303, and Leu304. All of these interactions contributed to the stabilization of the complex enzyme compound. Compound 8 also formed a hydrogen bond with the heme group of the enzyme through its amino substituent and another with residue Tyr122. Moreover, hydrophobic interactions with residues Ala307, Ala303, and Leu503 were detected (Figure 6).

It is important to note that the formation of a strong hydrogen bond with the heme group reflects the high inhibition profile of compounds 7 and 8 and likely explains it. Compounds **17** and **19,** with high antifungal activity, also formed this interaction (Table 3).

**Figure 7:**
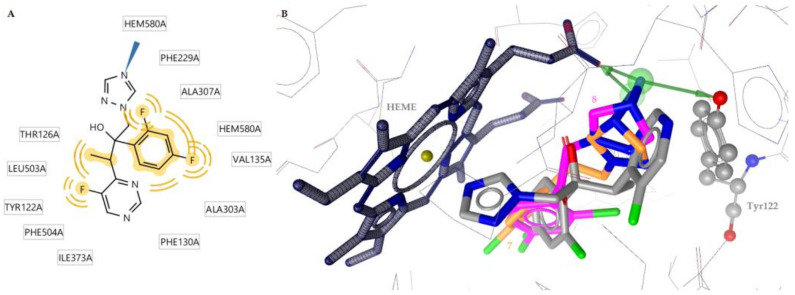
(**A**) 2D diagram of the syncrystalized drug voriconazole in 14-alpha demethylase (CYP51B) from the fungus *Aspergillus fumigatus*. (**B**) Superposition of most active compounds **8** (magenta), **7** (orange), and voriconazole (grey) 14-alpha demethylase (CYP51B) from the fungus *Aspergillus fumigatus*. Green arrows indicate a hydrogen bond; yellow spheres indicate the hydrophobic interactions, and the blue line indicates Fe-binding.

The superposition of most active compounds **7** (magenta), **7** (orange) with voriconazole (grey) in 14-alpha demethylase (CYP51B) from *Aspergillus fumigatus* (Figure 7), revealed that they took place inside the enzyme along with the heme group and were bound to the enzyme in the same manner.

### 2.4. Drug-likeness

The number of violations of various rules, viz. Lipinski, Ghose, Veber, Egan, and Muegge, along with the bioavailability and drug-likeness scores, are given in Table 4. The results revealed that none of the compounds violated any rule, and their bioavailability score was approximately 0.55. All compounds exhibited moderate to good drug-likeness scores ranging from −1.15 to 0.17. Compound **26** appeared to be the best in terms of their in silico predictions, with a drug-likeness score of 0.16 without any rule violation.

## 3. Materials and Methods 

### 3.1. Chemistry

The chemicals used were of reagent grade and used as received. The removal of all solvents was carried out under reduced pressure. ^1^H and ^13^C NMR spectra were recorded for 2% solutions on a Bruker-Avance III spectrometer (400.13 and 100.61 MHz) (Karlsruhe, Germany). Chemical shifts δ are given in ppm, referring to the signal center using the deuterated solvent peaks for reference. The IR spectra were recorded on a Spectrum 100 FT-IR spectrophotometer (Perkin–Elmer, Waltham, MA, USA) using the universal ATR sampling accessory. All products were analyzed by CHN elemental analysis (Elementar Vario EL analyzer) (Santa Clara, CA, USA). Melting points (uncorrected) were determined on a Boetius apparatus (Dresden, Germany). The GC-MS analysis was performed using an Agilent Technologies 7890A gas chromatograph coupled with a 5975C Mass Selective Detector (MSD) equipped with a splitless injector (1 µL) (Santa Clara, CA, USA). The analysis was carried out on a fused silica capillary HP-5MS calibrated column (30 m × 0.25 mm i.d.; film thickness 0.25 µm). The injector and detector temperatures were kept at 250 °C. Helium was used as carrier gas at a flow rate of 1.1 mL/min; the oven temperature program was 70 °C/2 min, which was then programmed to 200 °C at the rate of 5 °C/min, and finally to 300 °C at the rate of 20 °C/min; the split ratio was 1:50. The MSD ionization energy of 70 eV, scan time of 1 s, acquisition mass range was from 30 to 450 amu, and a solvent delay of 3 min. 

For LC/MS analyses, a Shimadzu LC/MS 2020 single quadrupole mass spectrometer with an electrospray ion source (ESI) was used. A nitrogen gas generator N2LCMS (Nitrogen Generator, Claind) was employed throughout this study. The temperature of the desolvation line and heat block were set at 250 and 200 °C, respectively. The N_2_ nebulizer gas flow was maintained at 1.5 L min^−1,^ and the drying gas flow was set at 15 L min^−1^, while the interface voltage was set at 4.5 kV in positive or negative mode. The sample injection volume was 5 μL in all cases. The carrier was a mixture of 0.1% aqueous formic acid/methanol, 50/50 *v*/*v.* The flow rate was set at 0.5 mL min^−1^. 

MS scan: 100–600 *m*/*z*. Sample concentration: 500 μg mL^−1^ in MeOH.

The HPLC analyses were performed using high-pressure liquid chromatography with a UV detector on a Shimadzu Prominence system coupled to a Shimadzu UV-SPD-20PD detector (Shimadzu, Kyoto, Japan) over the range of 2.5–100 mg/L (MeOH, MeOH:H_2_O = 70:30 and MeCN:H_2_O= 70:30). The purity of salts with 1-phenylethanone moiety at the 1-arylethanone of triazole ring **6, 16, 25** was ≥98.70%. HPLC chromatograms of the triazolium salts **6-10** and **16-26** appear as supporting information. Thin-layer chromatography was carried out on Merck aluminum TLC plates and silica gel 60 coated with fluorescent indicator F254.

#### 3.1.1. Typical Experimental Procedure for the Synthesis of 1-substituted-2-(1H-1,2,4-triazol-1-yl) ethanones **11-15**

*Method (A).* To a solution of 1*H*-1,2,4-triazole (0.76 g, 11 mmol) and triethylamine (1.39 mL, 11 mmol) in MeCN (20 mL) was added to a solution of 1-aryl-2-bromo-ethanone (2.33 g, 11 mmol) in MeCN (5 mL). The reaction mixture was stirred at 20 °C for 7 h (TLC control). The reaction mixture was poured into water, extracted with chloroform, and dried with anhydrous sodium sulfate.

The usual work-up produced a crude residue, which was submitted to flash chromatography on a silica gel column. Elution with 3% EtOAc in petroleum ether gave pure (GC-MS analysis) target compounds. 

*Method (B).* In a three-necked flask equipped with a mechanical stirrer, cooler, and dropping funnel, a solution of 4-amino-1,2,4-triazole (0.92 g, 11 mmol) in MeCN (30 mL) was added a stirring solution of 1-aryl-2-bromo-ethanone (11 mmol) in MeCN (10 mL). The reaction mixture was boiled for 40 minutes. After cooling, the precipitated crystals were filtered, washed with cold acetonitrile, and dried. A solid substance was obtained. From the mother liquor, after distilling off the solvent, another amount of product was obtained. 

The diazotization of the obtained salt: In a three-necked flask equipped with a stirrer, a thermometer, and a dropping funnel, the substance (10 mmol) obtained in the previous experiment was suspended in water (50 mL). Then, the HCl (1.7 mL of 37% solution) was added and heated until the salt was completely dissolved. The reaction mixture was cooled to 0 °C, and a solution of sodium nitrite (0.83 g, 12 mmol) in water (10 mL) was added slowly with stirring for 1 hour, maintaining the temperature within + 5- 0 °C. By the end of the addition of sodium nitrite, the reaction mixture thickened. Stirring at this temperature continued for another 1 hour, after which the temperature was raised to 20 °C. In this case, gas evolution is observed. The reaction mixture was kept at room temperature for 3 h, treated with NH_4_OH to pH = 8–9, and extracted with chloroform. The extract was washed with water, dried over anhydrous sodium sulfate, and then evaporated under reduced pressure. The resulting product was crystallized to give pure products (GC-MS analysis).

#### 3.1.2. General Experimental Procedure for Synthesis Ammonium Salts **6-10** and **15-23**

A solution of 1-aryl-2-bromo-ethanones, ethyl 2-bromoacetate, or iodoethane (10.5 mmol) in MeCN (10 mL) was added to a solution of 1-substituted-2-(1*H*-1,2,4-triazol-1-yl)ethanone (10 mmol) in MeCN (30 mL). The reaction mixture was refluxed for 7 h. The white precipitate was filtered off, washed with acetonitrile, and dried in a vacuum. Sometimes it depends on the nature of the starting materials to see if a distillation of half of the solvent is necessary.

#### 3.1.3. General Experimental Procedure for the Synthesis of 1-benzyl-4-substituted 1H-1,2,4-triazol-4-ium halides **24-26**

To a solution of 1*H*-1,2,4-triazol (0.1 mmol) in acetone (10 mL) were added benzyl chloride (0.1 mmol) and K_2_CO_3_ (0.1 mmol) in this order. The reaction mixture was stirred at room temperature for 24 h, after which acetone (10 mL) was added. The organic layer after filtration was evaporated under reduced pressure. The residue was dissolved in MeCN (25 mL) followed by the addition of 0.1 mmol the benzyl chloride, the 2-bromo-1-phenylethanone or the 2-bromo-1-(2,4-dichlorophenyl)ethanone. The reaction mixture was stirred at room temperature for 2 days. Solid products were obtained by filtration and drying at room temperature.

4-Amino-1-(2-oxo-2-phenylethyl)-1H-1,2,4-triazol-4-ium bromide (**6**)

Yield 2.4 g, (85%), white crystals (MeCN), mp 185–187 °C. IR (ν/cm^−1^): 3150, 3122,3075, 3033, 2928, 2912, 1687, 1645, 1593, 1579, 1557, 1449, 1414, 1399, 1352, 1313, 1306, 1232, 1200, 1185, 1150, 1090, 1081, 1043, 1008, 953, 924, 870, 854, 825, 687, 677. ^1^H NMR (DMSO-d6, 400 MHz); 10.17 (1H, s), 9.33 (1H, s), 8.08 (2H, d, *J* = 7.5 Hz), 7.79 (1H, t, *J* = 7.4 Hz), 7.65 (2H, t, *J* = 7.7 Hz), 7.17 (2H, s), 6.30 (2H, s). ^13^C NMR (DMSO-d6, 100 MHz): 190.9, 145.6, 144.6, 135.3, 133.9, 129.6, 128.8, 58.9. Anal. Calcd for C_10_H_11_BrN_4_O _C_ 42.42; H 3.92; N 19.79. Br, 28.22%. Found C 42.34; H 3.95; N 19.67. Br, 28.11%. MS: calcd for *m*/*z* 203.22, found: 203

4-Amino-1-(2-(4-bromophenyl)-2-oxoethyl)-1H-1,2,4-triazol-4-ium bromide (**7**)

Yield 2.86 g, (79%,) white crystals (ethanol), mp 223–224 °C. Lit. 223–224 °C [45]. Anal. Calcd for C_10_H_10_Br_2_N_4_O C 33.18; H 2.78; Br 44.14; N 15.48%. Found C 32.00; H 2.59; Br 44.24; N 15.31%. 

4-Amino-1-(2-(2,4-dibromophenyl)-2-oxoethyl)-1H-1,2,4-triazol-4-ium bromide (**8**)

Yield 3.65 g, (83%), white crystals (ethanol), mp 212–213 °C. IR (ν/cm^−1^): 3222, 3138, 3097, 3033, 2992, 2948, 2888, 1718, 1704, 1639, 1575, 1563, 1548, 1470, 1402, 1367, 1353, 1318, 1274, 1217, 1162, 1146, 1073, 1055, 998, 986, 872, 817, 783, 725, 710. ^1^H NMR (DMSO-d6, 400 MHz): 10.32 (1H, s), 9.37 (1H, s), 8.12 (1H, d, *J* = 1.6 Hz), 8.01 (1H, d, *J* = 8.4 Hz), 7.88 (1H, dd, *J* = 8.4.1.6 Hz),7.25 (2H, br.s), 6.24 (2H, s). ^13^C NMR (DMSO-d6, 100 MHz):191.9, 145.7, 144.5, 137.1, 134.6, 132.6, 131.6, 127.4, 121.3, 60.1. Anal. Calcd for C_10_H_9_Br_3_N_4_O C 27.24; H 2.06; Br 54.37; N 12.71%. Found C 27.31; H 1.89; Br 54.30; N 12.55%. MS: calcd for *m*/*z* 361.01, found: 361.

4-Amino-1-(2-(2,4-dichlorophenyl)-2-oxoethyl)-1H-1,2,4-triazol-4-ium bromide (**9**) 

Yield 3.09 g, (88%), white crystals (MeCN), mp 196–198 °C. Lit. 195–196 °C [45] Anal. Calcd for C_10_H_9_BrCl_2_N_4_O. C 34.12; H 2.58; Br 22.70; Cl 20.14; N 15.92%. Found C 34.00; H 2.37; Br 22.69; Cl 20.22; N 16.06%.

4-Amino-1-(2-(4-chlorophenyl)-2-oxoethyl)-1H-1,2,4-triazol-4-ium bromide (**10**)

Yield 3.1 g, (98%), white crystals (ethanol), mp 201–202 °C. Lit. 201–202 °C [45]. Anal. Calcd for C_10_H_10_BrClN_4_O C 37.82; H 3.17; Br 25.16; Cl 11.16; N 17.64. Found C 37.77; H 2.98; Br 25.15; Cl 11.16; N 17.49.

1-Phenyl-2-(1H-1,2,4-triazol-1-yl)ethanone (**11**)

Yield 1.85 g, (90%), white crystals (hexane), mp 107–108 °C. Lit. 108–109 °C. [46] Anal. Calcd for C_10_H_9_N_3_O C 64.16; H 4.85; N 22.45%. Found C 64.01; H 5.03; N 22.62%. MS: calcd for *m*/*z* 187.2, found 186.9, 159, 105, 91, 77, 65, 51, 39, 32.

1-(4-Bromophenyl)-2-(1H-1,2,4-triazol-1-yl)ethanone (**12**)

Method A: Yield 1.58 g, (60%), Method B: Yield 2.05g, (78%), white crystals (MeCN), mp 163–164 °C. Lit. 165–166 °C [45]. Lit. 167–168 °C. [46] Anal. Calcd for C_10_H_8_BrN_3_O C 45.14; H 3.03; Br 30.03; N 15.79%. Found C 44.98; H 2.88; Br 30.12; N 15.66%. %. MS: calcd for *m*/*z* 264.99, found 264.9, 238, 207, 195, 182, 170, 154, 130, 117, 104, 90, 76, 63, 50, 38.

1-(2,4-Dibromophenyl)-2-(1H-1,2,4-triazol-1-yl)ethanone (**13**)

Method A: Yield1.84 g,( 54%), Method B: Yield 2.97 g, (87%), white crystals (ethanol), mp 143–145 °C. Lit. 136–137 °C [45]. Anal. Calcd for C_10_H_7_Br_2_N_3_O C 34.81; H 2.05; Br 46.32; N 12.18%. Found C 34.74; H 2.11; Br 46.18; N 11.99%. MS: calcd for *m*/*z* 344.9, found 344.8, 302, 317, 277, 262, 248, 234, 207, 181, 168, 153, 129, 103, 89, 75, 55, 40. 

1-(2,4-Dichlorophenyl)-2-(1H-1,2,4-triazol-1-yl)ethanone (**14**)

Method A: Yield 1.43 g 56%, Method B: Yield 2.17 g,( 85%), white crystals (MeCN), mp 115–117 °C. Lit. 116–117 °C. [45]. Lit. 106–107 °C [46]. Anal. Calcd for C_10_H_7_Cl_2_N_3_O C 46.90; H 2.76; Cl 27.69; N 16.41%. Found C 46.99; H 2.59; Cl 27.47; N 16.32%.%. MS: calcd for *m*/*z* 256.09, found 255.0, 227, 207, 185, 172, 158, 145, 134, 122, 109, 98, 85, 75, 55, 40.

1-(4-Chlorophenyl)-2-(1H-1,2,4-triazol-1-yl)ethanone (**15**)

Method A: Yield 1.03 g,(47%), Method B: Yield 2.05 g,(93%), white crystals (pentane) mp 156–158 °C. Lit. 143–145 °C [45]. Lit. 121–122 °C [46]. Anal. Calcd for C_10_H_8_ClN_3_O. C 54.19; H 3.64; Cl 16.00; N 18.96%. Found C 54.11; H 3.49; Cl 15.91; N 18.88%. MS: calcd for *m*/*z* 221.64, found 221.0, 207, 193, 165, 152, 139, 125, 111, 98, 85, 75, 63, 50, 40.

4-(2-(4-Methoxyphenyl)-2-oxoethyl)-1-(2-oxo-2-phenylethyl)-1H-1,2,4-triazol-4-ium bromide (**16**)

Yield 3.66 g (88%), white crystals (MeCN), mp 215–216 °C. IR (ν/cm^−1^): 3170, 3130, 3074, 2992, 2930, 2904, 2837, 1978, 1833, 1710, 1699, 1686, 1595, 1585, 1573, 1552, 1504, 1446, 1425, 1361, 1351, 1336, 1272, 1238, 1225, 1182, 1155, 1145, 1137, 1007, 988, 958, 854, 835, 817, 761, 683, 673, 665. ^1^H NMR (DMSO-d6, 400 MHz): 10.17 (1H, s), 9.33 (1H, s), 8.11 (2H, dd, *J* = 1.3, 8.6 Hz), 8.08 (2H, d, *J* = 8.9 Hz), 7.80 (1H, t, *J* = 7.4 Hz), 7.66 (2H, t, *J* = 7.7 Hz), 7.19 (2H, d, *J* = 8.9 Hz), 6.48 (2H, s), 6.25 (2H, s), 3.9 (3H, s). ^13^C NMR (DMSO-d6, 100 MHz): 190.9, 189.1, 164.8, 146.3, 145.7, 135.3, 133.89, 131.2, 129.6, 128.9, 120.7, 114.9, 59, 56.3, 54.4. Calcd for C_19_H_18_BrN_3_O_3_ C 54.82; H 4.36; Br 19.20; N 10.09%. Found C 54.67; H 4.23; Br 19.00; N 10.17%. MS: calcd for *m*/*z* 415.27, found: 382 (M-MeOH)

1-(2-(2,4-Dichlorophenyl)-2-oxoethyl)-4-(2-(4-methoxyphenyl)-2-oxoethyl)-1H-1,2,4-triazol-4-ium bromide (**17**) 

Yield 3.2 g, (66%), white crystals (ethanol), mp 209–211 °C. IR (ν/cm^−1^): 3555, 3381, 3055, 2976, 2924, 2897, 1702, 1688, 1603, 1576, 1553, 1519, 1466, 1423, 1371, 1344, 1324, 1240, 1226, 1213, 1162, 1115, 1102, 1066, 1020, 1004, 980, 828, 806, 753, 744, 707, 663. ^1^H NMR (DMSO-d6, 400 MHz): 10.18 (1H, s), 9.32 (1H, s), 8.14 (1H, d, *J* = 8.5 Hz), 8.07 (2H, d, *J* = 8.8 Hz), 7.89 (1H, d, *J* = 2.0 Hz), 7.73 (1H, dd, *J* = 8.5, 2.0 Hz), 7.19 (2H, d, *J* = 8.8 Hz), 6.38 (2H, s), 6.24 (2H, s), 3.90 (3H, s). ^13^C NMR (DMSO-d6, 100 MHz): 190.7, 189.0, 164.8, 146.4, 145.8, 138.8, 133.3, 132.7, 132.4, 131.4, 131.2, 128.4, 126.7, 114.9, 60.4, 56.3, 54.4. Anal. Calcd for C_19_H_16_BrCl_2_N_3_O_3_ C 47.04; H 3.32; Br 16.47; Cl 14.62; N 8.66%. Found C 47.22; H 3.24; Br 16.55; Cl 14.39; N 8.79%. MS: calcd for *m*/*z* 485.16 found; 404 (M-HBr)

1-(2-(2,4-Dichlorophenyl)-2-oxoethyl)-4-(2-(4-nitrophenyl)-2-oxoethyl)-1H-1,2,4-triazol-4-ium bromide (**18**)

Yield 4.5 g, (90%), white crystals (ethanol), mp 228–229 °C. IR (ν/cm^−1^): 3138, 3091, 3067, 2980, 2903, 2833, 1703, 1689, 1602, 1583, 1573, 1552, 1519, 1433, 1408, 1372, 1346, 1335, 1321, 1286, 1224, 1214, 1168, 1115, 1066, 1002, 983, 886, 854, 814, 751, 745, 687, 659. ^1^H NMR (DMSO-d6, 400 MHz): 10.17 (1H, s), 9.31 (1H, s), 8.48 (2H, d, *J* = 8.8 Hz), 8.33 (2H, d, *J* = 8.8 Hz), 8.13 (1H, d, *J* = 8.5 Hz), 7.90 (1H, d, *J* = 2.0 Hz), 7.74 (1H, dd, *J* = 8.5, 2.0 Hz), 6.38 (2H, s), 6.33 (2H, s). ^13^C NMR (DMSO-d6, 100 MHz): 190.7, 190.2, 151.1, 146.3, 145.8, 138.8, 138.6, 133.3, 132.7, 132.4, 131.5, 130.2, 128.4, 124.7, 60.5, 55.2. Anal. Calcd for C_18_H_13_BrCl_2_N_4_O_4_ C 43.23; H 2.62; Br 15.98; Cl 14.18; N 11.20%. Found C 43.22; H 2.54; Br 16.15; Cl 14.00; N 11.27%. MS: calcd for *m*/*z* 500.13, found: 419 (M-HBr).

1-(2-(2,4-Dichlorophenyl)-2-oxoethyl)-4-(2-oxo-2-phenylethyl)-1H-1,2,4-triazol-4-ium bromide (**19**)

Yield 4.23 g, (93%), white crystals (ethanol), mp 189–202 °C. IR (ν/cm^−1^): 3583, 3402, 3055, 3019, 2964, 2928, 2892, 1986, 1696, 1633, 1595, 1578, 1551, 1532, 1592, 1464, 1446, 1379, 1353, 1343, 1253, 1232, 1210, 1159, 1182, 1103, 1064, 991, 965, 869, 850, 841, 803, 761, 688, 659. ^1^H NMR (DMSO-d6, 400 MHz): 10.19 (1H, s), 9.33 (1H, s), 8.14 (1H, d, *J* = 8.5 Hz), 8.1 (2H, d, *J* = 8.4 Hz), 7.89 (1H, d, *J* = 2.0 Hz), 7.81 (1H, tt, *J* = 5.4, 2.0 Hz), 7.74 (1H, dd *J* = 8.5, 2.0 Hz), 7.68 (2H, t, *J* = 7.4 Hz), 6.39 (2H, s), 6.31 (2H, s). ^13^C NMR (DMSO-d6, 100 MHz): 190.8, 190.7, 146.4, 145.8, 138.8, 135.2, 133.9, 133.3, 132.7, 132.4. 131.4, 129.7, 128.7, 128.4, 60.5, 54.8. Anal. Calcd for C_18_H_14_BrCl_2_N_3_O_2_ C 47.50; H 3.10; Br 17.56; Cl 15.58; N 9.23%. Found C 47.69; H 3.09; Br 17.71; Cl 15.39; N 9.16%. MS: calcd for *m*/*z* 455.13 found: 374.05 (M-HBr)

1,4-Bis(2-(2,4-dichlorophenyl)-2-oxoethyl)-1H-1,2,4-triazol-4-ium bromide (**20**) 

Yield 3.51 g,(67%), white crystals (ethanol), mp 215–216 °C. IR (ν/cm^−1^): 3091, 3003, 2912, 1984, 1726, 1712, 1704, 1583, 1555, 1468, 1391, 1372, 1348, 1339, 1282, 1216, 1183, 1159, 1110, 1078, 1065, 1031, 1010, 991, 931, 866, 844, 812, 738, 713, 659. ^1^H NMR (DMSO-d6, 400 MHz): 10.20 (1H, s), 9.34 (1H, s), 8.14 (2H, m), 7.9 (2H, m), 7.75 (2H, m), 6.39 (2H, s), 6.21 (2H, s). ^13^C NMR (DMSO-d6, 100 MHz): 190.7, 190.3, 146.3, 145.8, 139, 138.8, 133.5, 133.3, 133.1, 132.8, 132.4, 131.6, 131.5, 128.43, 128.36, 60.5, 56.4. Anal. Calcd for C_18_H_12_BrCl_4_N_3_O_2_ C 41.26; H 2.31; Br 15.25; Cl 27.06; N 8.02%. Found C 41.11; H 2.29; Br 15.26; Cl 25.17; N 7.93%. MS: calcd for *m*/*z* 524.02, found 444 (M-HBr)

1-(2-(2,4-Dichlorophenyl)-2-oxoethyl)-4-(2-ethoxy-2-oxoethyl)-1H-1,2,4-triazol-4-ium bromide (**21**)

Yield 3.06 g, (79%), white crystals (ethanol), mp 159–160 °C. IR (ν/cm^−1^): 3156, 3138, 2983, 2936, 1746, 1695, 1583, 1554, 1464, 1435, 1395, 1370, 1351, 1338, 1236, 1207, 1169, 1111, 1074, 1066, 1022, 1003, 984, 891, 864, 815, 766, 725, 658. ^1^H NMR (DMSO-d6, 400 MHz): 10.23 (1H, s), 9.37 (1H, s), 8.12 (1H, d, *J* = 8.5 Hz), 7.88 (1H, d, *J* = 1.9 Hz), 7.72 (1H, dd, *J* = 8.5, 1.9 Hz), 6.36 (2H, s), 5.49 (2H, s), 4.26 (2H, q, *J* = 7.2), 1.27 (3H, t, *J* = 7.2). ^13^C NMR (DMSO-d6, 100 MHz): 190.6, 166.6, 146.2, 145.7, 138.8, 133.3, 132.7, 132.4, 131.4, 128.3. 62.7, 60.5, 49.1, 14.4. Anal. Calcd for C_14_H_14_BrCl_2_N_3_O_3_: C 39.74; H 3.34; Br 18.89; Cl 16.76; N 9.93%. Found C 39.66; H 3.29; Br 19.05; Cl 16.64; N 9.99%. MS: calcd for *m*/*z* 423.09, found. 342(M-HBr)

1-(2-(4-Chlorophenyl)-2-oxoethyl)-4-(2-(2,4-dichlorophenyl)-2-oxoethyl)-1H-1,2,4-triazol-4-ium bromide (**22**)

Yield 4.15 g, (85%), white crystals (ethanol), mp 227–229 °C. IR (ν/cm^−1^): 3555, 3380, 3087, 3067, 2980, 2900, 2833, 1984, 1694, 1601, 1583, 1553, 1519, 1374, 1340, 1322, 1284, 1216, 1170, 1114, 1066, 1000, 985, 864, 852, 815, 744, 737, 685, 658. ^1^H NMR (DMSO-d6, 400 MHz): 9.89 (1H, s), 9.43 (1H, s), 8.55 (1H, s), 8.10 (2H, d, *J* = 8.9 Hz, J = 5.5 Hz), 8.07 (1H, s), 7.69 (2H, d, *J* = 8.9 Hz), 6.36 (2H, s), 6.28 (2H, s). ^13^C NMR (DMSO-d6, 100 MHz): 190.2, 190.0, 147.3, 143.8, 139.0, 138.8, 132.4, 132.2, 133.1, 132.9, 132.1, 131.4, 131.9, 127.66, 128.22, 60.58, 56.2. Anal. Calcd for C_18_H_13_BrCl_3_N_3_O_2_ C, 44.16; H, 2.68; Br, 16.32; Cl, 21.72; N, 8.58%. Found C, 44.14; H, 2.56; Br, 16.19; Cl, 21.59; N, 8.41%. MS: calcd for *m*/*z* 489.58, found 408 (M-HBr)

1-(2-(2,4-Dichlorophenyl)-2-oxoethyl)-4-ethyl-1H-1,2,4-triazol-4-ium iodide (**23**)

Yield 2.35 g, (65%), white crystals (ethanol), mp 170–171 °C. IR (ν/cm^−1^): 1150, 1125, 3028, 2980, 2916, 2813, 2753, 1779, 1688, 1583, 1573, 1551, 1524, 1478, 1465, 1408, 1369, 1340, 1320, 1281, 1211, 1156, 1112, 1064, 1005, 984, 938, 897, 961, 814, 727, 705, 671, 665. ^1^H NMR (DMSO-d6, 400 MHz): 10.16 (1H, s), 9.40 (1H, s), 8.09 (1H, d, *J* = 8.5 Hz), 7.88 (1H, d, *J* = 2.0 Hz), 7.72 (1H, dd, *J* = 8.5, 2.0 Hz), 6.24 (2H, s), 4.42 (2H, q, *J* = 7.3 Hz), 1,51 (3H, t, *J* = 7.3 Hz). ^13^C NMR (DMSO-d6, 100 MHz): 190.8, 145.1, 144.5, 138.8, 133.2, 132.7, 132.5, 131.4, 128.4, 60.1, 43.1, 15.1. Anal. Calcd for C_12_H_12_Cl_2_IN_3_O C 34.98; H 2.94; Cl 17.21; N 10.20%. Found C 35.11; H 3.23; Cl 17.39; N 10.34%. MS: calcd for *m*/*z* 365.05, found 364 

1,4-Dibenzyl-1H-1,2,4-triazol-4-ium chloride (**24**)

Yield 1.68 g, (59%), white crystals (ethanol), mp 184–185 °C. IR (ν/cm^−1^): 3547, 3162, 3051, 2972, 2902, 2833, 2325, 1704, 1688, 1649, 1601, 1573, 1553, 1519, 1480, 1464, 1423, 1408, 1374, 1344, 1322, 1288, 1229, 1175, 1155, 1115, 1066, 132, 1004, 985, 949, 889, 850, 830, 809, 745, 715, 686, 659. ^1^H NMR (DMSO-d6, 400 MHz); 10.51 (1H, s), 9.40 (1H, s), 7.53 (2H, d, *J* = 7.7 Hz), 7.44 (8H, m), 5.64 (2H, s), 5.56 (2H, s). ^13^C NMR (DMSO-d6, 100 MHz):145.5, 143.3, 134, 133.7, 129.6, 129.3, 55.3, 51.1. Anal. Calcd for C_16_H_16_ClN_3_ C 67.25; H 5.64; Cl 12.41; N 14.70%. Found C 67.29; H 5.66; Cl 12.56; N 14.88%. MS: calcd for *m*/*z* 330.22, found: 250 (M- HBr)

1-Benzyl-4-(2-oxo-2-phenylethyl)-1H-1,2,4-triazol-4-ium bromide (**25**) 

Yield 2.31 g, (74%), white crystals (ethanol), mp 206–207 °C. IR (ν/cm^−1^): 3170, 3103, 3058, 3039, 2905, 2869, 2837, 1700, 1583, 1569, 1552, 1520, 1478, 1455, 1446, 1433, 1397, 1367, 1338, 1278, 1240, 1215, 1172, 1155, 1144, 1109, 1072, 1030, 994, 965, 945, 919, 897, 868, 848, 818, 806, 779, 761, 723, 696, 685. ^1^H NMR (DMSO-d6, 400 MHz): 10.61 (1H, s), 9.45 (1H, s), 8.10 (2H, d, *J* = 7.3 Hz), 7.78, (1H, t, *J* = 7.4 Hz), 7.64 (2H, t, J = 7.8 Hz), 7.55 (2H, d, *J* = 6. 8 Hz), 7.46 (2H, t, *J* = 6.8 Hz), 7.42 (1H, t, *J* = 6.7 Hz), 6.42, (2H, s), 5.90 (2H, s). ^13^C NMR (DMSO-d6, 100 MHz):191.0, 146.5, 144.1, 135.2, 133.84, 133.8, 129.7, 129.5, 129.4, 129.3, 128.8, 55.3, 54.9. Anal. Calcd for C_17_H_16_BrN_3_O_:_ C 57.00; H 4.50; Br 22.31; N 11.73%. Found C 57.15; H 4.29; Br 22.49; N 11.96%. MS: calcd for *m*/*z* 360.25, found: 278 (M-HBr)

1-Benzyl-4-(2-(2,4-dichlorophenyl)-2-oxoethyl)-1H-1,2,4-triazol-4-ium bromide (**26**) 

Yield 3.09 g, (81%), white crystals (ethanol), mp 215–216 °C. IR (ν/cm^−1^): 3174, 3134, 3063, 2988, 2936, 2841, 1756, 1712, 1698, 1597, 1582, 1567, 1555, 1449, 1427, 1374, 1346, 1337, 1274, 1215, 1183, 1155, 1115, 1072, 1005, 995, 981, 955, 922, 856, 838, 818, 801, 760, 753, 686, 771, 662. ^1^H NMR (DMSO-d6, 400 MHz): 10.09 (1H, s), 9.26 91H, s), 8.11 (3H, m), 7.93 (1H, d, *J* = 2.0 Hz), 7.80 (1H, t, *J* = 7.4 Hz), 7.76 (1H, dd, *J* = 2.0, 8.5 Hz), 7.67 (2H, t, *J* = 7.7 Hz), 6.43 (2H, s), 6.14 (2H, s). ^13^C NMR (DMSO-d6, 100 MHz): 190.3, 172.5, 146.1, 138.9, 135.2, 133.9, 133.5, 133, 132.1, 131.5, 129.6, 128.8, 128.4, 59, 56.4. Anal. Calcd for C_17_H_14_BrCl_2_N_3_O C 47.80; H 3.30; Cl 16.60; N 9.84%. Found C 47.97; H 3.24; Cl 16.59; N 10.01%. MS: calcd for *m*/*z* 427.12, found 346 (M-HBr)

### 3.2. Antifungal Action

The following fungi were used, including Aspergillus niger (ATCC 6275), Aspergillus fumigatus (ATCC 1022), Aspergillus versicolor (ATCC 11730), Penicillium funiculosum (ATCC 36839), Trichoderma viride (IAM 5061), Penicillium verrucosum var. cyclopium (food isolate). The organisms were obtained from the Mycological Laboratory, Department of Plant Physiology, Institute for Biological Research—Siniša Stankovic, Belgrade, Serbia.

The antifungal activity of the synthesized compounds was determined by the modified microdilution method [47,48]. Prior to the antifungal assay, fungi were cultured for 14 days on solid malt agar at 25 °C, after which the spore inoculum was prepared. Spores were washed using sterile saline and 0.1% Tween 80 (*v*/*v*), and the suspension was adjusted to a concentration of approximately 1.0 × 10^5^ CFU in a final volume of 100 µL per well by microscopic enumeration with a cell-counting hematocytometer (Neubauer chamber; Paul Marienfeld, England). The inocula were stored at 4 °C until further use. The obtained results were presented as minimal inhibitory (MICs), and minimal fungicidal concentrations (MFCs) needed to effectively retard fungal growth. The commercially available antifungal agents ketoconazole and bifonazole were used as positive controls. The ethanol solution (30%) was used as a negative control. All experiments were performed in duplicate and repeated three times.

### 3.3. In-Silico Molecular Docking Studies

Docking studies were performed in an effort to predict the mechanism of action of the compounds. All calculations were performed using the Autodock 4.2 program [49]. To prepare the proteins, all water molecules were eliminated, and polar hydrogens were added, while for the preparation of the inhibitors, charges were added, and rotatable bonds were determined. Grid maps have been calculated utilizing the Autogrid algorithm and must contain the area to be connected. The Autogrid Box was computed by the X-, Y-, and Z-coordinates for each enzyme. Three-dimensional structures of all compounds were constructed using Chem3Dultra 12.0 software (Chemical Structure Drawing Standard; Perkin Elmer Informatics, Waltham, MA, USA).

For the present system, the following settings were used: initial population: 300, 2,500,000 maximum energy ratings, and 27,000 as a maximum generation. The pitch was 1,0 Å, while the quaternion and pivot angles were set to 5.0 degrees. For each compound, 200 configurations were produced. The results from the Autodock calculations were grouped using a RMSD deviation value of 1.5 Å, while the lowest-energy configuration of the largest population group was chosen as the most likely tethering configuration. The discovery studio 2017 R2 silent and LigandScout (Vienna, Austria) were used to display the results and process the configurations with the highest tie rating [50].

**Figure 8:**
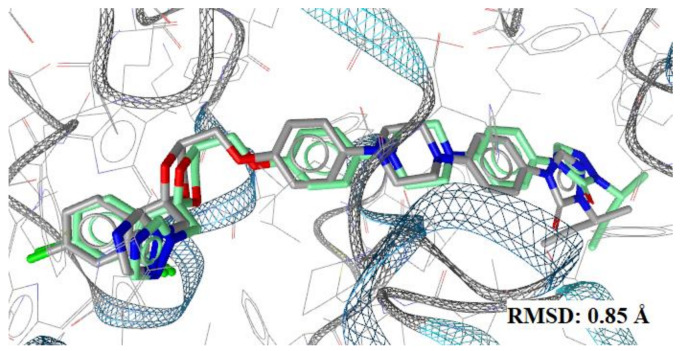
Docking of the initial inhibitor to the 5V5Z structure.

For antifungal activity, lanosterol 14a-demethylase, CYP51, an enzyme from *C. albicans* with PDB I.D. 5V5Z, and DNA topoisomerase IV with PDB I.D. 1S16, were chosen for docking studies. The docking box was centered on the heme molecule, at the active center of the enzyme, with coordinates x = −47.731, y = −13.422, z = 22.982 with a target box of 50 × 50 × 50Å. As a first step of docking studies, the initial inhibitor 2-[(2R)-butan-2-yl]-4-{4-[4-(4-{[(2R,4S)-2-2,4-dichlorophenyl-2-(1H-1,2,4-triazol-1-ylmethyl)-1,3-dioxolan-4-yl]methoxy}phenyl)piperazin-1-yl]phenyl}-2,4-dihydro-3H-1,2,4-triazol-3-one, was removed and docked to the prepared enzyme for verification of the method (Figure 8) with an RMSD value of 0.855Å. Furthermore, the reference drug ketoconazole was also docked into the active site of 5V5Z structure.

### 3.4. In Silico Predictive Studies 

Drug-likeness is one of the qualitative ideas employed for predicting a drug-like property. It is designated as an intricate balance of diverse molecular and structural features, which plays a pivotal task in establishing if the specific drug candidate can be likened to the known drugs. The targeted molecules were appraised for predicting the Drug-likeness based on five separate filters, namely the Egan [51], Ghose [52], Veber [53], Muegge [54], and Lipinski [55] rules accompanying bioavailability, and the drug-likeness scores using the Molsoft software and SwissADME program(http://swissadme.ch, access on 11 January 2022) using the ChemAxon’s Marvin JS structure drawing tool.

## 4. Conclusions

Fifteen triazolium salts were synthesized and evaluated for their antifungal activity against a panel of eight fungi. All compounds showed good antifungal activity with MIC /MFC in the range of 0.0003–0.2 /0.0006–0.4 mg/mL. As reference compounds, ketoconazole and bifonazole were used. The most active compound (**19**) exhibited MIC/MFC in the range of 0.009–0.037 /0.0125–0.05 mg/mL, respectively. All compounds appeared to be more active than ketoconazole and bifonazole. Thus, compound **19** showed 209 times superior activity than ketoconazole and 53-fold more than bifonazole against *T. viride,* while compound **20** was 1267 times more potent than ketoconazole and 1067 than bifonazole against *A. versicolor*.

Docking to DNA TopoIV and CYP51 of *C. albicans* revealed that the best estimated binding energy was shown by CYP51, indicating the probable involvement of this enzyme in the mechanism of antifungal activity. The docking results revealed that the most active compound **19** takes place inside the enzyme interacting with the heme group of the enzyme throughout its benzene ring, thus forming aromatic interactions in the same way with ketoconazole.

This in silico prediction study indicated that none of the compounds violated any rule, and their bioavailability score was approximately 0.55.

## Figures and Tables

**Table 1:** Antifungal activity of the synthesized compound (MIC and MFC are in mg/mL).

Compounds		*A.fum.*	*A.v.*	*A.o.*	*A.n.*	*T.v.*	*P.f.*	*P.o.*	*P.v.c.*
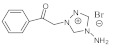 **6**	MIC	0.15	0.10	0.10	0.05	0.025	0.10	0.07	0.20
	MFC	0.20	0.20	0.20	0.10	0.05	0.20	0.10	0.40
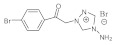 **7**	MIC	0.025	0.025	0.0125	0.025	0.05	0.05	0.025	0.05
	MFC	0.05	0.05	0.025	0.05	0.10	0.10	0.05	0.10
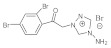 **8**	MIC	0.025	0.0125	0.07	0.0	0.009	0.0125	0.0125	0.025
	MFC	0.05	0.025	0.10	0.10	0.0125	0.025	0.025	0.05
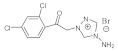 **9**	MIC	0.05	0.05	0.025	0.037	0.015	0.03	0.025	0.05
	MFC	0.10	0.10	0.05	0.0	0.025	0.05	0.05	0.10
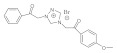 **16**	MIC	0.07	0.006	0.05	0.025	0.025	0.05	0.05	0.10
	MFC	0.10	0.0125	0.10	0.05	0.05	0.10	0.10	0.20
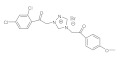 **17**	MIC	0.037	0.015	0.025	0.05	0.009	0.037	0.037	0.037
	MFC	0.05	0.025	0.05	0.10	0.0125	0.05	0.05	0.05
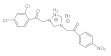 **18**	MIC	0.05	0.03	0.025	0.05	0.015	0.05	0.03	0.07
	MFC	0.10	0.05	0.05	0.10	0.025	0.10	0.05	0.10
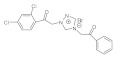 **19**	MIC	0.037	0.0125	0.025	0.037	0.009	0.02	0.0125	0.025
	MFC	0.05	0.025	0.05	0.05	0.0125	0.025	0.025	0.05
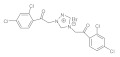 **20**	MIC	0.07	0.0003	0.05	0.05	0.006	0.037	0.025	0.025
	MFC	0.10	0.0006	0.10	0.10	0.0125	0.05	0.05	0.05
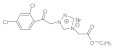 **21**	MIC	0.05	0.05	0.025	0.025	0.012	0.05	0.037	0.025
	MFC	0.10	0.10	0.05	0.05	0.025	0.10	0.05	0.05
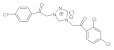 **22**	MIC	0.05	0.015	0.037	0.05	0.009	0.037	0.025	0.027
	MFC	0.10	0.025	0.05	0.10	0.0125	0.05	0.05	0.05
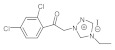 **23**	MIC	0.10	0.07	0.05	0.05	0.037	0.07	0.05	0.07
	MFC	0.20	0.10	0.10	0.10	0.05	0.10	0.10	0.10
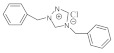 **24**	MIC	0.10	0.05	0.037	0.05	0.015	0.05	0.05	0.10
	MFC	0.20	0.10	0.05	0.10	0.05	0.10	0.10	0.20
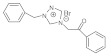 **25**	MIC	0.10	0.07	0.037	0.05	0.025	0.20	0.025	0.20
	MFC	0.20	0.10	0.05	0.10	0.05	0.40	0.05	0.40
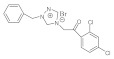 **26**	MIC	0.10	0.037	0.05	0.05	0.015	0.037	0.037	0.037
	MFC	0.20	0.05	0.10	0.10	0.025	0.05	0.05	0.05
**Ketoconazole**	MIC	0.38	0.38	0.28	0.38	1.88	0.38	1.88	0.38
	MFC	0.94	0.94	0.38	0.94	2.82	0.94	2.82	0.57
**Bifonazole**	MIC	0.48	0.32	0.48	0.48	0.48	0.64	0.64	0.32
	MFC	0.64	0.64	0.64	0.64	0.64	0.81	0.81	0.64

*A.f.—A. fumigatus, A.v.—A. versicolor, A.o.—A. ochraceus, A.n.—A. niger, T.v.—T. viride, P.f.—P. funiculosum, P.o.—P. ochrochloron, P.v.c.—P. cyclpoium var verucosum.*

**Table 2:** Molecular docking free binding energy estimates to antifungal targets.

	Est. Binding Energy (kcal/mol)					
N/N	DNA TopoIV 1S16	CYP51 of *C. albicans* 5V5Z	Residues Involved in H-Bond Interactions	Halogen Interactions	Hydrophobic Interactions	Interactions with HEM601
**6**	-	−6.60	-	-	Phe233, Leu300, Phe380, Met508, Hem601	Hydrophobic
**7**	−4.11	−9.27	Tyr132	-	Phe233, Leu376, Phe380, Met508, Val509, Hem601	Hydrophobic, Aromatic
**8**	−2.27	−10.60	-	Met508	Phe228, Thr311, Leu376, Met508, Val509	Negative ionizable
**9**	−3.37	−9.10	Tyr118	-	Leu376, Phe380, Met508, Val509, Hem601	Hydrophobic, Aromatic
**16**	−2.82	−6.95	-	-	Leu376, Met508, Hem601	Hydrophobic
**17**	−1.86	−8.80	Tyr118	-	Tyr118, Leu376, Met508, Hem601	Hydrophobic, Aromatic
**18**	−4.16	−8.84	Tyr64	-	Tyr118, Leu300, Leu376, Phe380, Met508, Hem601	Hydrophobic, Aromatic
**19**	−3.30	−10.83	Tyr132	-	Phe233, Thr311, Leu376, Phe380, Met508, Hem601	Hydrophobic, Aromatic, Positive ionizable
**20**	-	−7.11	-	-	Thr311, Leu376, Met508, Hem601	Hydrophobic
**21**	-	−8.10	-	Met508	Thr311, Leu376, Met508, Hem601	Hydrophobic, Aromatic
**22**	−2.52	−9.15	Tyr132	-	Thr311, Leu376, Met508, Hem601	Hydrophobic, Aromatic
**23**	−3.79	−7.50	Tyr118	-	Tyr118, Leu376, Hem601	Hydrophobic
**24**	-	−9.73	-	Met508	Phe233, Thr311, Leu376, Met508, Hem601	Hydrophobic, Aromatic
**25**	-	−10.20	Tyr132	-	Thr311, Leu376, Phe380, Met508, Hem601	Hydrophobic
**26**	−4.53	−7.72	-	-	Phe233, Leu376, Phe380, Met508, Hem601	Hydrophobic
**Ket.**	-	−8.23	Tyr64	-	Tyr118, Ile131, Tyr132, Ile304, Leu300, Leu376, Met508, Hem601	Hydrophobic, Aromatic

**Table 3:** Molecular docking free binding energy estimates to 14-alpha demethylase (CYP51B) from the fungus *Aspergillus fumigatus* in complex with voriconazole.

N/N	Est. Binding Energy(kcal/mol) CYP51 of *Aspergillus fumigatus* 4UYM	Residues Involved in H-Bond Interactions	Hydrophobic Interactions	Interactions with HEM601
**6**	−7.15	-	Tyr122, Thr126, Val135, Ala307, Phe229, Ala303, Leu503, Hem580	Hydrophobic
**7**	−10.30	Hem508	Phe130, Val135, Ala307, Ala303, Leu304	Hydrogen bond
**8**	−11.47	Tyr122, Hem508	Thr126, Phe130, Val135, Ala307, Ala303, Leu503, Hem580	Hydrophobic, hydrogen bond
**9**	−9.27	Tyr122	Thr126, Val135, Ala307, Ala303, Leu503, Hem580	Hydrophobic, Aromatic
**16**	−7.91	-	Tyr122, Thr126, Phe130, Ala307, Phe229, Ala303, Ile373, Phe504, Hem580	Hydrophobic
**17**	−10.45	Hem508	Tyr122, Phe130, Val135, Ala307, Leu304	Hydrogen bond
**18**	−8.86	-	Phe130, Val135, Ala307, Ala303, Leu304, Hem580	Hydrophobic, Aromatic
**19**	−10.82	Hem508	Tyr122, Thr126, Phe130, Val135, Ile373, Phe504, Hem580	Hydrophobic, hydrogen bond
**20**	−8.05	-	Tyr122, Thr126, Phe130, Ala307, Hem580	Hydrophobic
**21**	−8.30	-	Tyr122, Thr126, Phe130, Ala303, Phe504, Hem580	Hydrophobic, Aromatic
**22**	−9.02	Tyr122	Thr126, Val135, Ala307, Leu503, Hem580	Hydrophobic, Aromatic
**23**	−7.24	-	Tyr122, Thr126, Ala307, Phe229, Ala303, Hem580	Hydrophobic
**24**	−8.41	-	Thr126, Phe130, Ala303, Phe504, Hem580	Hydrophobic, Aromatic
**25**	−8.80	-	Phe130, Val135, Ala307, Leu304, Hem580	Hydrophobic, Aromatic
**26**	−7.10	-	Tyr122, Thr126, Val135, Hem580	Hydrophobic
**voriconazole**	−10.23	-	Tyr122, Thr126, Phe130, Val135, Ala307, Phe229, Ala303, Ile373, Phe504, Leu503, Hem580	Hydrophobic, Fe-binding

**Table 4:** Drug likeness predictions and physicochemical–pharmacokinetic/ADME properties of the tested compounds.

No	MW	Number of HBA ^a^	Number of HBD ^b^	Log *P*_o/w_ (iLOGP) ^c^	Log S ^d^	TPSA ^e^	Lipinski, Ghose, Veber, Egan, and Muegge Violations	Bioavailability Score	Drug-Likeness Model Score
**6**	285.14	5	1	−14.56	Soluble	49.57	0	0.55	−0.94
**7**	364.04	5	1	−10.84	Soluble	49.57	0	0.55	−0.81
**8**	442.93	5	1	−9.33	Soluble	49.57	0	0.55	−0.62
**9**	354.03	5	1	−10.27	Soluble	49.57	0	0.55	−0.45
**16**	418.28	6	0	−9.26	Soluble	49.85	0	0.55	−0.69
**17**	487.17	6	0	−7.33	Poorly Soluble	49.85	0	0.55	0.05
**18**	504.16	6	1	0.00	Moderately Soluble	103.05	1	0.55	−0.27
**19**	457.15	5	0	−7.94	Moderately Soluble	40.62	0	0.55	−0.01
**20**	526.04	5	0	−6.96	Poorly Soluble	40.62	1	0.55	−0.44
**21**	425.11	6	0	−9.97	Moderately Soluble	49.85	0	0.55	−0.32
**22**	447.14	5	0	−7.91	Poorly Soluble	40.62	0	0.55	−0.23
**23**	414.07	4	0	−1.63	Moderately Soluble	23.55	0	0.55	−0.19
**24**	287.79	3	0	−8.77	Moderately Soluble	6.48	0	0.55	−1.15
**25**	360.25	4	0	−9.78	Moderately Soluble	23.55	0	0.55	−0.70
**26**	429.14	4	0	−8.52	Poorly Soluble	23.55	0	0.55	0.17

(a) Number of hydrogen bond acceptors; (b) number of hydrogen bond donors; (c) lipophilicity; (d) water solubility (SILICOS-IT [S = Soluble]); (e) topological polar surface area (Å^2^).

## Supplementary Materials

The following spectra are presented at: https://www.mdpi.com/article/10.3390/antibiotics11050588/s1. IR, ^1^H-NMR, ^13^C-NMR, HPLC and MS.
