# Supplementary material for: The Synthesis of Triazolium Salts as Antifungal Agents: A Biological and In Silico Evaluation
Source: Antibiotics (Basel). 2022 Apr 27;11(5):588. doi: 10.3390/antibiotics11050588 (PMC9137982; doi:10.3390/antibiotics11050588)

Triazolium salts as antifungal agents. Synthesis, biological and in *silico* evaluation

Serghei Pogrebnoi[1,2], Oleg Radul[1], Eugenia Stingaci[1], Lucian Lupascu[1], Vladimir Valica[2], Livia Uncu[2], Anastasia Smetanscaia[2], Anthi Petrou[3], Ana Ciric[4], Jasmina Glamoclija[4], Marina Sokovic[4], Athina Geronikaki*[3], Fliur Z. Macaev*[1,2]

[1] Laboratory of Organic Synthesis, Institute of Chemistry, Chisinau, 3 str. Academiei, Moldova;
[2] Scientific Center for Drug Research, "Nicolae Testemitanu" State University of Medicine and Pharmacy, Chisinau, Moldova.
[3] Department of Pharmacy School of Health, Department of Pharmacy, Aristotle University of Thessaloniki, Thessaloniki, 54124, Greece.
[4] Mycological Laboratory, Department of Plant Physiology, Institute for Biological Research, Siniša Stanković, University of Belgrade, Bulevar Despota Stefana 142, 11000, Belgrade, Serbia.

* Correspondence: geronik@pharm.auth.gr; Tel.:+302301997616

Received: date; Accepted: date; Published: date

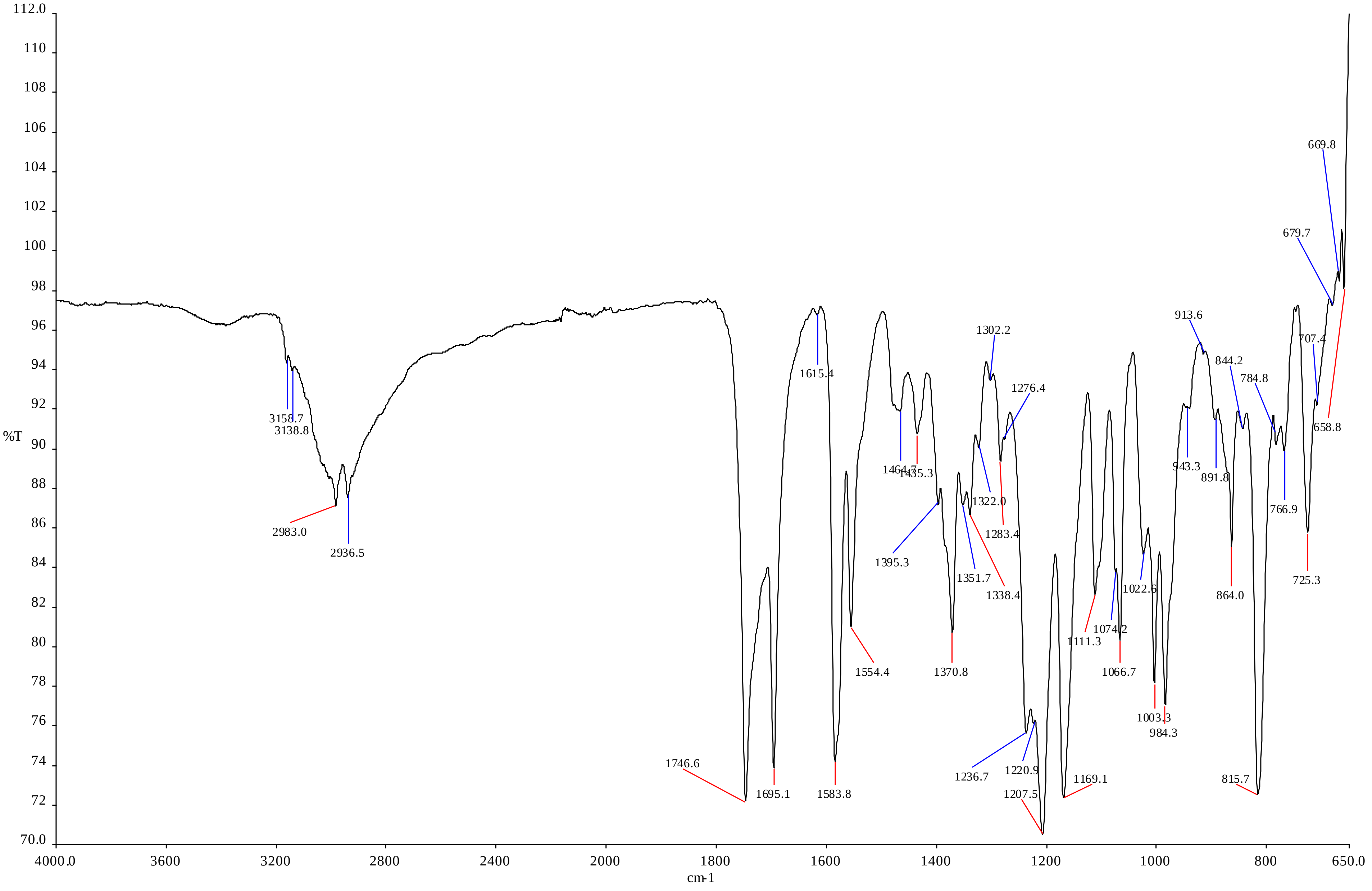

%T
cm-1
3158.7
3138.8
2983.0
2936.5
1746.6
1695.1
1615.4
1583.8
1554.4
1464.7
1435.3
1395.3
1370.8
1351.7
1338.4
1322.0
1302.2
1283.4
1276.4
1236.7
1220.9
1207.5
1169.1
1111.3
1074.2
1066.7
1022.6
1003.3
984.3
943.3
913.6
891.8
864.0
844.2
815.7
784.8
766.9
725.3
707.4
679.7
669.8
658.8

21

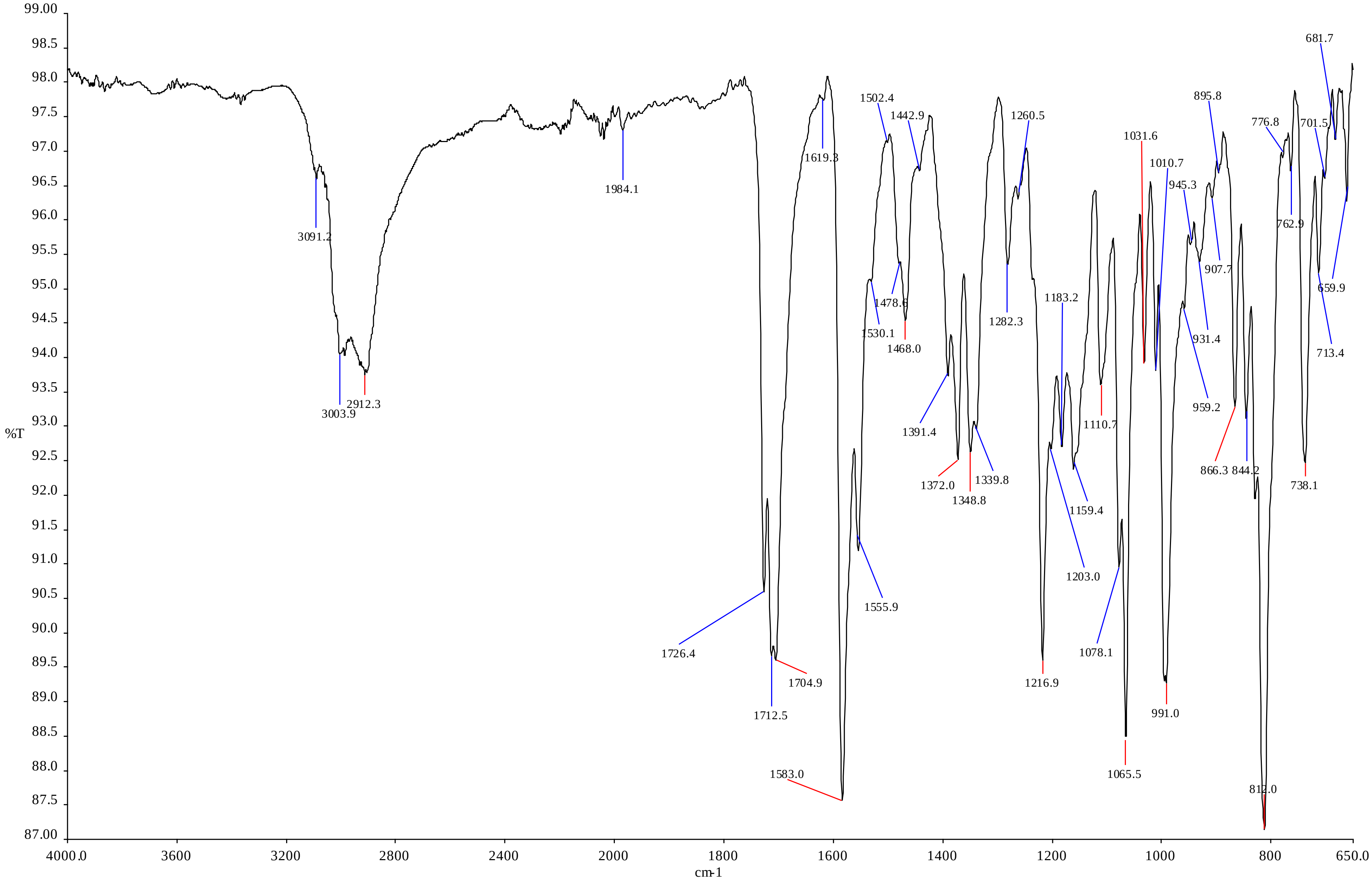
%T
cm-1
3091.2
3003.9
2912.3
1984.1
1619.3
1502.4
1442.9
1260.5
1031.6
1010.7
945.3
895.8
776.8
701.5
681.7
762.9
907.7
659.9
1478.6
1530.1
1468.0
1282.3
1183.2
931.4
713.4
1391.4
1110.7
959.2
1372.0
1348.8
1339.8
1159.4
866.3
844.2
738.1
1203.0
1555.9
1726.4
1712.5
1704.9
1216.9
1078.1
991.0
1583.0
1065.5
812.0

20

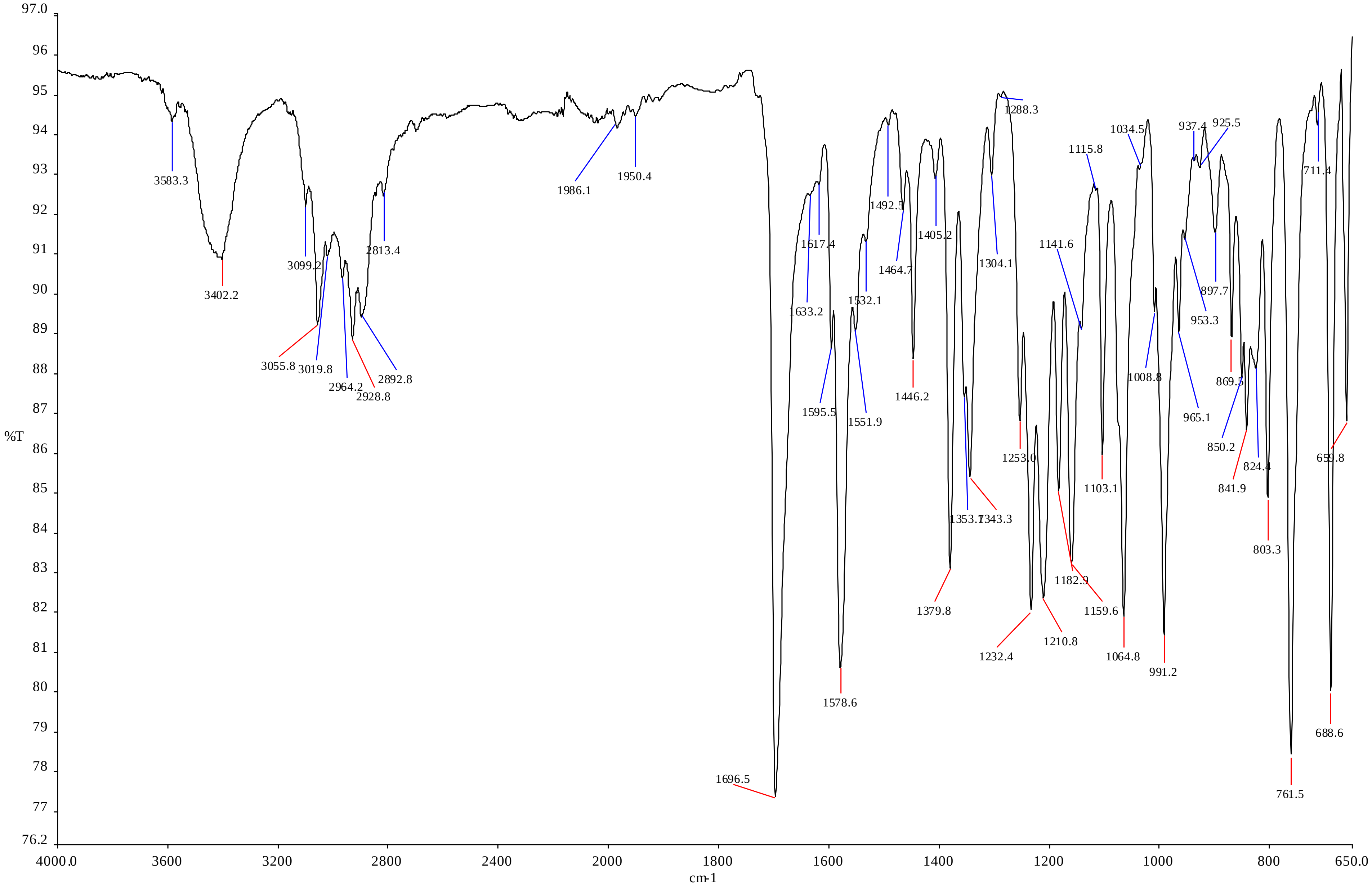
%T
cm-1
97.0
96
95
94
93
92
91
90
89
88
87
86
85
84
83
82
81
80
79
78
77
76.2
4000.0
3600
3200
2800
2400
2000
1800
1600
1400
1200
1000
800
650.0
3583.3
3402.2
3099.2
3055.8
3019.8
2964.2
2928.8
2892.8
2813.4
1986.1
1950.4
1696.5
1633.2
1617.4
1595.5
1578.6
1551.9
1532.1
1492.5
1464.7
1446.2
1405.2
1379.8
1353.7
1343.3
1304.1
1288.3
1253.0
1232.4
1210.8
1182.9
1159.6
1141.6
1115.8
1103.1
1064.8
1034.5
1008.8
991.2
965.1
953.3
937.4
925.5
897.7
869.5
850.2
841.9
824.4
803.3
761.5
711.4
688.6
659.8

19

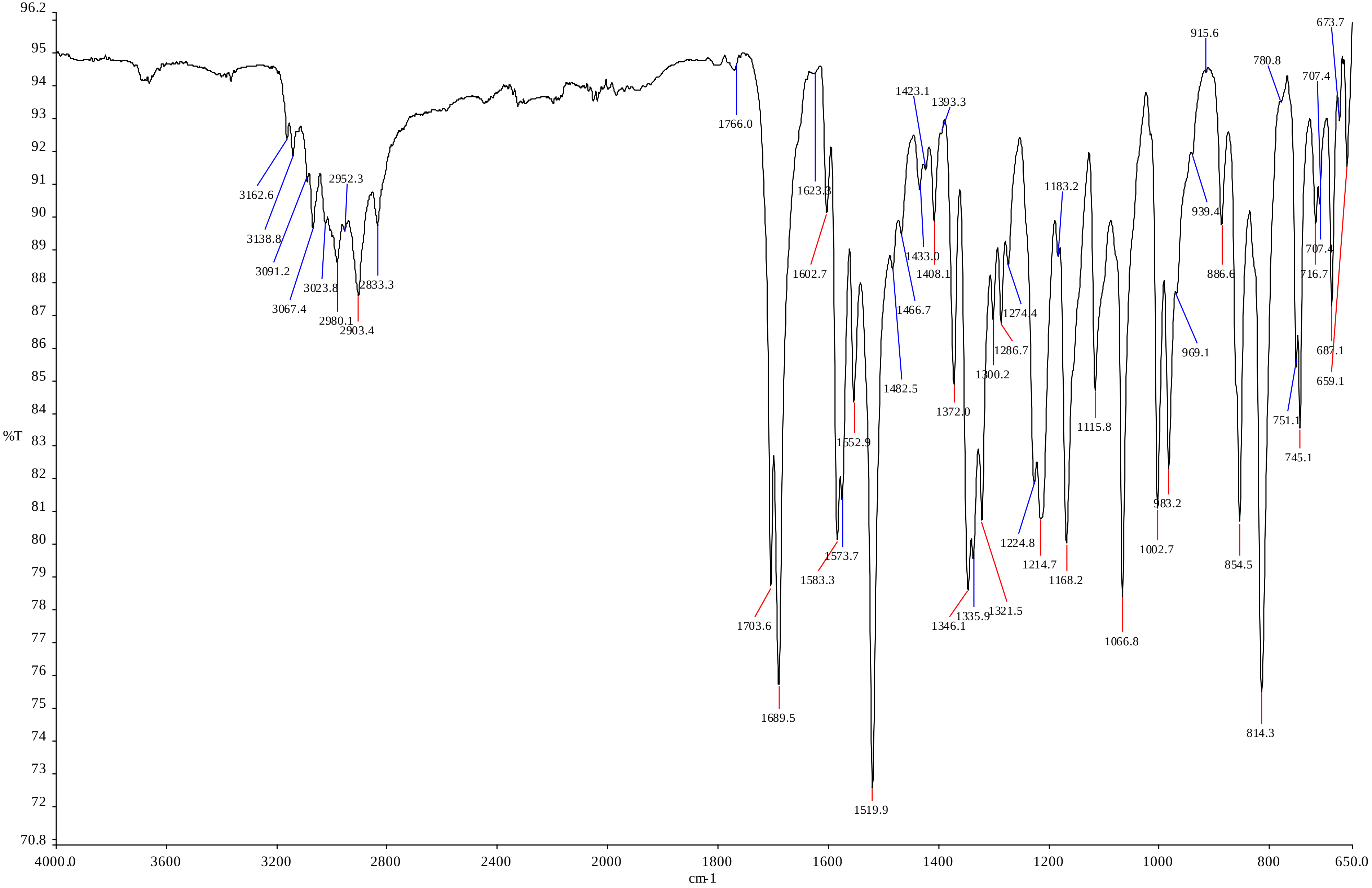
%T
cm-1
4000.0
650.0
96.2
70.8
1766.0
1689.5
1519.9
814.3
1066.8
1703.6
1623.3
1602.7
1583.3
1573.7
1552.9
1482.5
1466.7
1433.0
1423.1
1408.1
1393.3
1372.0
1346.1
1335.9
1321.5
1300.2
1286.7
1274.4
1224.8
1214.7
1183.2
1168.2
1115.8
1002.7
983.2
969.1
939.4
915.6
886.6
854.5
780.8
751.1
745.1
716.7
707.4
687.1
673.7
659.1
3162.6
3138.8
3091.2
3067.4
3023.8
2980.1
2952.3
2903.4
2833.3

18

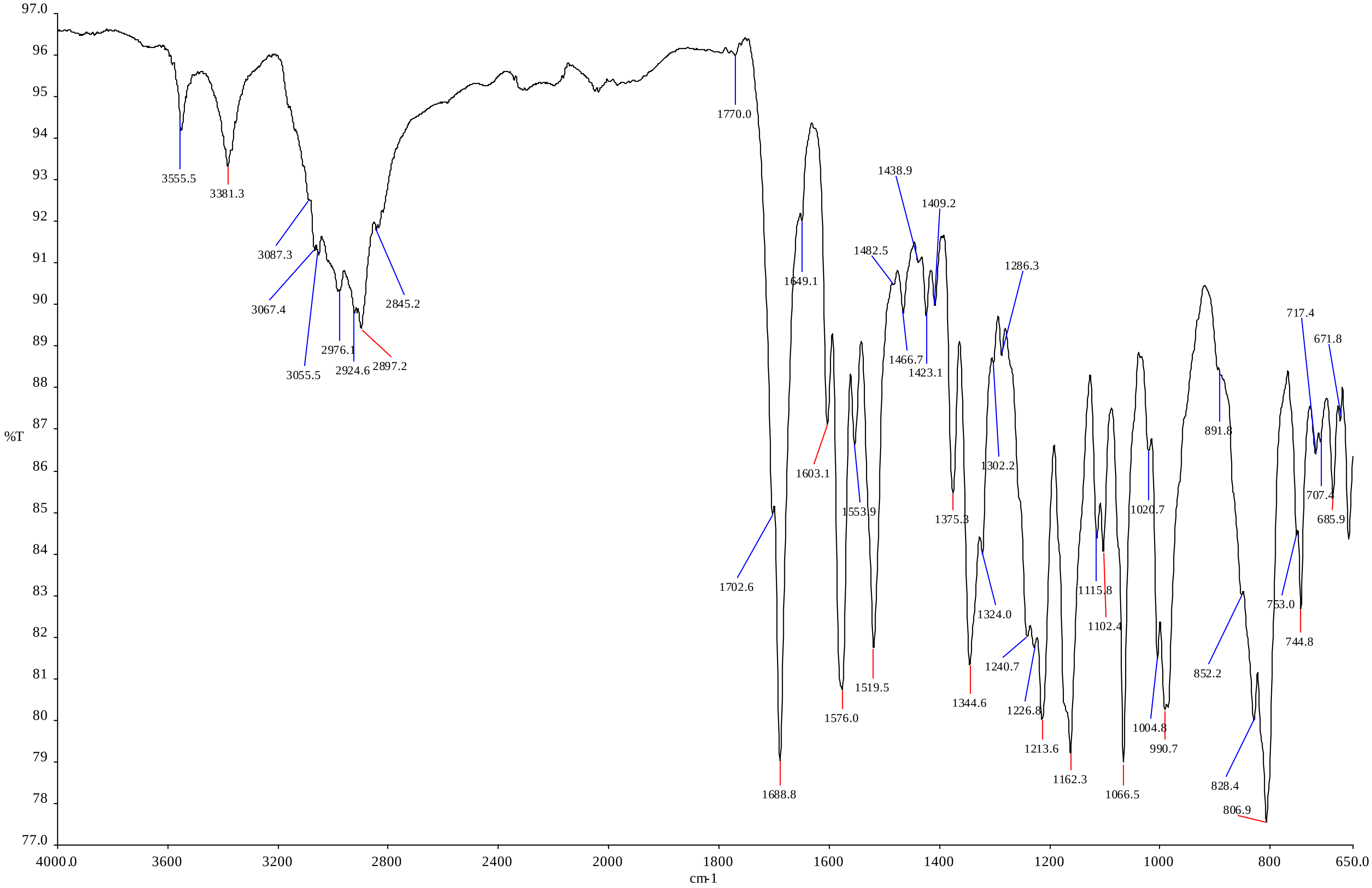
%T
cm-1
3555.5
3381.3
3087.3
3067.4
3055.5
2976.1
2924.6
2897.2
2845.2
1770.0
1702.6
1688.8
1649.1
1603.1
1576.0
1553.9
1519.5
1482.5
1466.7
1438.9
1423.1
1409.2
1375.3
1344.6
1324.0
1302.2
1286.3
1240.7
1226.8
1213.6
1162.3
1115.8
1102.4
1066.5
1020.7
1004.8
990.7
891.8
852.2
828.4
806.9
753.0
744.8
717.4
707.4
685.9
671.8

17

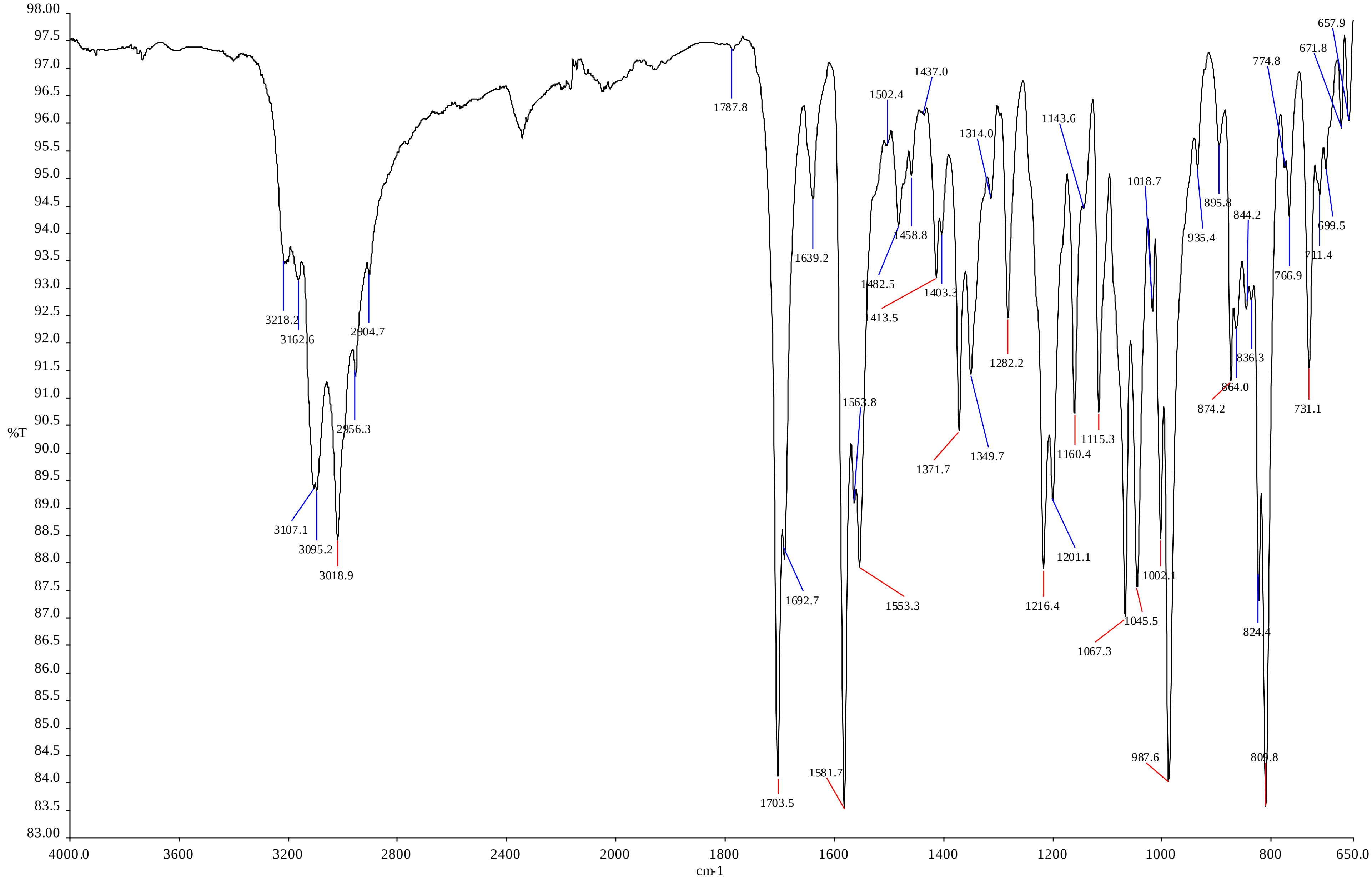
%T
cm-1
98.00
83.00
4000.0
3600
3200
2800
2400
2000
1800
1600
1400
1200
1000
800
650.0
3218.2
3162.6
3107.1
3095.2
3018.9
2956.3
2904.7
1787.8
1703.5
1692.7
1639.2
1581.7
1563.8
1553.3
1502.4
1482.5
1458.8
1437.0
1413.5
1403.3
1371.7
1349.7
1314.0
1282.2
1216.4
1201.1
1160.4
1143.6
1115.3
1067.3
1045.5
1018.7
1002.1
987.6
935.4
895.8
874.2
864.0
844.2
836.3
824.4
809.8
774.8
766.9
731.1
711.4
699.5
671.8
657.9

9

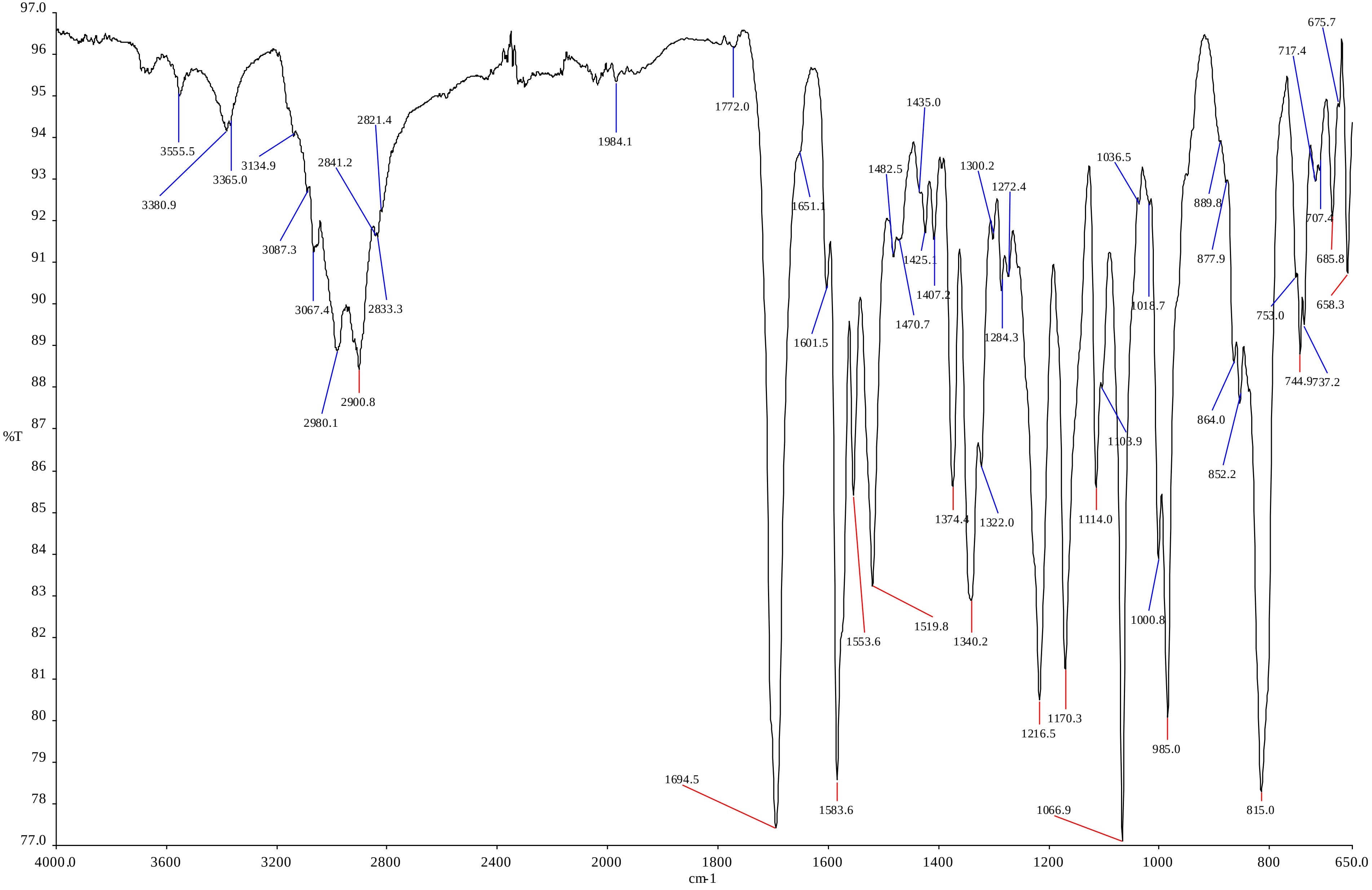
97.0
96
95
94
93
92
91
90
89
88
87
86
85
84
83
82
81
80
79
78
77.0
%T
4000.0
3600
3200
2800
2400
2000
1800
1600
1400
1200
1000
800
650.0
cm-1
3555.5
3380.9
3365.0
3134.9
3087.3
3067.4
2980.1
2900.8
2841.2
2821.4
2833.3
1984.1
1772.0
1694.5
1651.1
1601.5
1583.6
1553.6
1519.8
1482.5
1470.7
1435.0
1425.1
1407.2
1374.4
1340.2
1322.0
1300.2
1284.3
1272.4
1216.5
1170.3
1114.0
1103.9
1066.9
1036.5
1018.7
1000.8
985.0
889.8
877.9
864.0
852.2
815.0
753.0
744.9
737.2
717.4
707.4
685.8
675.7
658.3

22

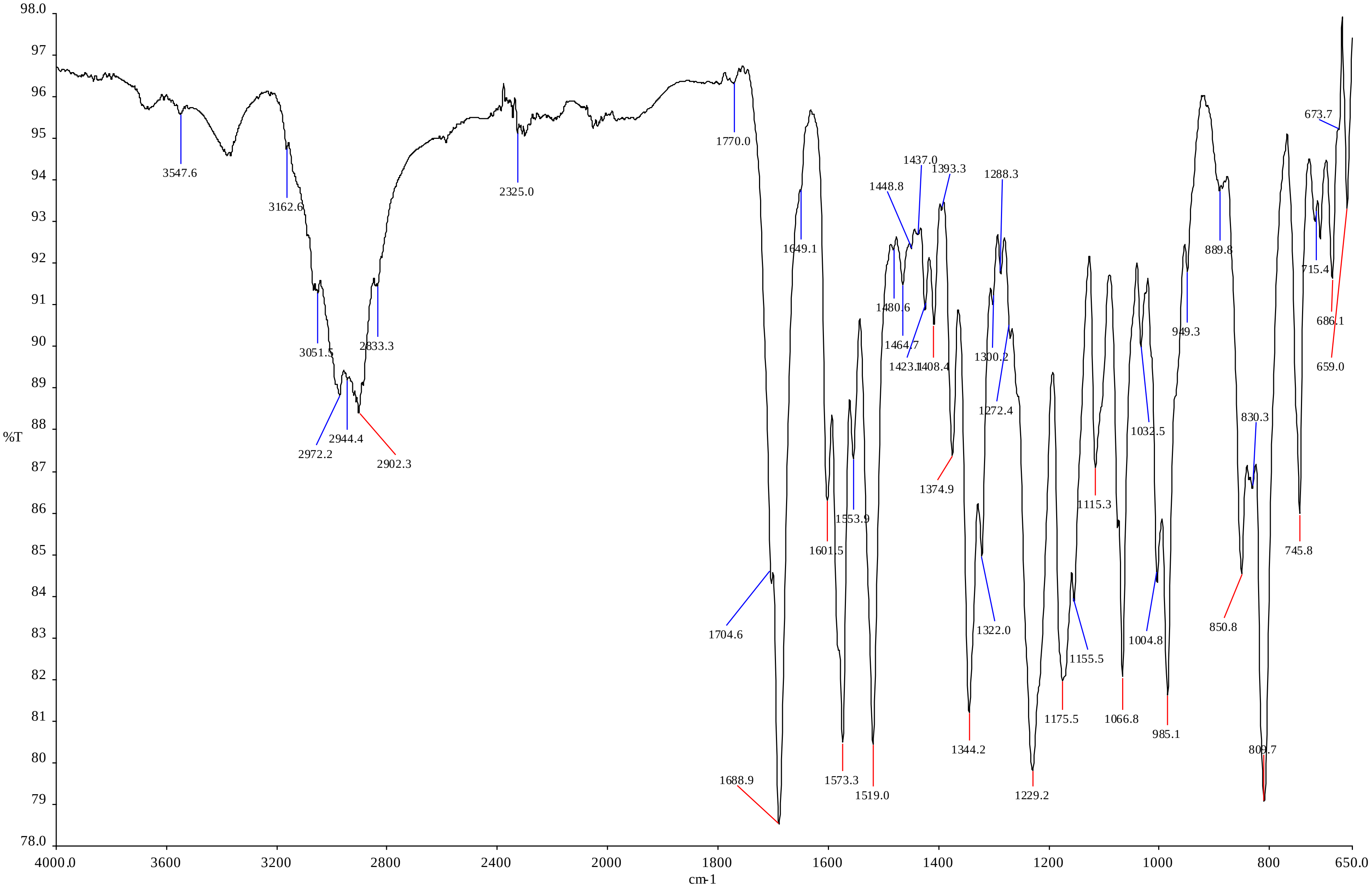

%T
cm-1
3547.6
3162.6
3051.5
2972.2
2944.4
2902.3
2833.3
2325.0
1770.0
1704.6
1688.9
1649.1
1601.5
1573.3
1553.9
1519.0
1480.6
1464.7
1448.8
1437.0
1423.1
1408.4
1393.3
1374.9
1344.2
1322.0
1300.2
1288.3
1272.4
1229.2
1175.5
1155.5
1115.3
1066.8
1032.5
1004.8
985.1
949.3
889.8
850.8
830.3
809.7
745.8
715.4
686.1
673.7
659.0

24

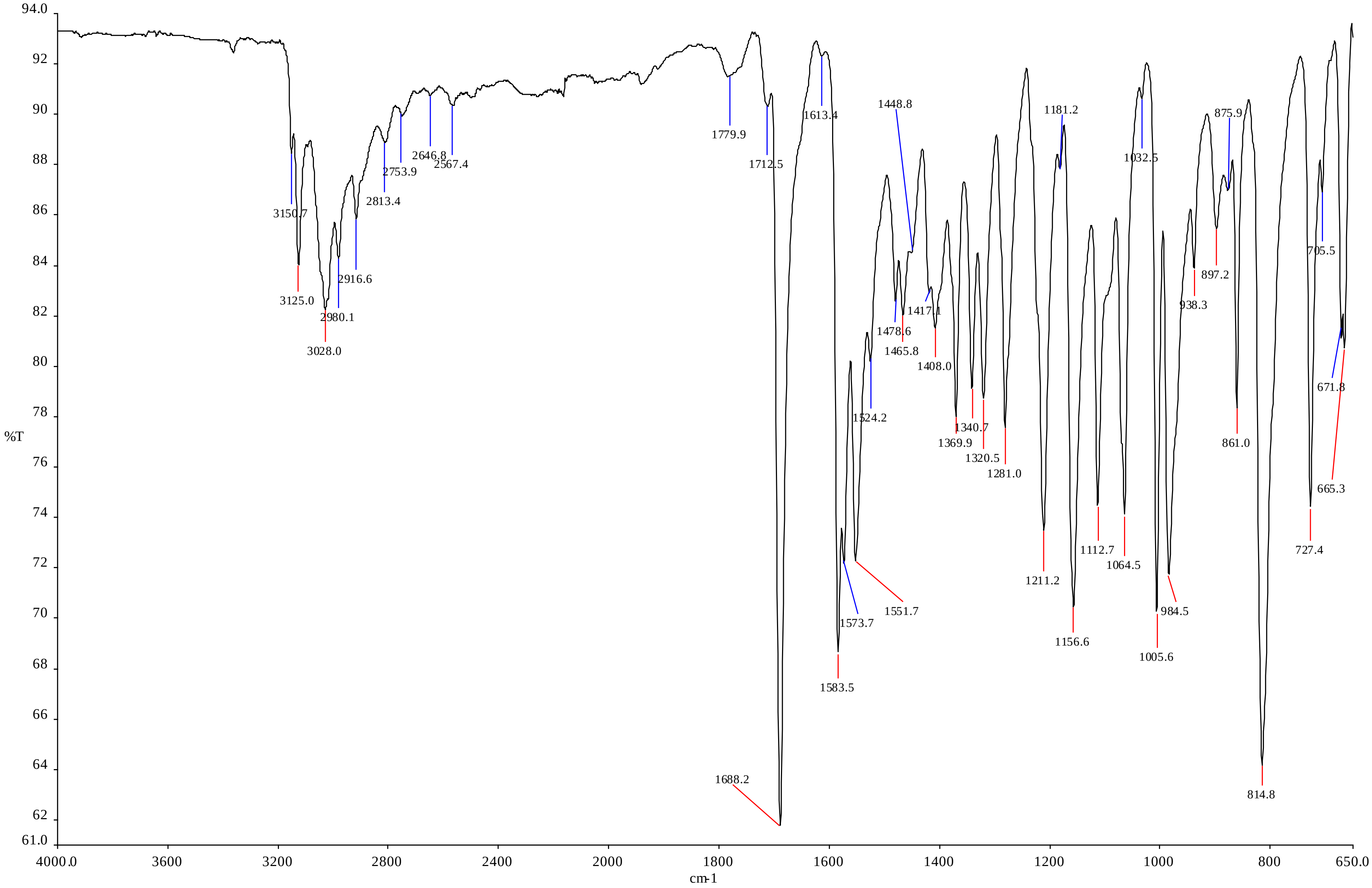
94.0
92
90
88
86
84
82
80
78
76
74
72
70
68
66
64
62
61.0
%T
4000.0
3600
3200
2800
2400
2000
1800
1600
1400
1200
1000
800
650.0
cm-1
3150.7
3125.0
3028.0
2980.1
2916.6
2813.4
2753.9
2646.8
2567.4
1779.9
1712.5
1688.2
1613.4
1583.5
1573.7
1551.7
1524.2
1478.6
1465.8
1448.8
1417.1
1408.0
1369.9
1340.7
1320.5
1281.0
1211.2
1181.2
1156.6
1112.7
1064.5
1032.5
1005.6
984.5
938.3
897.2
875.9
861.0
814.8
727.4
705.5
671.8
665.3

23

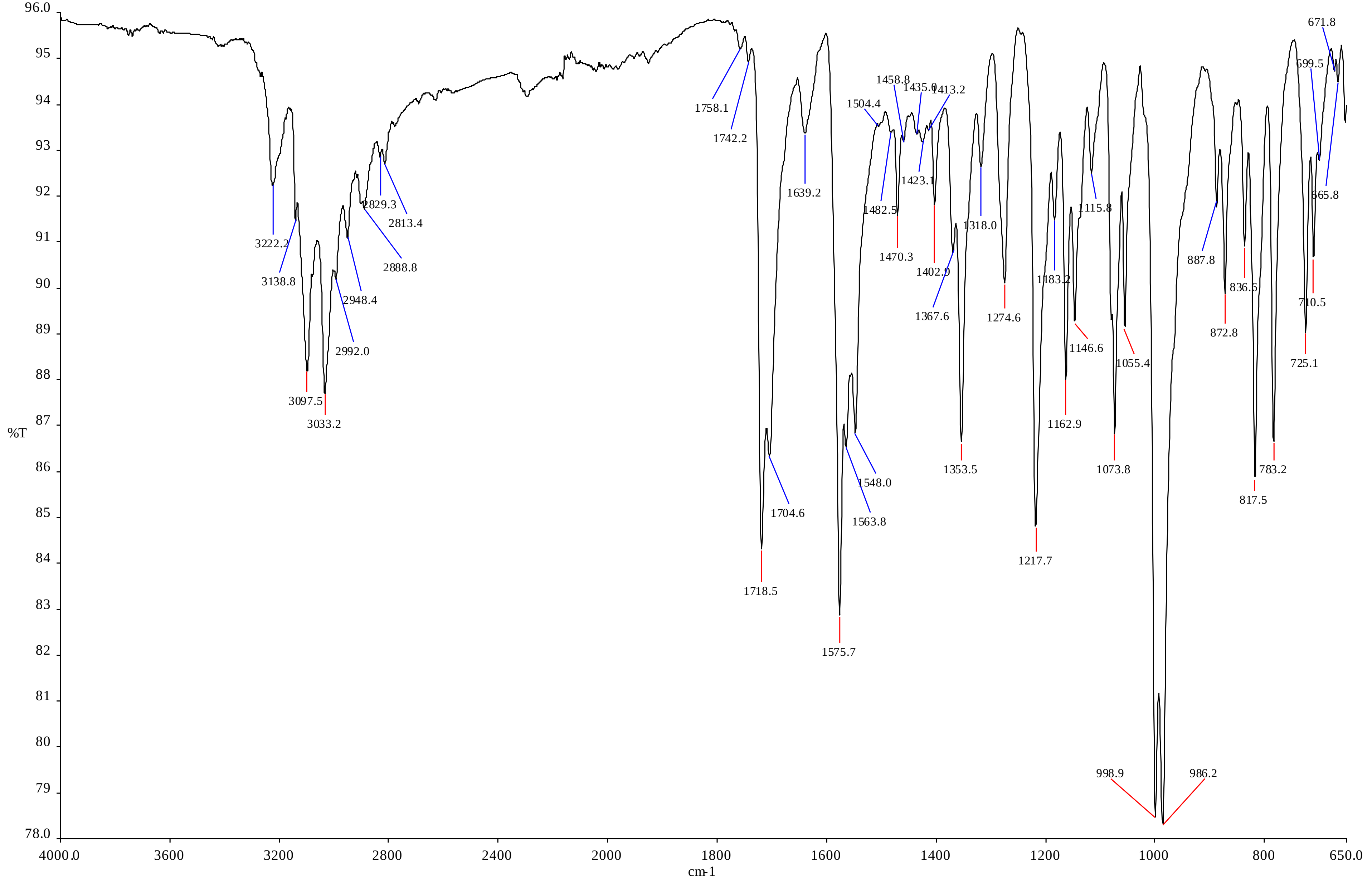

96.0
95
94
93
92
91
90
89
88
87
86
85
84
83
82
81
80
79
78.0
%T
4000.0
3600
3200
2800
2400
2000
1800
1600
1400
1200
1000
800
650.0
cm-1
3222.2
3138.8
3097.5
3033.2
2992.0
2948.4
2888.8
2829.3
2813.4
1758.1
1742.2
1718.5
1704.6
1639.2
1575.7
1563.8
1548.0
1504.4
1482.5
1470.3
1458.8
1435.0
1423.1
1413.2
1402.9
1367.6
1353.5
1318.0
1274.6
1217.7
1183.2
1162.9
1146.6
1115.8
1073.8
1055.4
998.9
986.2
887.8
872.8
836.6
817.5
783.2
725.1
710.5
699.5
671.8
665.8

8

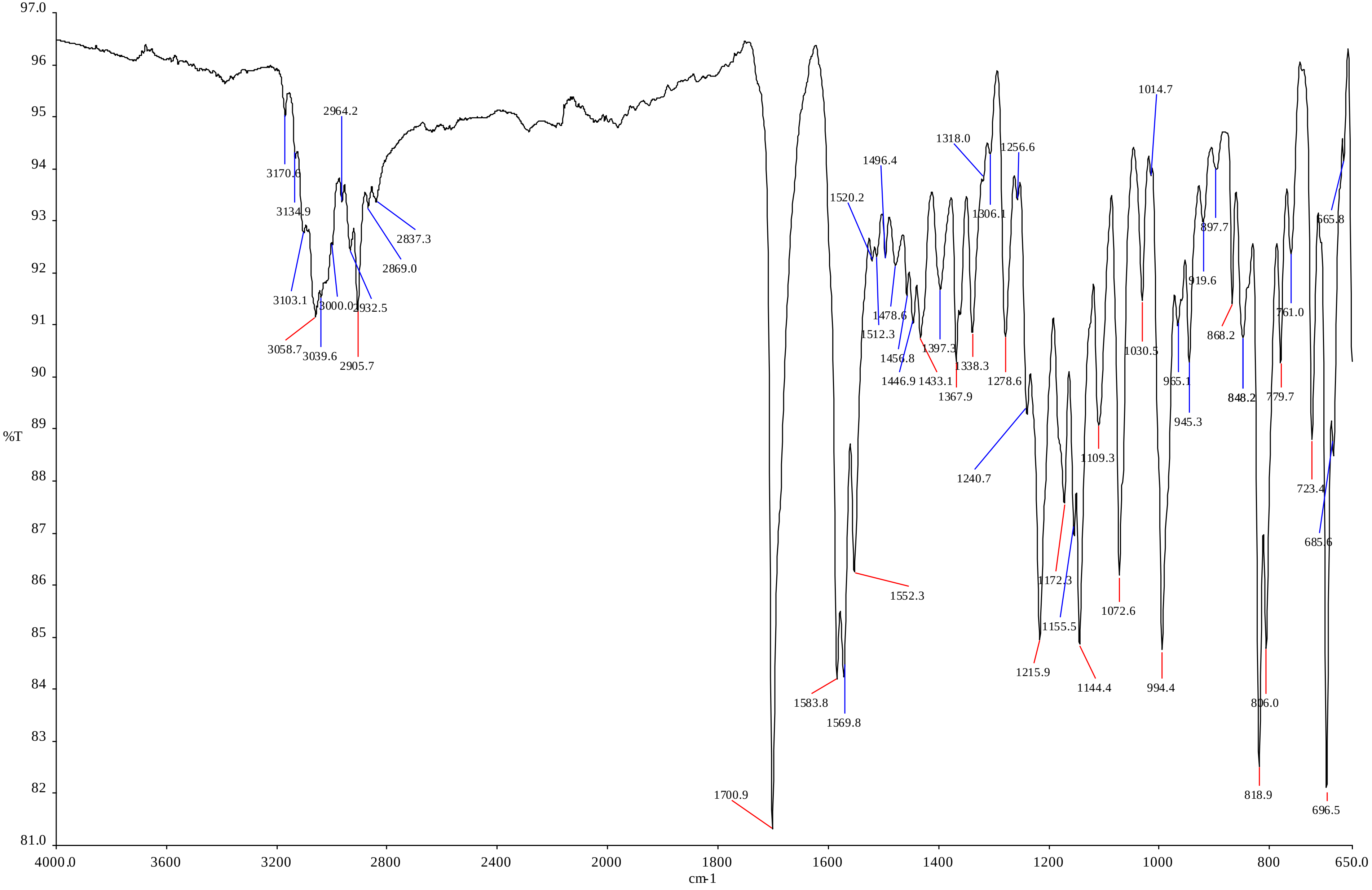
%T
cm-1
97.0
81.0
4000.0
650.0
1700.9
1583.8
1569.8
1552.3
1520.2
1512.3
1496.4
1478.6
1456.8
1446.9
1433.1
1397.3
1367.9
1338.3
1318.0
1306.1
1278.6
1256.6
1240.7
1215.9
1172.3
1155.5
1144.4
1109.3
1072.6
1030.5
1014.7
994.4
965.1
945.3
919.6
897.7
868.2
848.2
818.9
806.0
779.7
761.0
723.4
696.5
685.6
665.8
3170.6
3134.9
3103.1
3058.7
3039.6
3000.0
2964.2
2932.5
2905.7
2869.0
2837.3

25

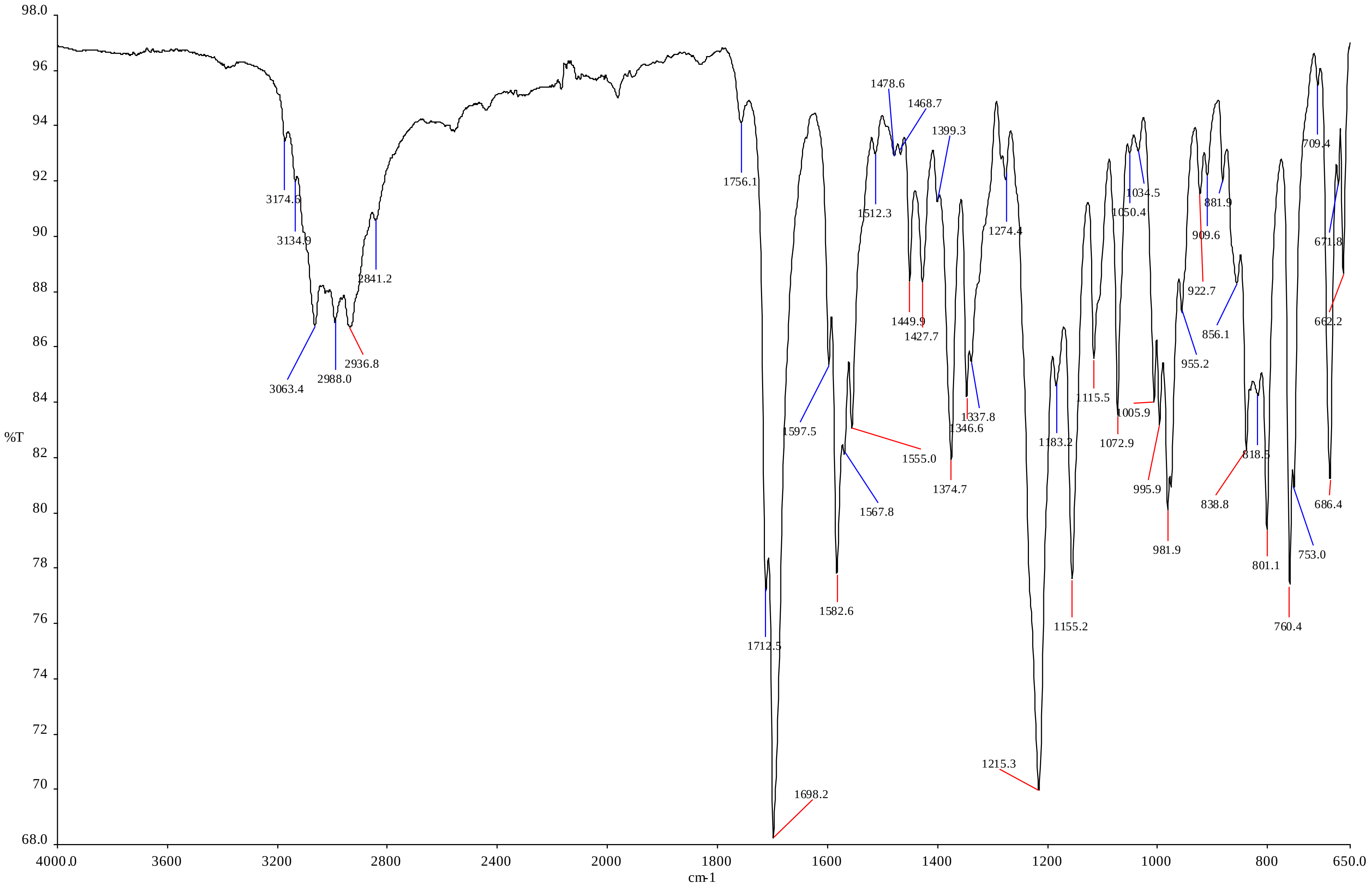
%T
cm-1
3174.6
3134.9
3063.4
2988.0
2936.8
2841.2
1756.1
1712.5
1698.2
1597.5
1582.6
1567.8
1555.0
1512.3
1478.6
1468.7
1449.9
1427.7
1399.3
1374.7
1346.6
1337.8
1274.4
1215.3
1183.2
1155.2
1115.5
1072.9
1050.4
1034.5
1005.9
995.9
981.9
955.2
922.7
909.6
881.9
856.1
838.8
818.5
801.1
760.4
753.0
709.4
686.4
671.8
662.2

26

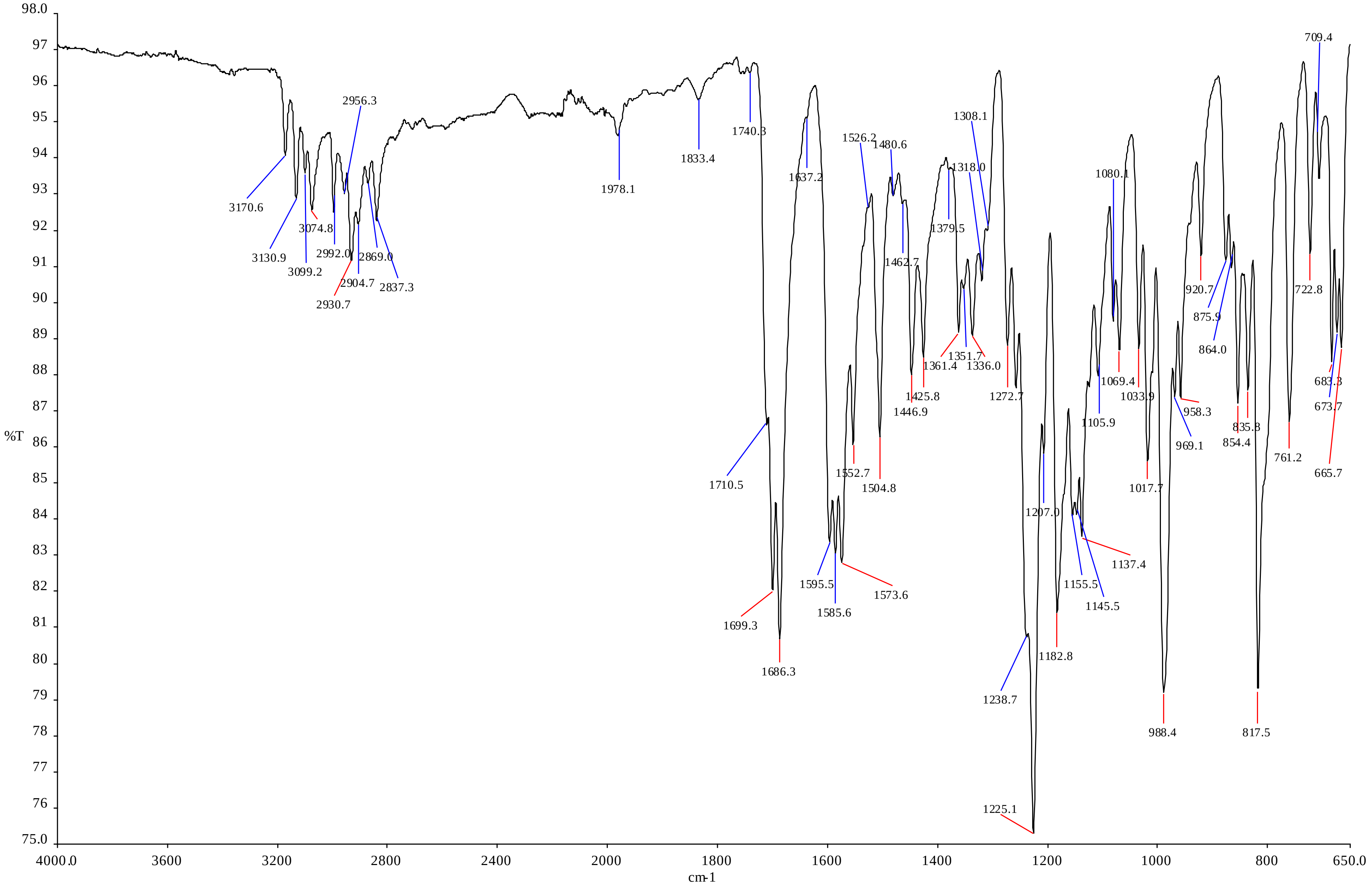

%T
cm-1
98.0
75.0
4000.0
650.0
3170.6
3130.9
3099.2
3074.8
2992.0
2956.3
2930.7
2904.7
2869.0
2837.3
1978.1
1833.4
1740.3
1710.5
1699.3
1686.3
1637.2
1595.5
1585.6
1573.6
1552.7
1526.2
1504.8
1480.6
1462.7
1446.9
1425.8
1379.5
1361.4
1351.7
1336.0
1318.0
1308.1
1272.7
1238.7
1225.1
1207.0
1182.8
1155.5
1145.5
1137.4
1105.9
1080.1
1069.4
1033.9
1017.7
988.4
969.1
958.3
920.7
875.9
864.0
854.4
835.8
817.5
761.2
722.8
709.4
683.3
673.7
665.7

16

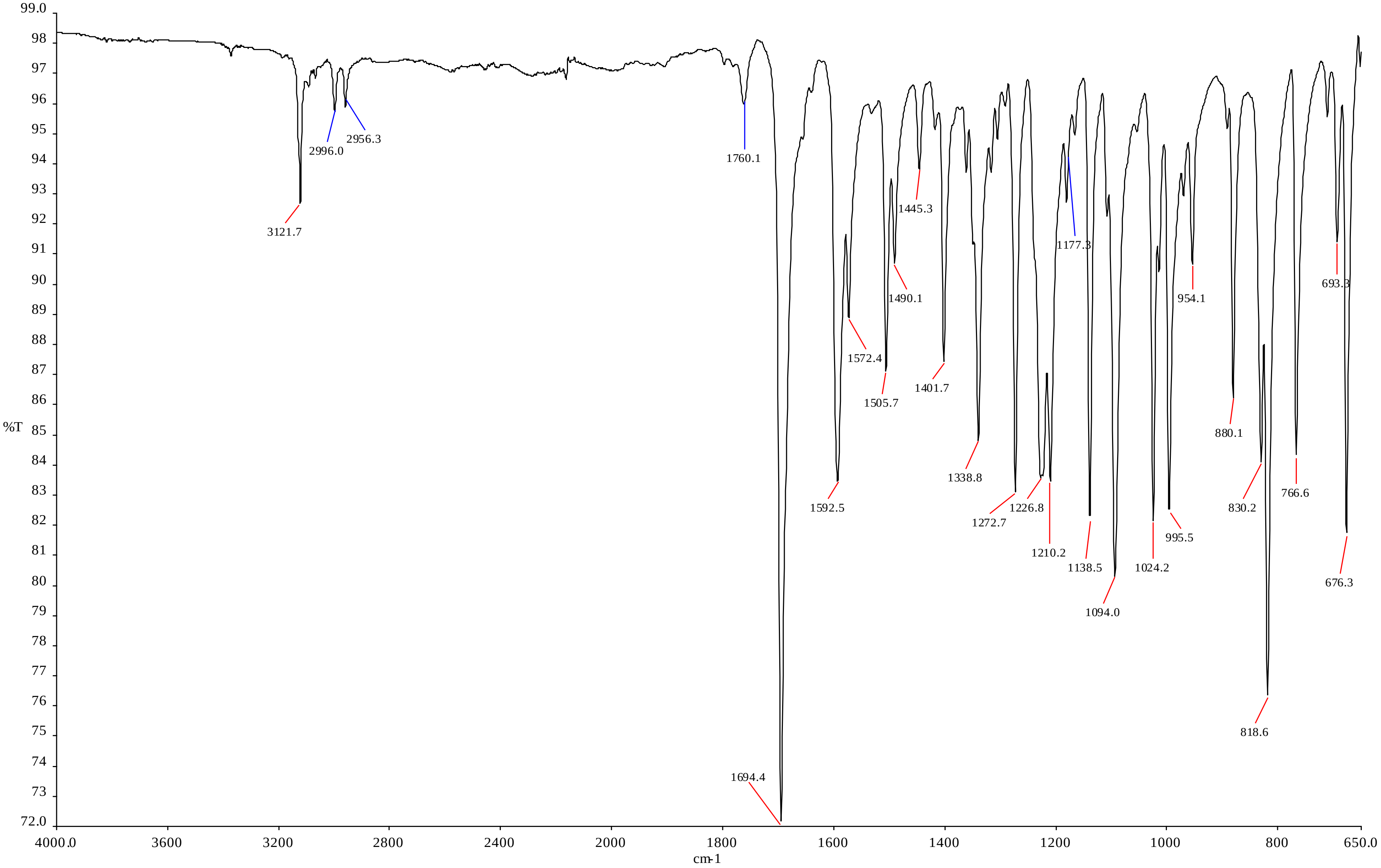

%T
cm-1
4000.0
3600
3200
2800
2400
2000
1800
1600
1400
1200
1000
800
650.0
99.0
72.0
3121.7
2996.0
2956.3
1760.1
1694.4
1592.5
1572.4
1505.7
1490.1
1445.3
1401.7
1338.8
1272.7
1226.8
1210.2
1177.3
1138.5
1094.0
1024.2
995.5
954.1
880.1
830.2
818.6
766.6
693.3
676.3

15

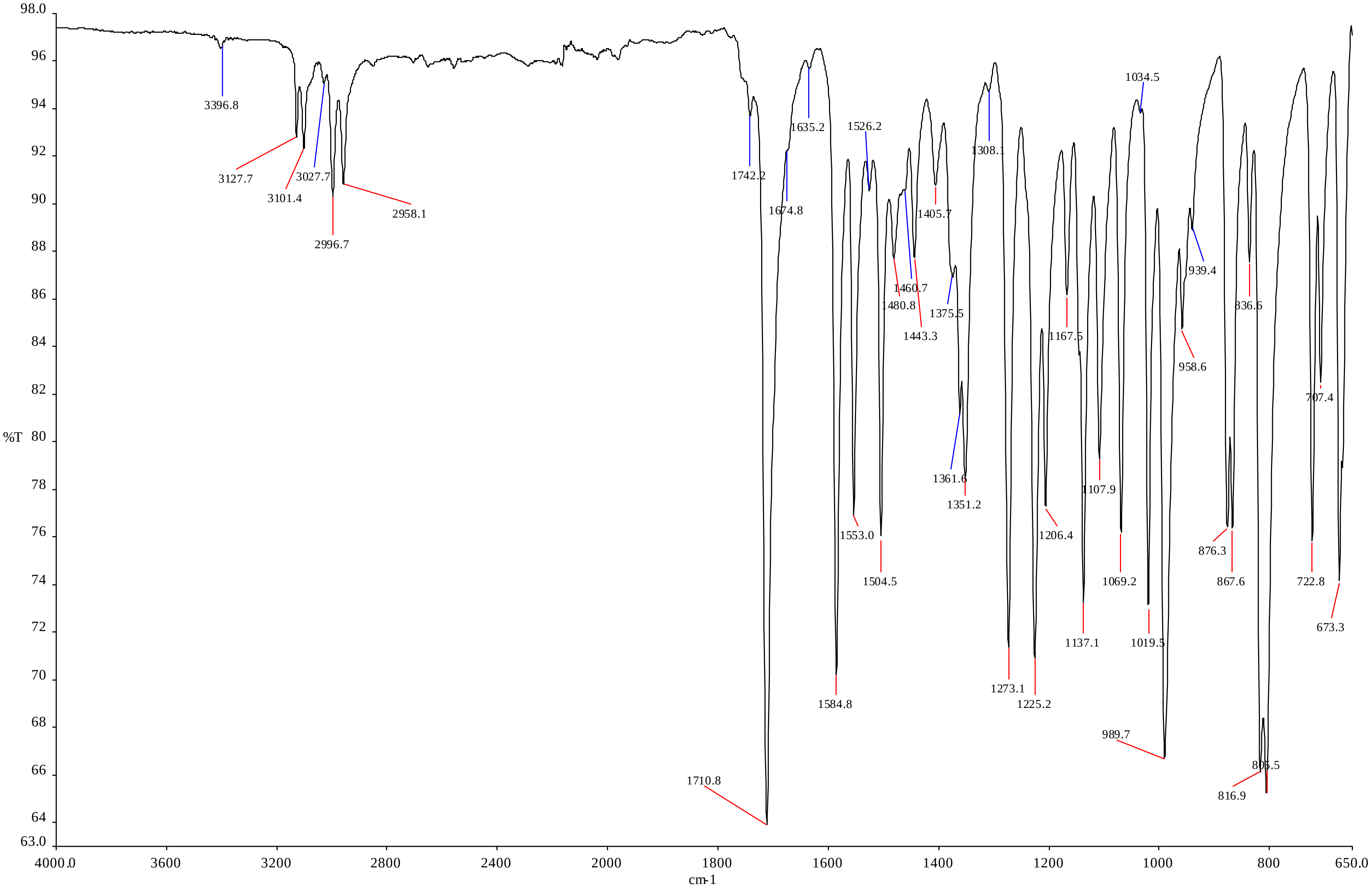
98.0
96
94
92
90
88
86
84
82
%T 80
78
76
74
72
70
68
66
64
63.0
4000.0
3600
3200
2800
2400
2000
1800
1600
1400
1200
1000
800
650.0
cm-1
3396.8
3127.7
3101.4
3027.7
2996.7
2958.1
1742.2
1710.8
1674.8
1635.2
1584.8
1553.0
1526.2
1504.5
1480.8
1460.7
1443.3
1405.7
1375.5
1361.6
1351.2
1308.1
1273.1
1225.2
1206.4
1167.5
1137.1
1107.9
1069.2
1034.5
1019.5
989.7
958.6
939.4
876.3
867.6
836.6
816.9
805.5
722.8
707.4
673.3

14

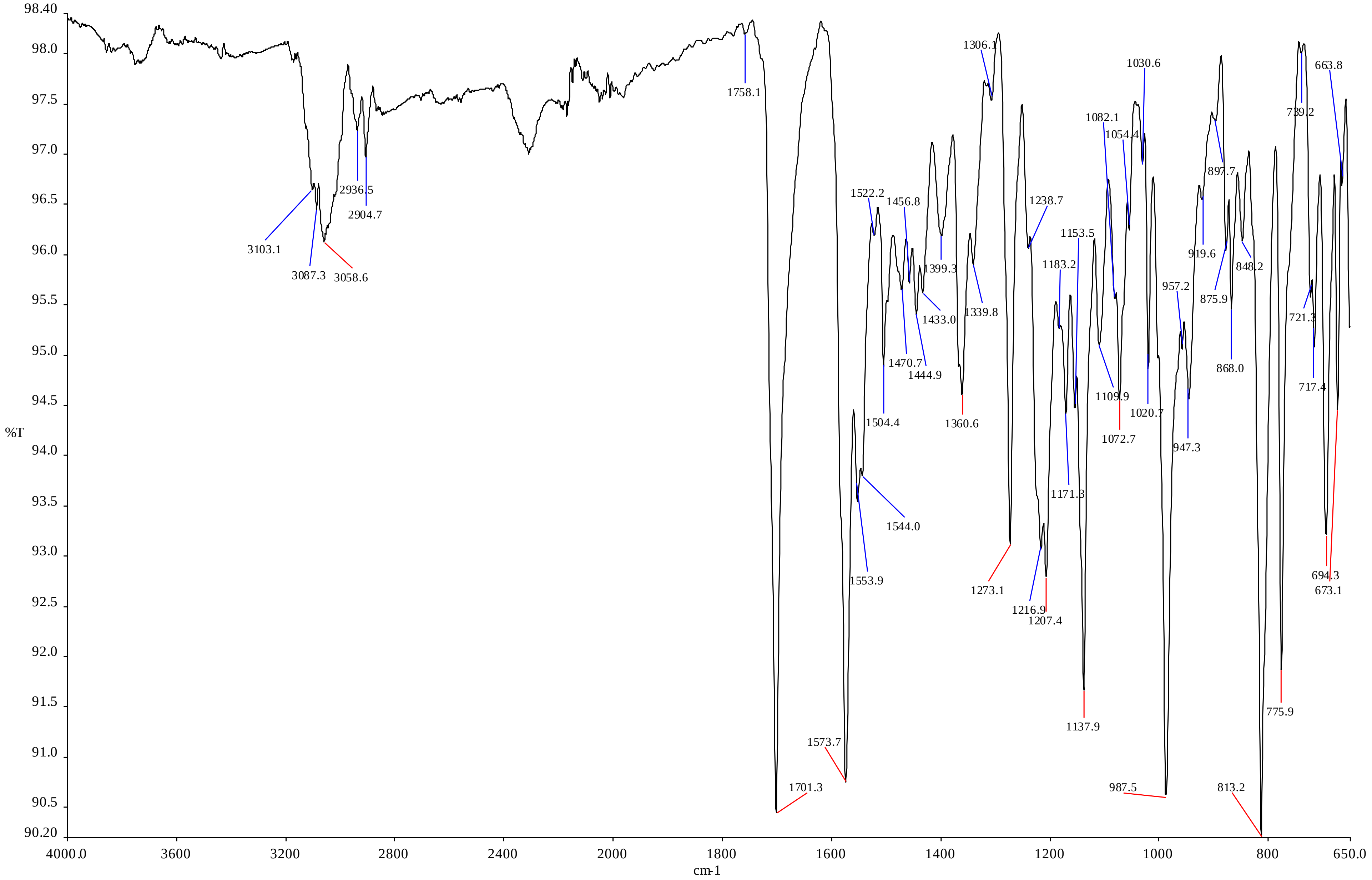
%T
cm-1
98.40
98.0
97.5
97.0
96.5
96.0
95.5
95.0
94.5
94.0
93.5
93.0
92.5
92.0
91.5
91.0
90.5
90.20
4000.0
3600
3200
2800
2400
2000
1800
1600
1400
1200
1000
800
650.0
3103.1
3087.3
3058.6
2936.5
2904.7
1758.1
1701.3
1573.7
1553.9
1544.0
1522.2
1504.4
1470.7
1456.8
1444.9
1433.0
1399.3
1360.6
1339.8
1306.1
1273.1
1238.7
1216.9
1207.4
1183.2
1171.3
1153.5
1137.9
1109.9
1082.1
1072.7
1054.4
1030.6
1020.7
987.5
957.2
947.3
919.6
897.7
875.9
868.0
848.2
813.2
775.9
739.2
721.3
717.4
694.3
673.1
663.8

13

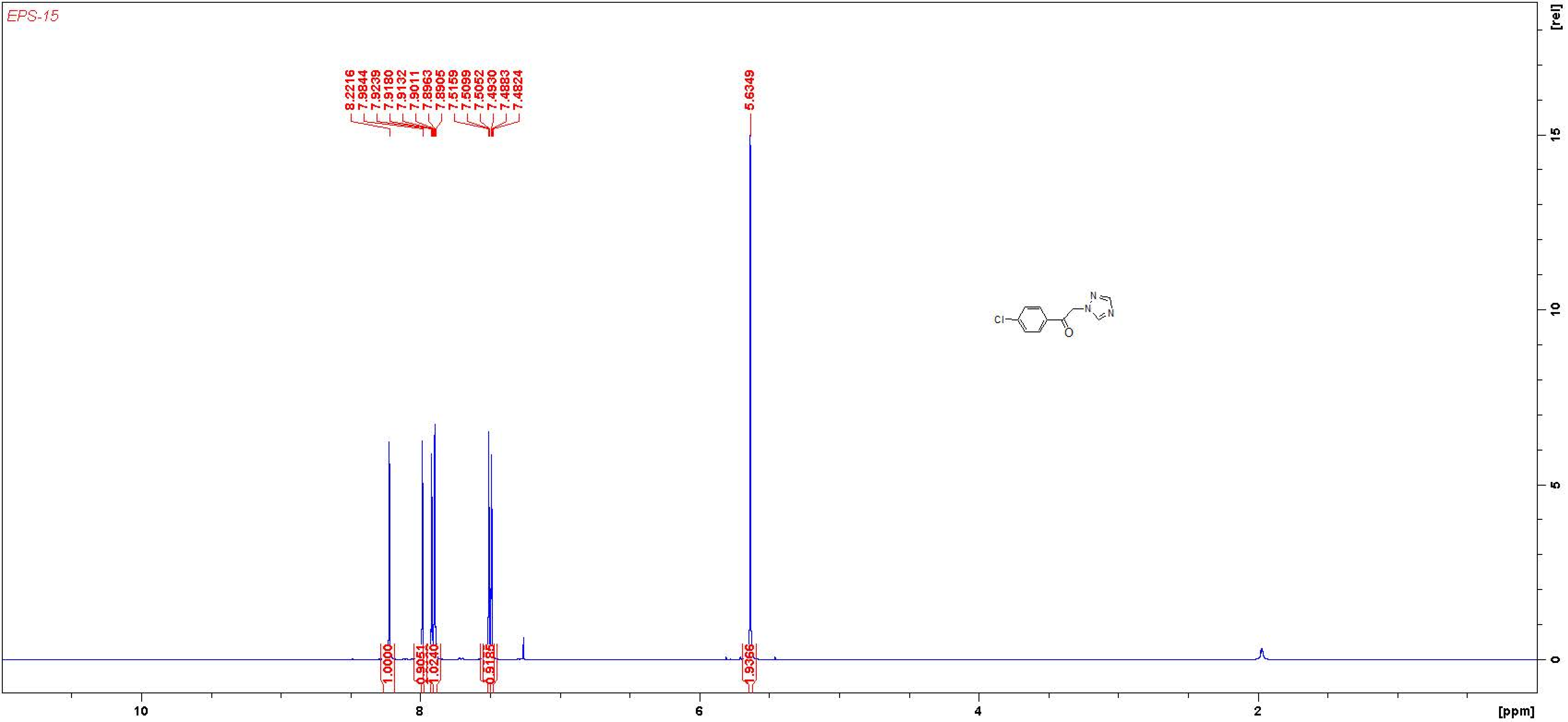
EPS-15
8.2216
7.9844
7.9239
7.9180
7.9132
7.9011
7.8963
7.8905
7.5159
7.5099
7.5052
7.4930
7.4883
7.4824
5.6349
1.0000
0.9051
1.0240
0.9185
1.9366
Cl
N
N
N
O
[rel]
15
10
5
0
10
8
6
4
2
[ppm]

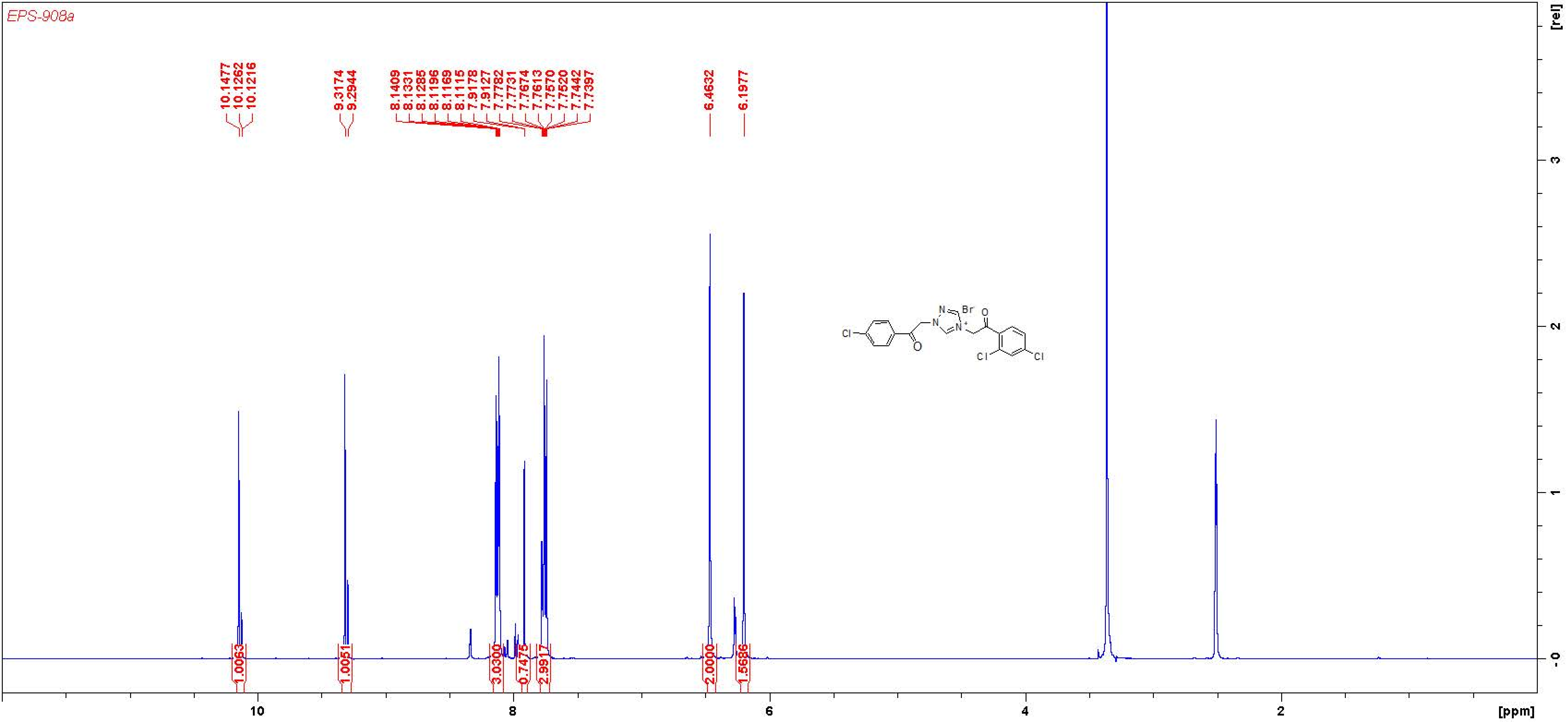
EPS-908a
10.1477
10.1262
10.1216
9.3174
9.2944
8.1409
8.1331
8.1285
8.1196
8.1169
8.1115
7.9178
7.9127
7.7782
7.7731
7.7674
7.7613
7.7570
7.7520
7.7442
7.7397
6.4632
6.1977
1.0063
1.0051
3.0300
0.7475
2.9917
2.0000
1.5686
Br
O
N
Cl
Cl
Cl
[rel]
[ppm]

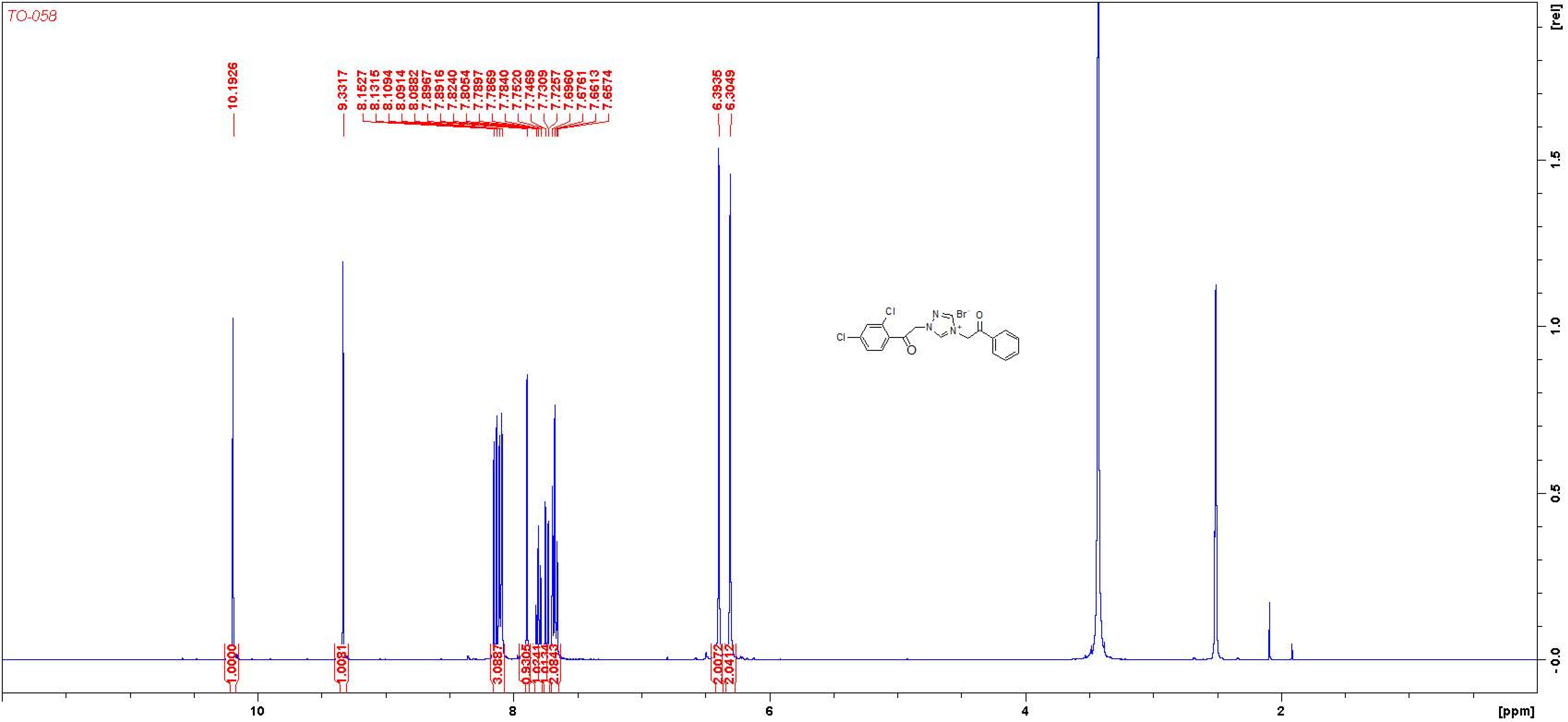

TO-058
10.1926
9.3317
8.1527
8.1315
8.1094
8.0914
8.0882
7.8967
7.8916
7.8240
7.8054
7.7897
7.7869
7.7840
7.7520
7.7469
7.7309
7.7257
7.6960
7.6761
7.6613
7.6574
6.3935
6.3049
1.0000
1.0081
3.0887
0.9305
1.0244
1.0144
2.0843
2.0072
2.0412
Cl
Cl
N
N
Br
N+
O
O
[rel]
[ppm]

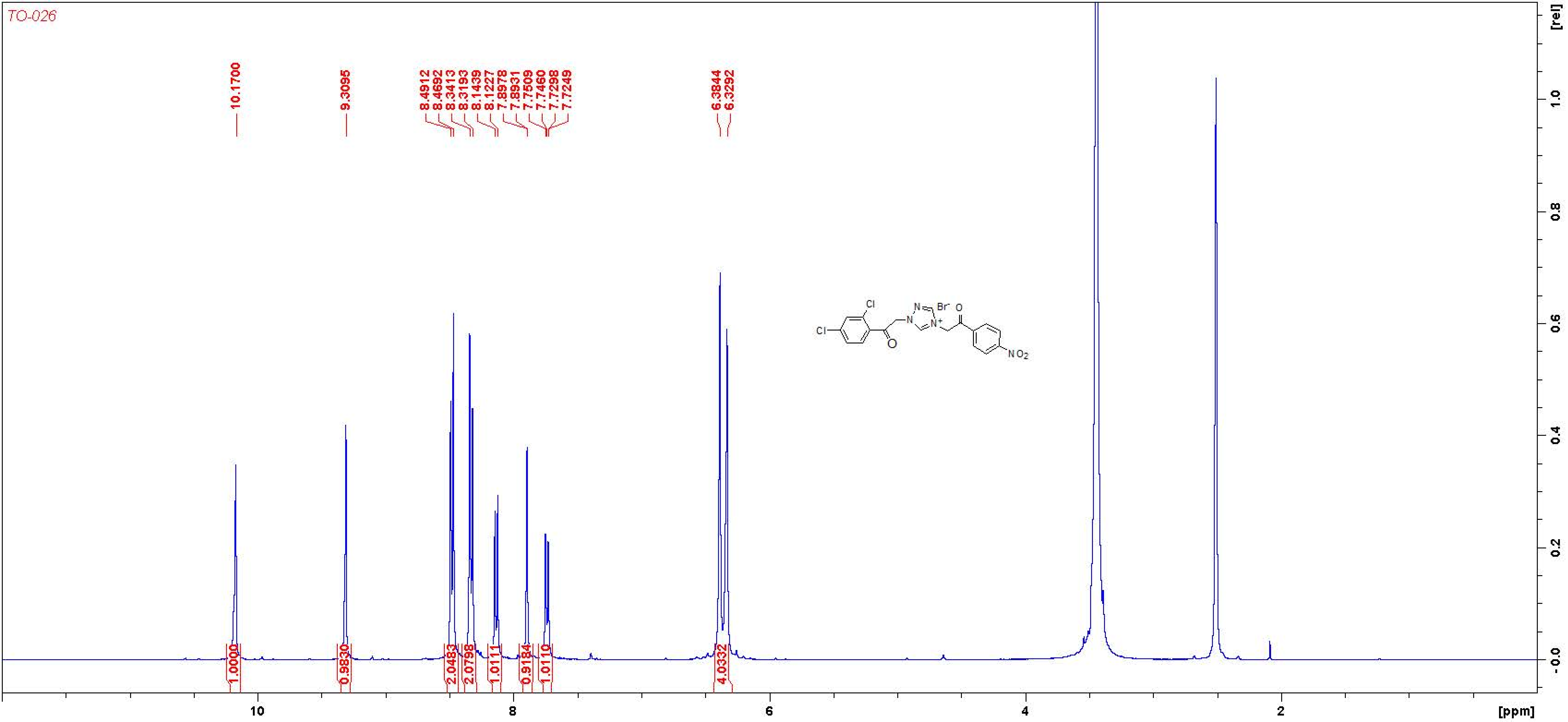
TO-026
10.1700
9.3095
8.4912
8.4692
8.3413
8.3193
8.1439
8.1227
7.8978
7.8931
7.7509
7.7460
7.7298
7.7249
6.3844
6.3292
1.0000
0.9830
2.0483
2.0798
1.0111
0.9184
1.0110
4.0332
10
8
6
4
2
[ppm]
[rel]
1.0
0.8
0.6
0.4
0.2
-0.0
Cl
Cl
Br⁻
N
N
N⁺
O
O
NO2

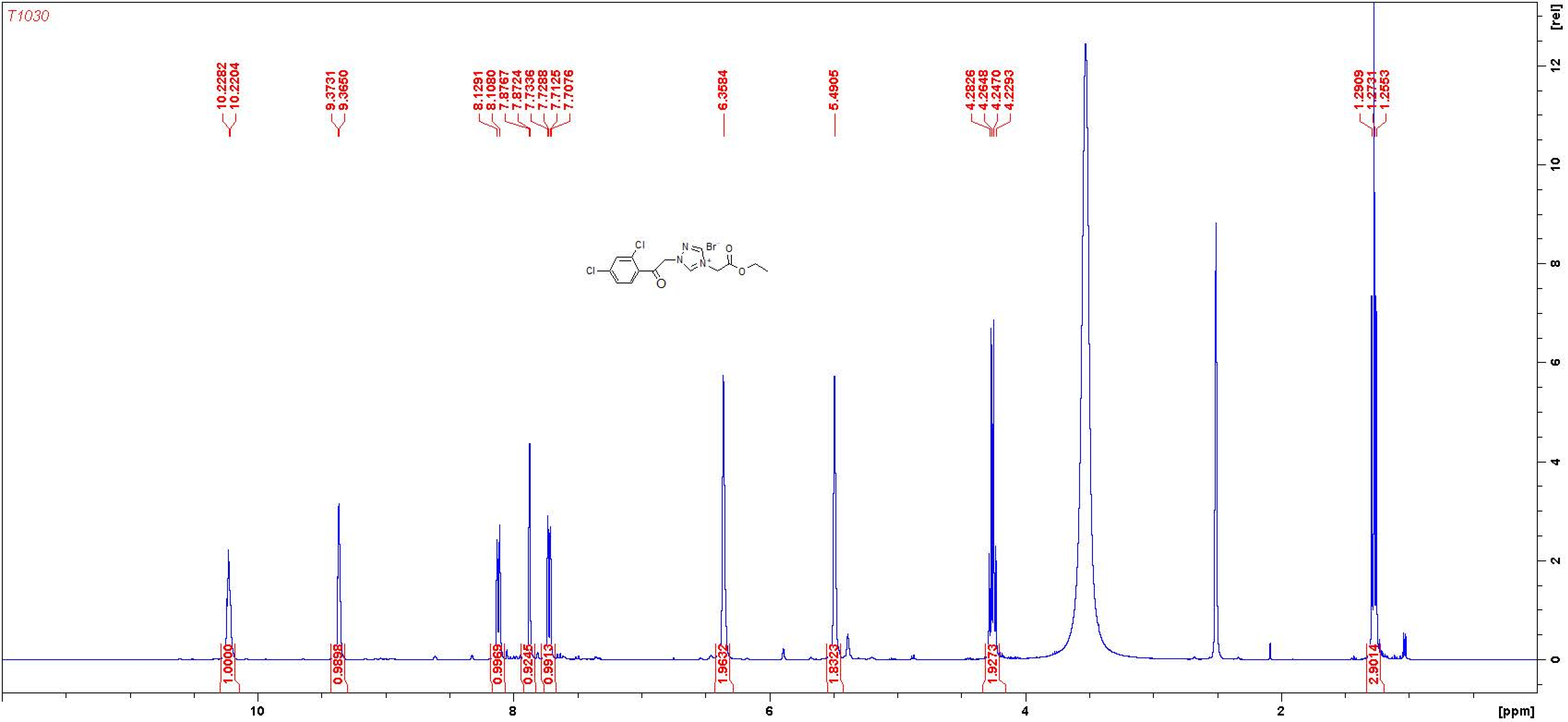
T1030
10.2282
10.2204
9.3731
9.3650
8.1291
8.1080
7.8767
7.8724
7.7336
7.7288
7.7125
7.7076
6.3584
5.4905
4.2826
4.2648
4.2470
4.2293
1.2909
1.2731
1.2553
1.0000
0.9898
0.9969
0.9245
0.9913
1.9632
1.8323
1.9273
2.9014
[rel]
[ppm]

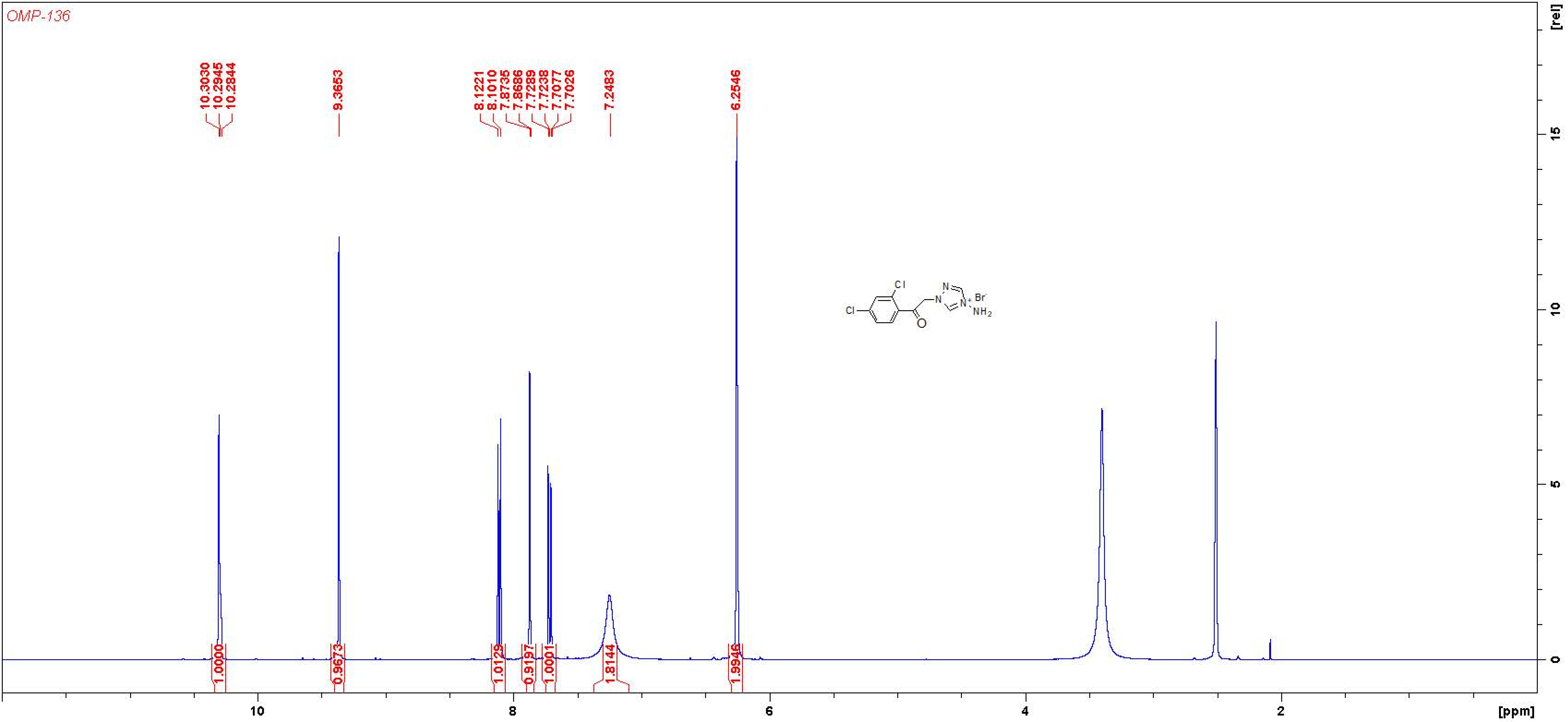
OMP-136
10.3030
10.2945
10.2844
9.3653
8.1221
8.1010
7.8735
7.8686
7.7289
7.7238
7.7077
7.7026
7.2483
6.2546
1.0000
0.9673
1.0129
0.9197
1.0001
1.8144
1.9946
Cl
Cl
N
N
N
Br
NH2
O
[rel]
15
10
5
0
10
8
6
4
2
[ppm]

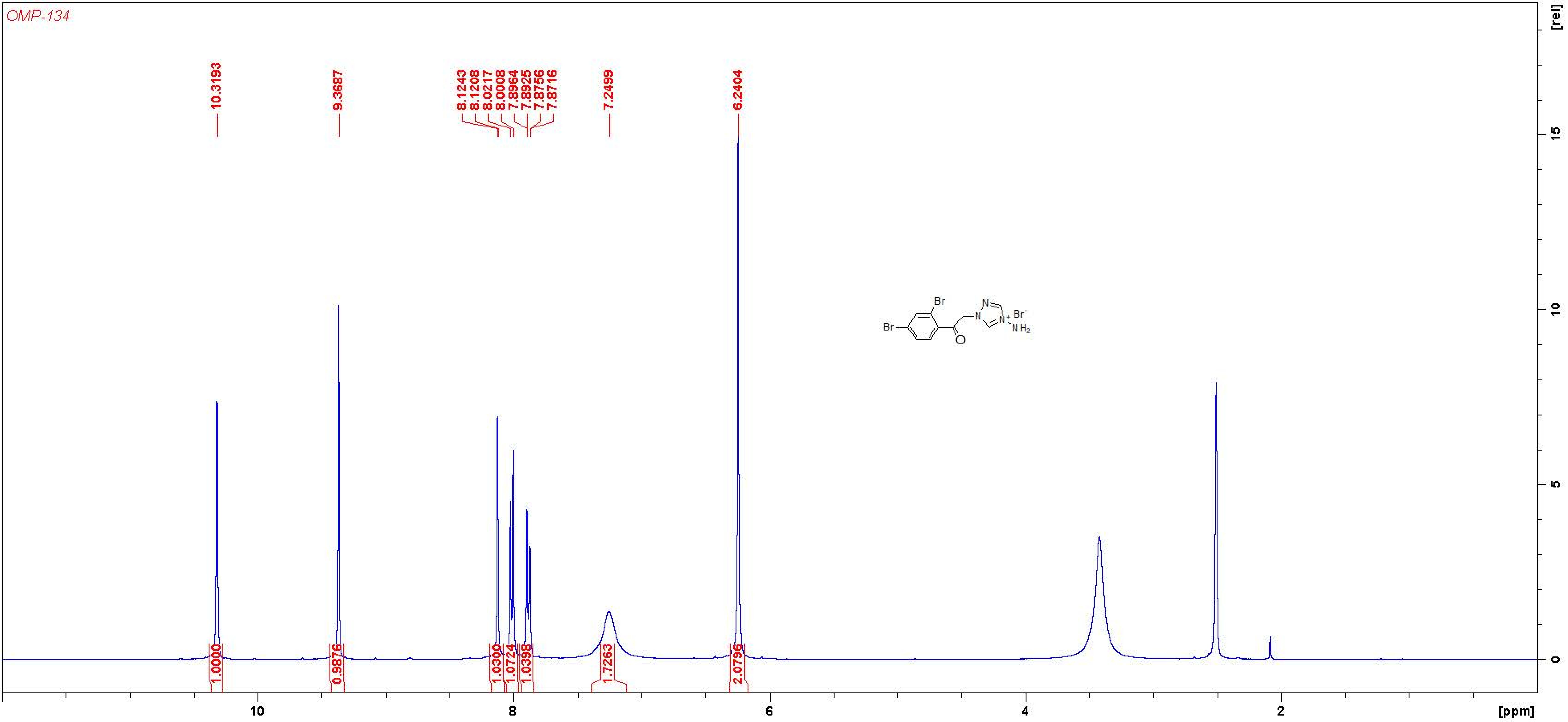
OMP-134
10.3193
9.3687
8.1243
8.1208
8.0217
8.0008
7.8964
7.8925
7.8756
7.8716
7.2499
6.2404
1.0000
0.9876
1.0300
1.0724
1.0398
1.7263
2.0796
Br
Br
N
N
N+
Br-
NH2
O
[rel]
15
10
5
0
10
8
6
4
2
[ppm]

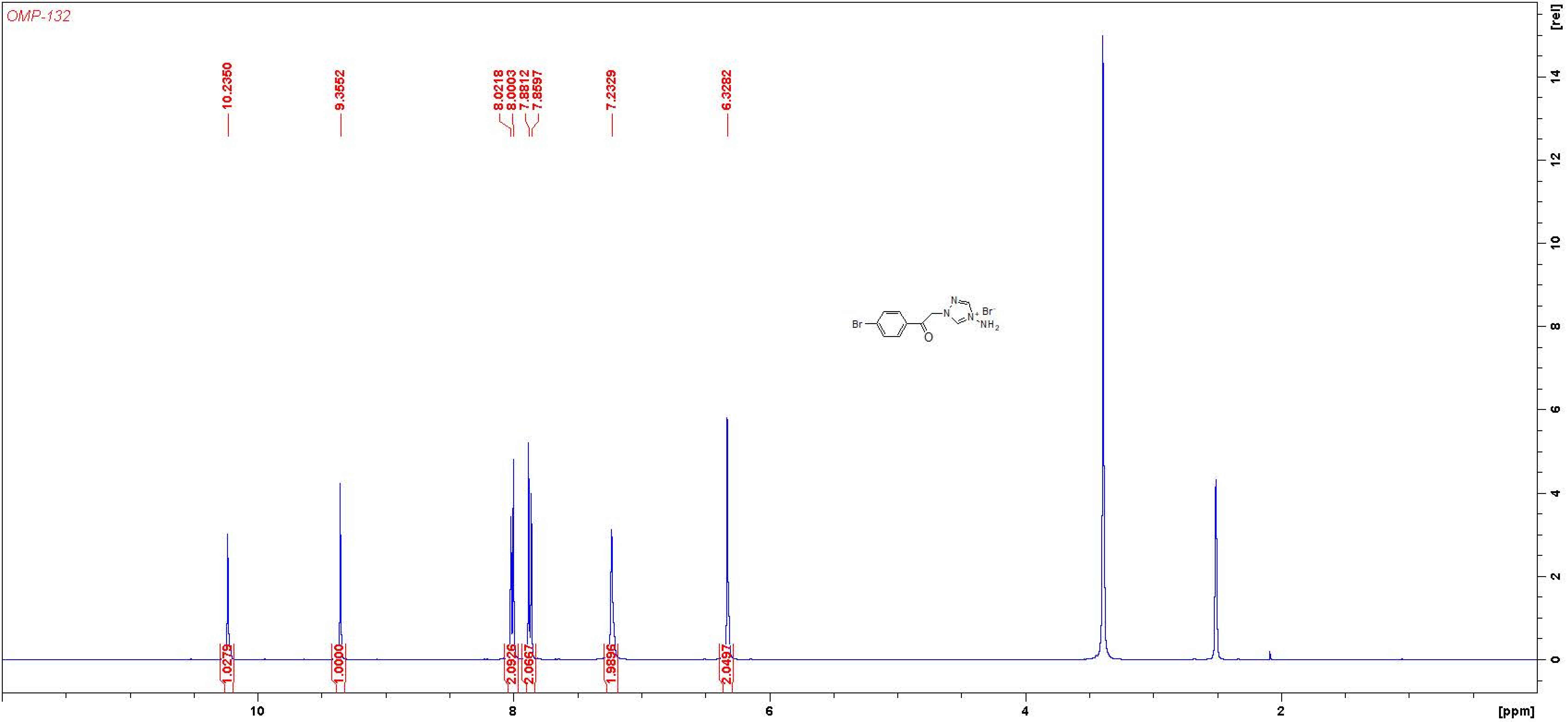

OMP-132
10.2350
9.3552
8.0218
8.0003
7.8812
7.8597
7.2329
6.3282
1.0279
1.0000
2.0926
2.0667
1.9896
2.0497
Br
O
N
N
N+
Br-
NH2
[rel]
14
12
10
8
6
4
2
0
10
8
6
4
2
[ppm]

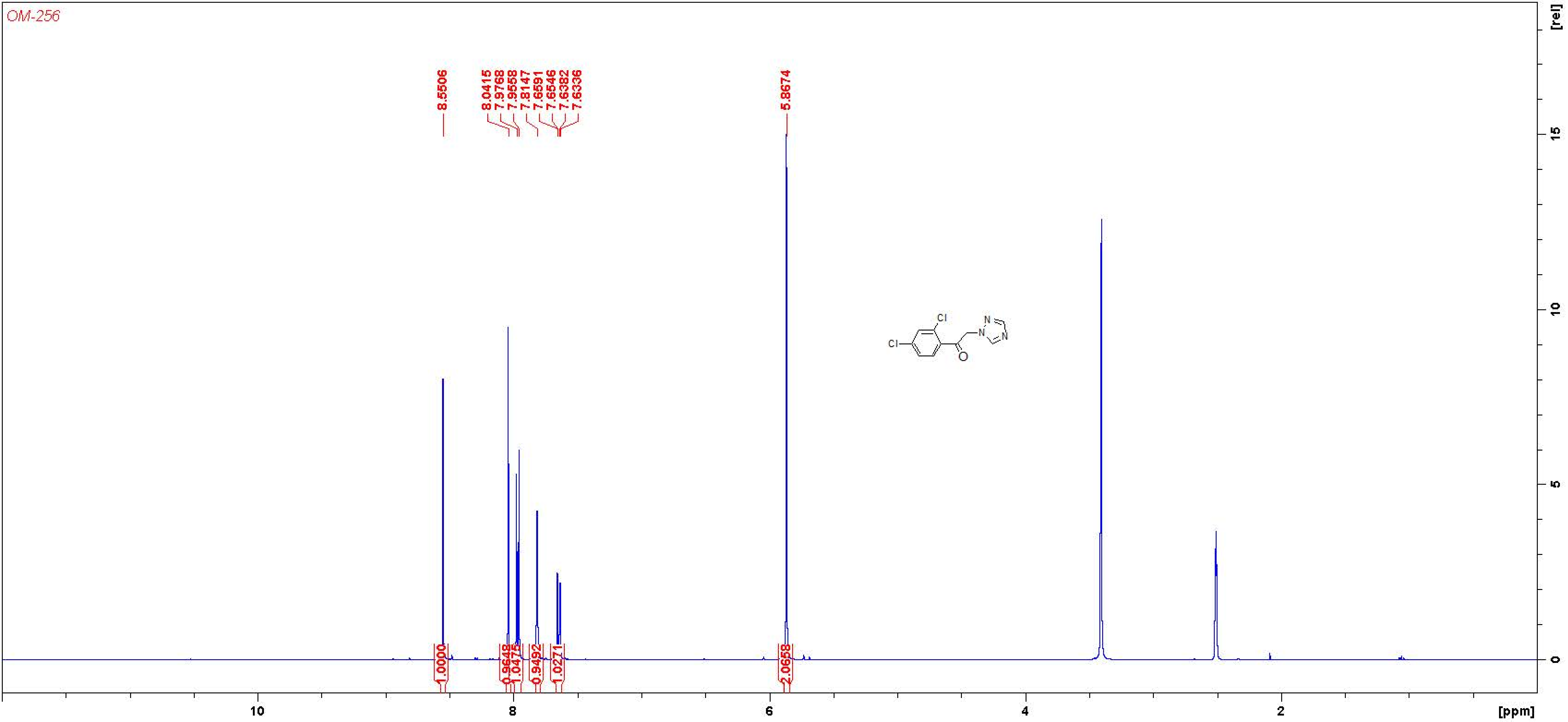
OM-256
8.5506
8.0415
7.9768
7.9558
7.8147
7.6591
7.6546
7.6382
7.6336
5.8674
1.0000
0.9648
1.0475
0.9492
1.0271
2.0658
Cl
Cl
N
N
N
O
[rel]
15
10
5
0
10
8
6
4
2
[ppm]

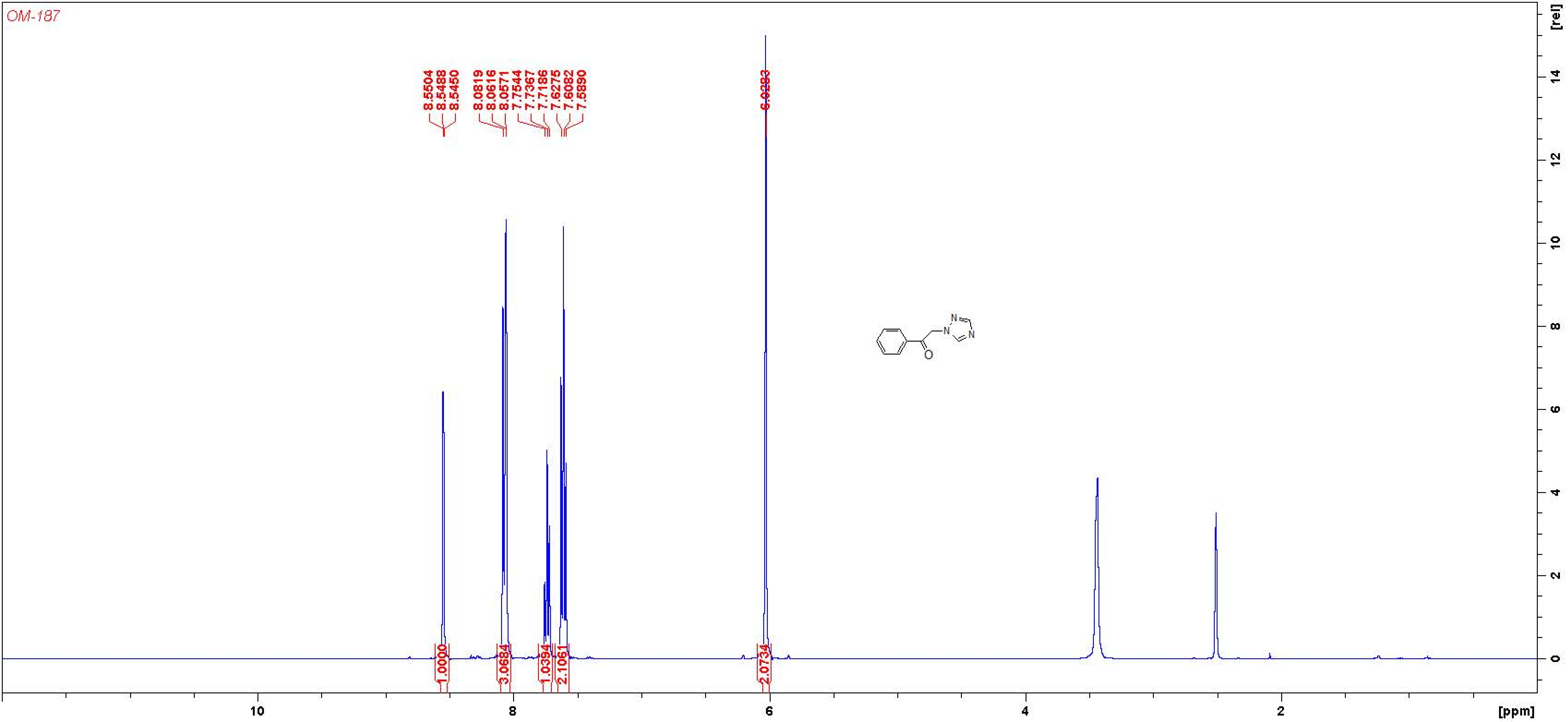

OM-187
8.5504
8.5488
8.5450
8.0819
8.0616
8.0571
7.7544
7.7367
7.7186
7.6275
7.6082
7.5890
6.0283
1.0000
3.0684
1.0394
2.1061
2.0734
[rel]
[ppm]
O
N
N
N

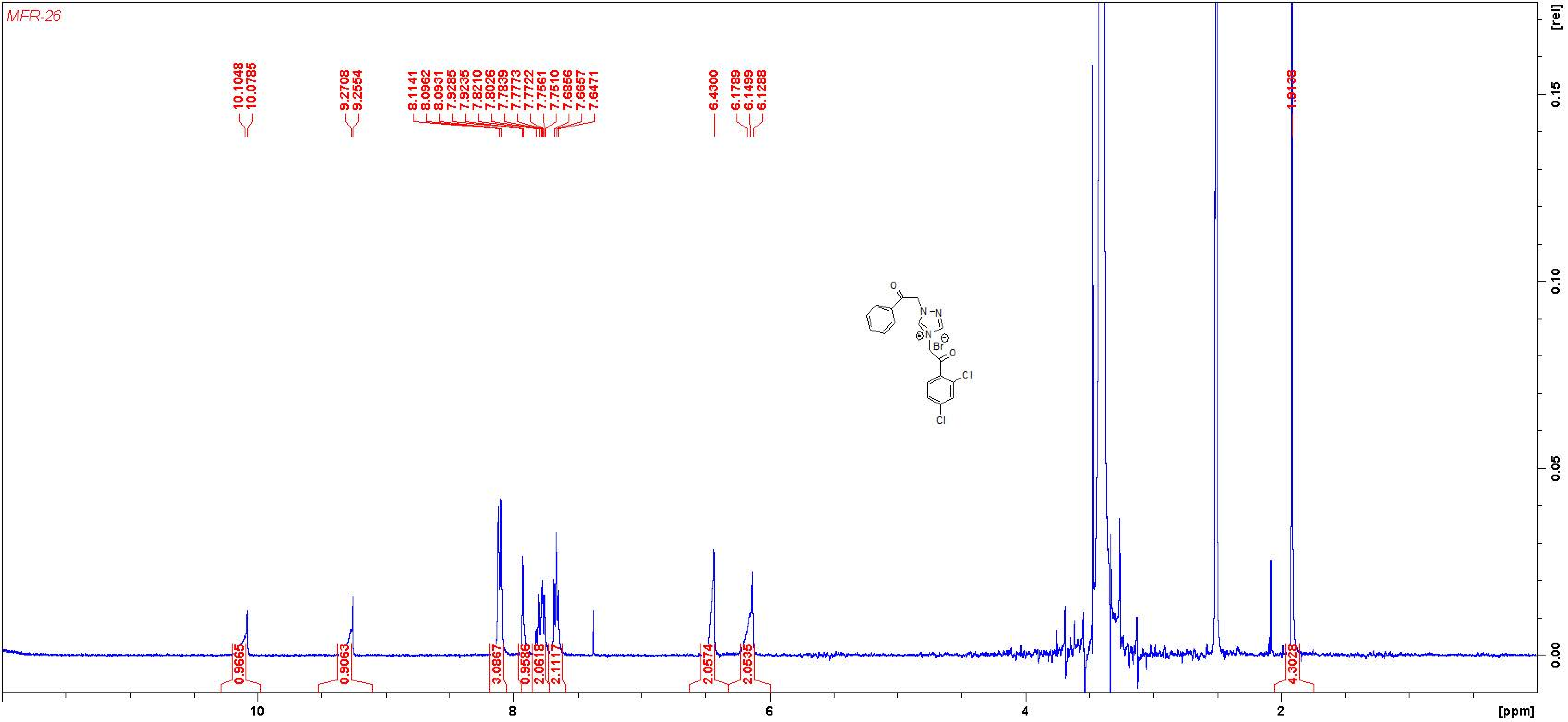
MFR-26
10.1048
10.0785
9.2708
9.2554
8.1141
8.0962
8.0931
7.9285
7.9235
7.8210
7.8026
7.7839
7.7773
7.7722
7.7561
7.7510
7.6856
7.6657
7.6471
6.4300
6.1789
6.1499
6.1288
1.9138
0.9665
0.9063
3.0867
0.9586
2.0618
2.1117
2.0574
2.0535
4.3028
O
N
N
N
Br
O
Cl
Cl
[rel]
0.15
0.10
0.05
0.00
10
8
6
4
2
[ppm]

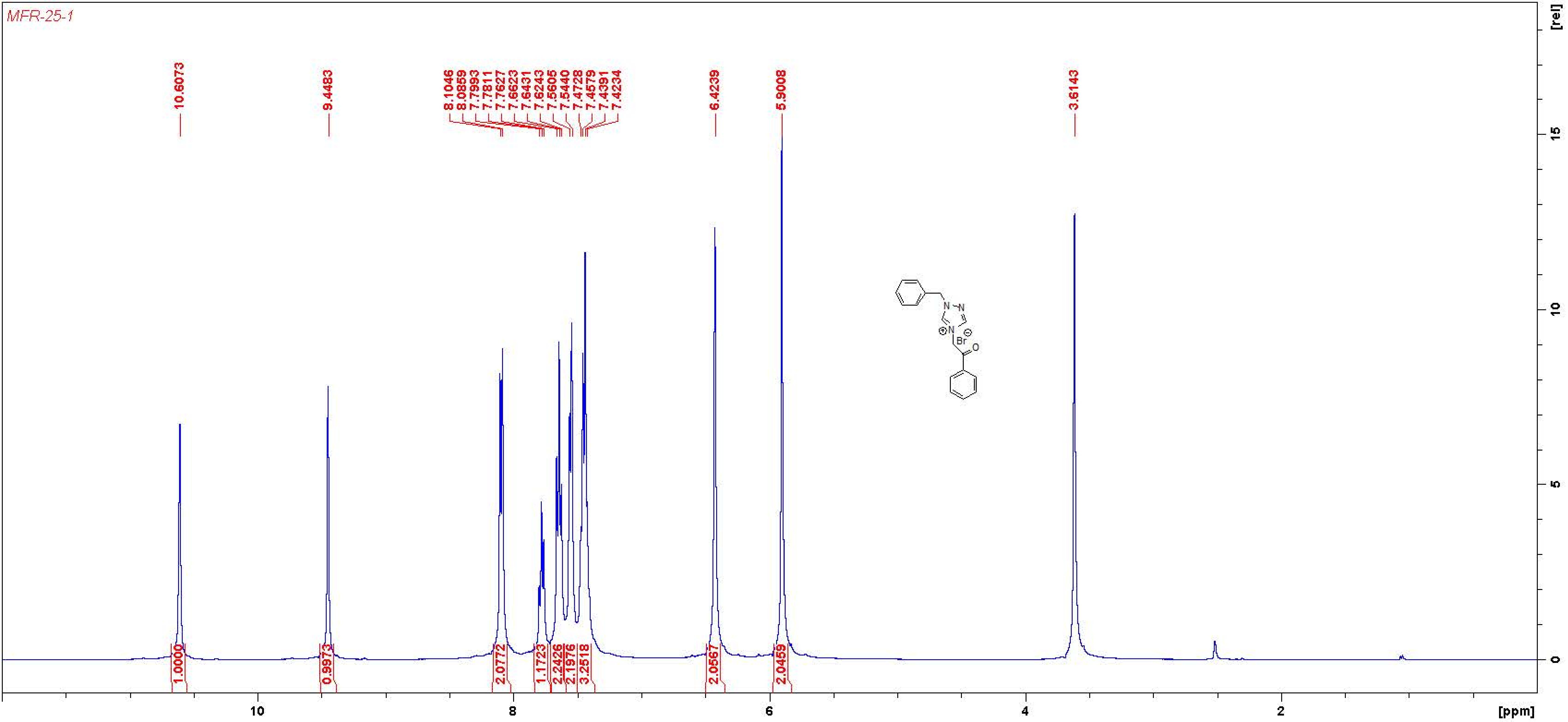

MFR-25-1
10.6073
9.4483
8.1046
8.0859
7.7993
7.7811
7.7627
7.6623
7.6431
7.6243
7.5605
7.5440
7.4728
7.4579
7.4391
7.4234
6.4239
5.9008
3.6143
1.0000
0.9973
2.0772
1.1723
2.2426
2.1976
3.2518
2.0567
2.0459
[rel]
15
10
5
0
10
8
6
4
2
[ppm]
N
N
N
Br
O

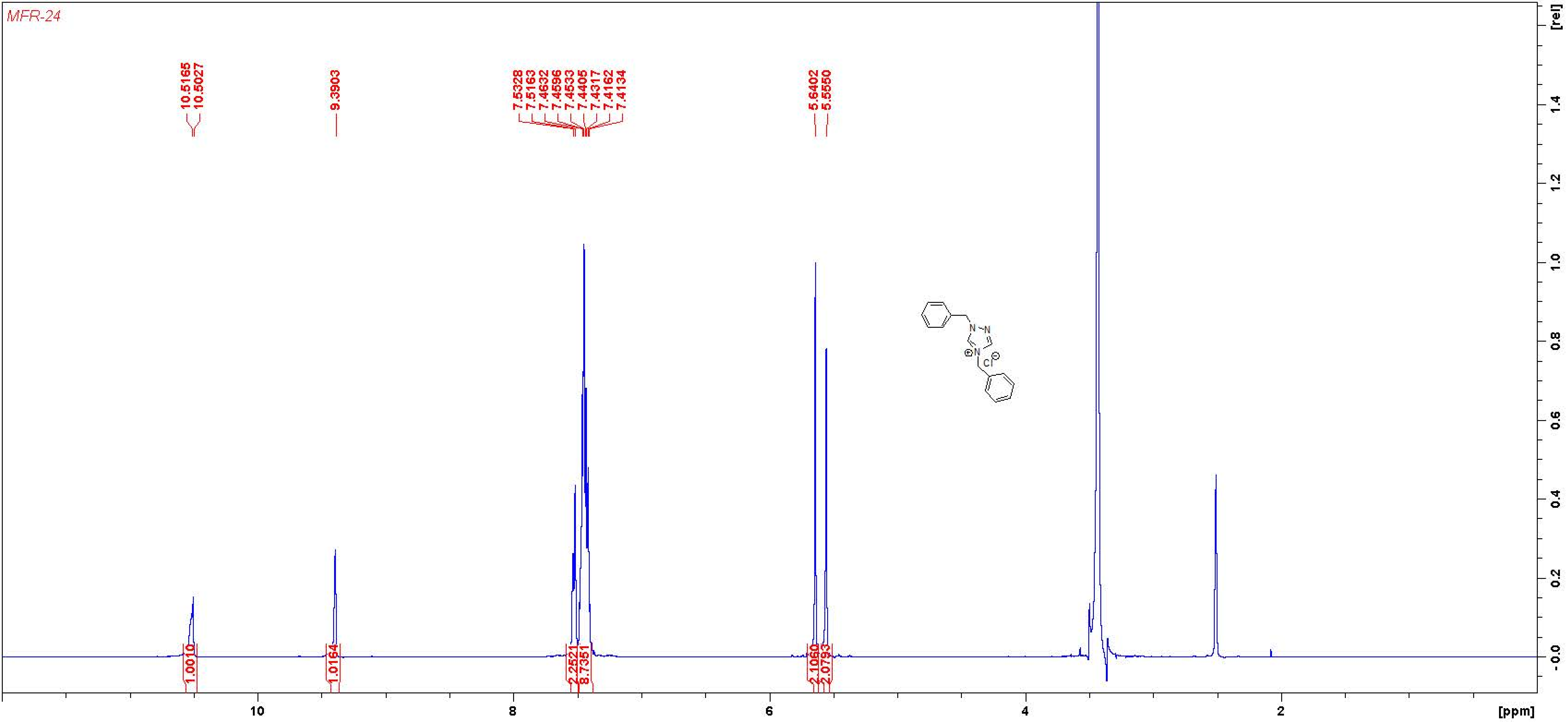

MFR-24
10.5165
10.5027
9.3903
7.5328
7.5163
7.4632
7.4596
7.4533
7.4405
7.4317
7.4162
7.4134
5.6402
5.5550
1.0010
1.0164
2.2521
8.7351
2.1060
2.0793
10
8
6
4
2
[ppm]
[rel]
1.4
1.2
1.0
0.8
0.6
0.4
0.2
-0.0

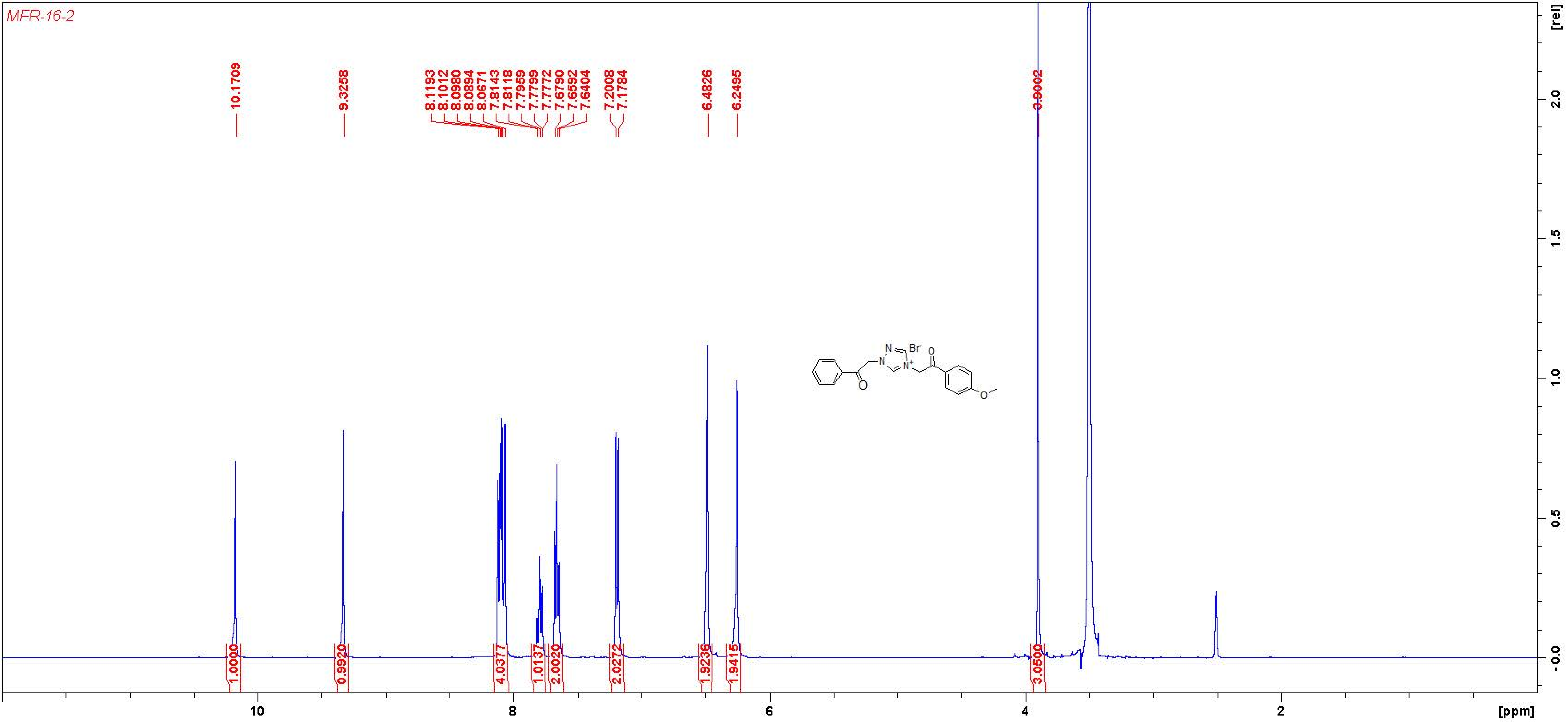

MFR-16-2
10.1709
9.3258
8.1193
8.1012
8.0980
8.0894
8.0671
7.8143
7.8118
7.7959
7.7799
7.7772
7.6790
7.6592
7.6404
7.2008
7.1784
6.4826
6.2495
3.9002
1.0000
0.9920
4.0377
1.0137
2.0020
2.0272
1.9236
1.9415
3.0500
Br⁻
N
N
N⁺
O
O
O
[rel]
2.0
1.5
1.0
0.5
-0.0
10
8
6
4
2
[ppm]

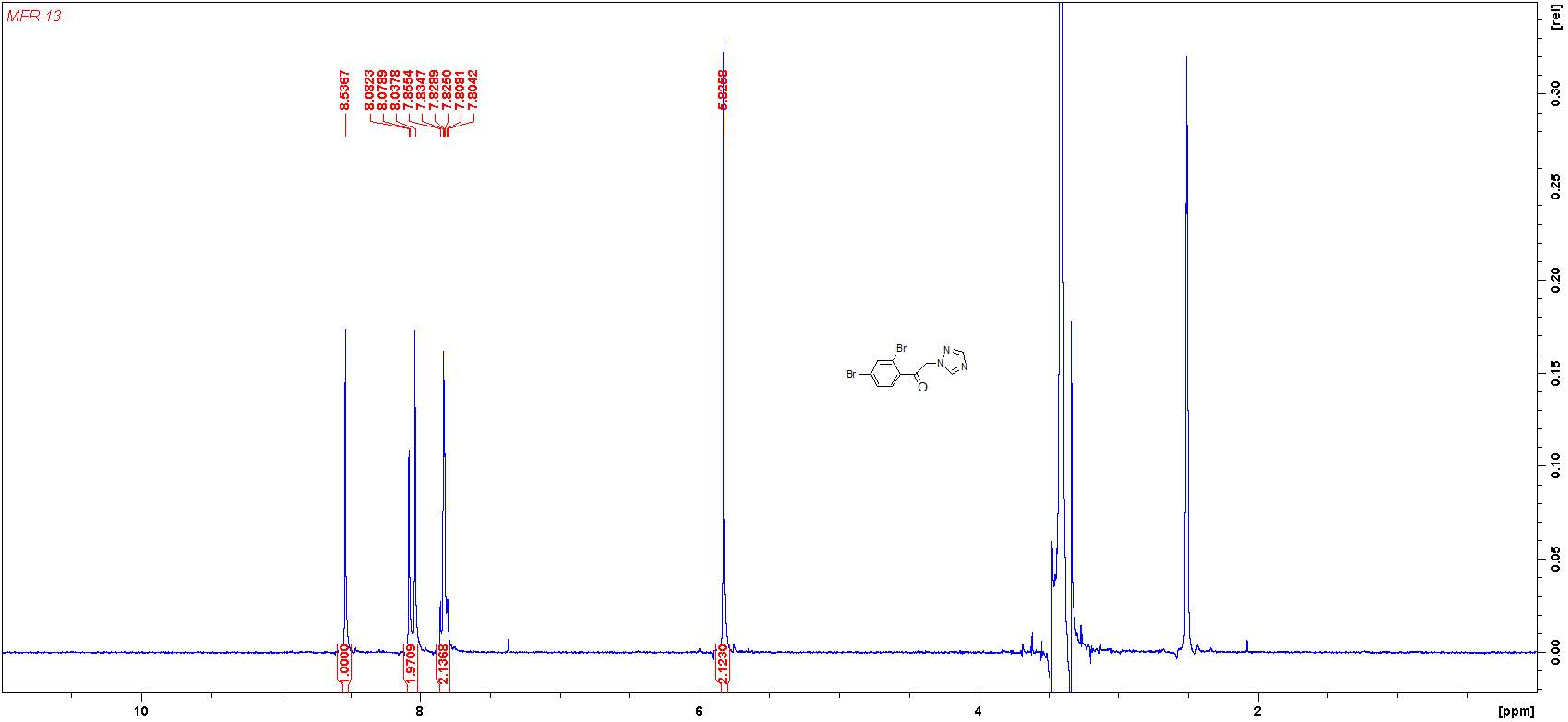
MFR-13
8.5367
8.0823
8.0789
8.0378
7.8554
7.8347
7.8289
7.8250
7.8081
7.8042
5.8258
1.0000
1.9709
2.1368
2.1230
Br
Br
N
N
N
O
[rel]
0.30
0.25
0.20
0.15
0.10
0.05
0.00
10
8
6
4
2
[ppm]

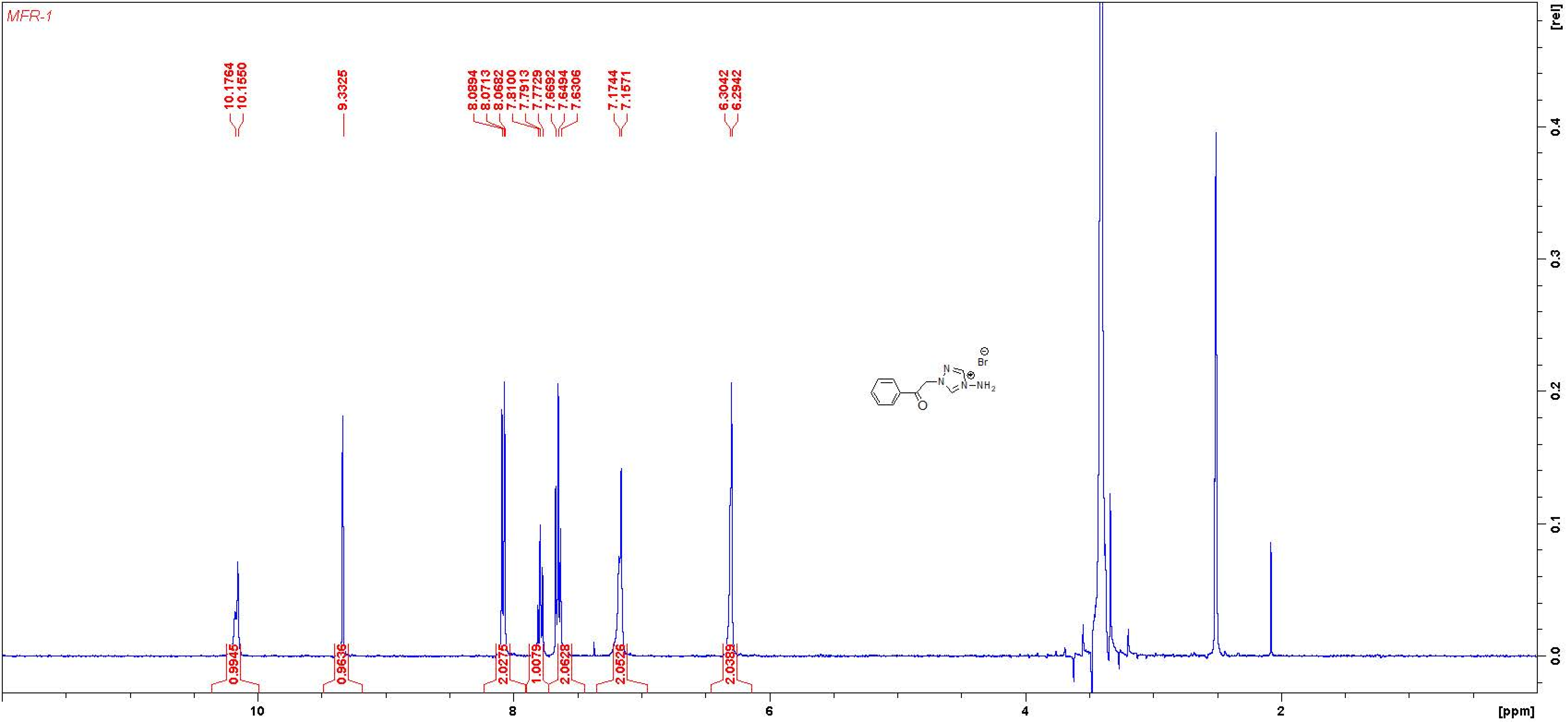

MFR-1
10.1764
10.1550
9.3325
8.0894
8.0713
8.0682
7.8100
7.7913
7.7729
7.6692
7.6494
7.6306
7.1744
7.1571
6.3042
6.2942
0.9945
0.9636
2.0275
1.0079
2.0628
2.0526
2.0389
N
N
N
Br
NH2
O
[rel]
0.0
0.1
0.2
0.3
0.4
10
8
6
4
2
[ppm]

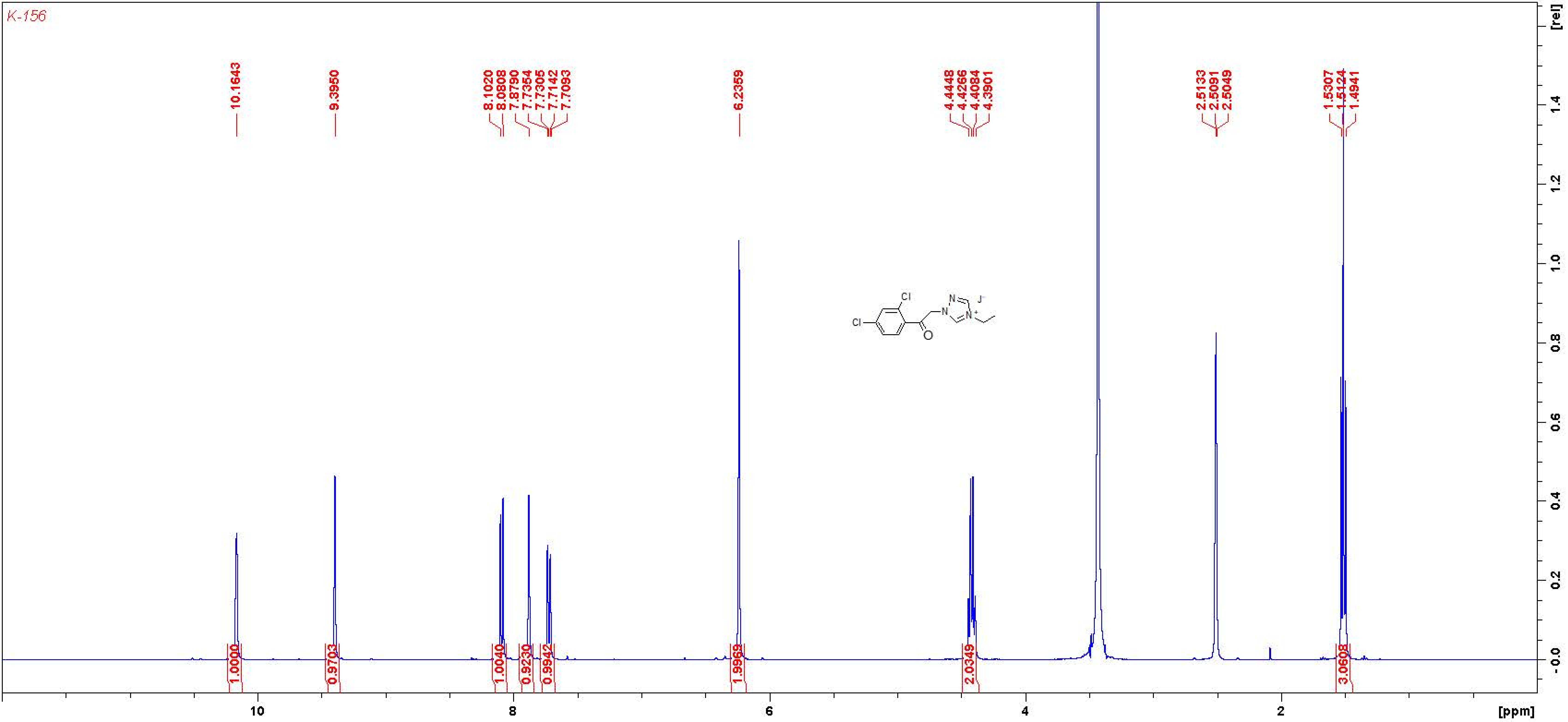
K-156
10.1643
9.3950
8.1020
8.0808
7.8790
7.7354
7.7305
7.7142
7.7093
6.2359
4.4448
4.4266
4.4084
4.3901
2.5133
2.5091
2.5049
1.5307
1.5124
1.4941
Cl
Cl
N
N
N+
J−
O
1.0000
0.9703
1.0040
0.9230
0.9942
1.9969
2.0349
3.0608
[rel]
[ppm]
10
8
6
4
2
1.4
1.2
1.0
0.8
0.6
0.4
0.2
-0.0

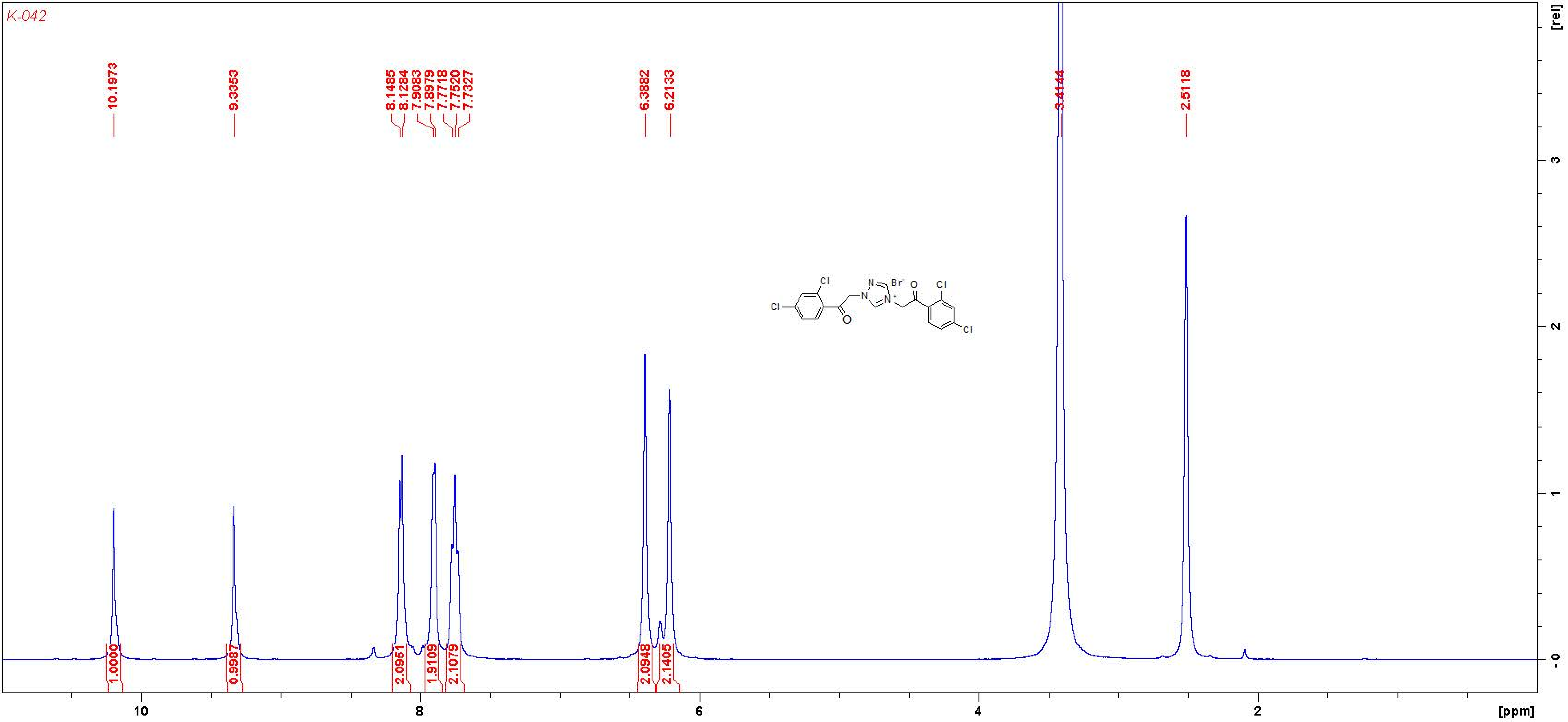
K-042
10.1973
9.3353
8.1485
8.1284
7.9083
7.8979
7.7718
7.7520
7.7327
6.3882
6.2133
3.4144
2.5118
1.0000
0.9987
2.0951
1.9109
2.1079
2.0948
2.1405
Cl
Cl
N
N
Br
N+
O
O
Cl
Cl
10
8
6
4
2
[ppm]
[rel]
3
2
1
0

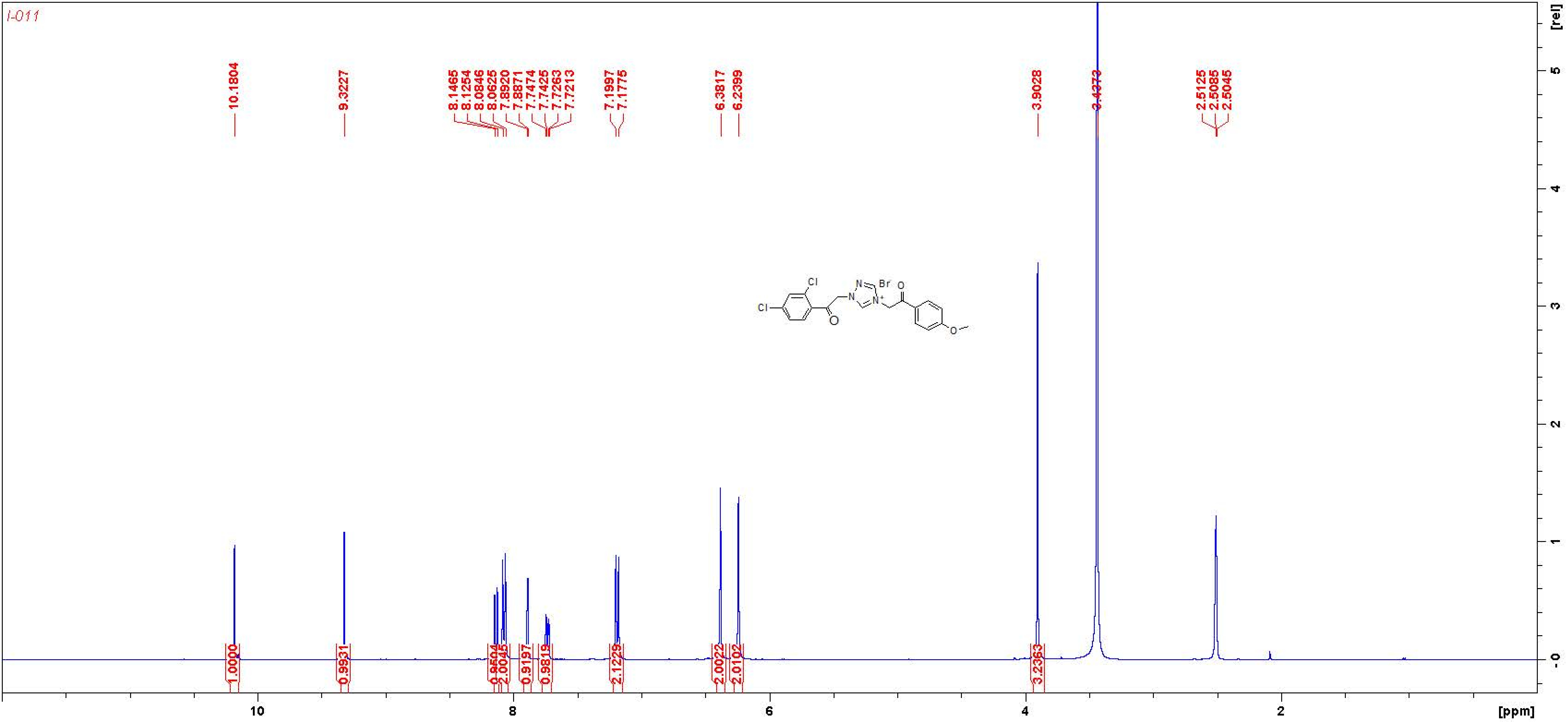
I-011
10.1804
9.3227
8.1465
8.1254
8.0846
8.0625
7.8920
7.8871
7.7474
7.7425
7.7263
7.7213
7.1997
7.1775
6.3817
6.2399
3.9028
3.4373
2.5125
2.5085
2.5045
Cl
Cl
N
N
N+
Br-
O
O
O
1.0000
0.9931
0.9504
2.0045
0.9197
0.9819
2.1229
2.0022
2.0102
3.2363
[rel]
[ppm]

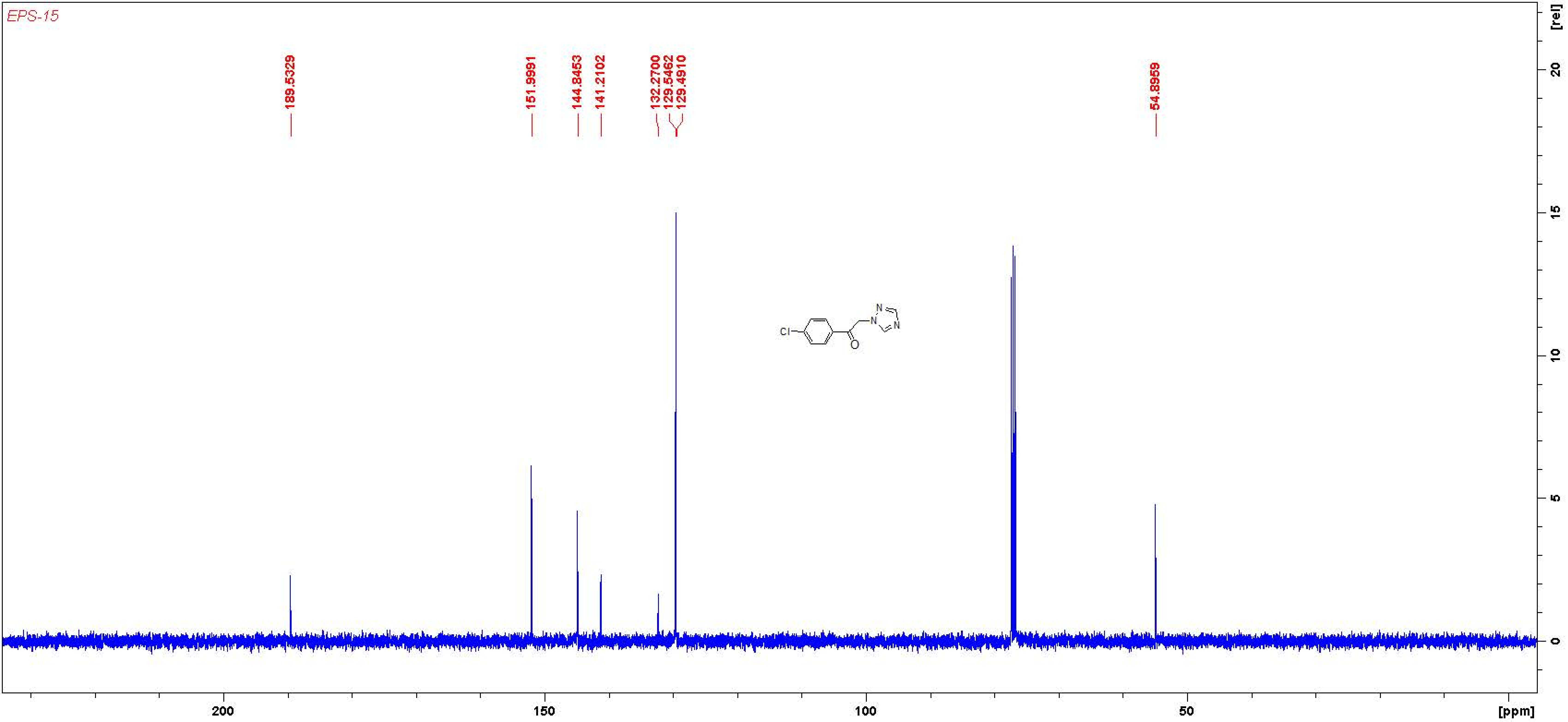
EPS-15
189.5329
151.9991
144.8453
141.2102
132.2700
129.5462
129.4910
54.8959
Cl
O
N
N
N
200
150
100
50
[ppm]
[rel]
20
15
10
5
0

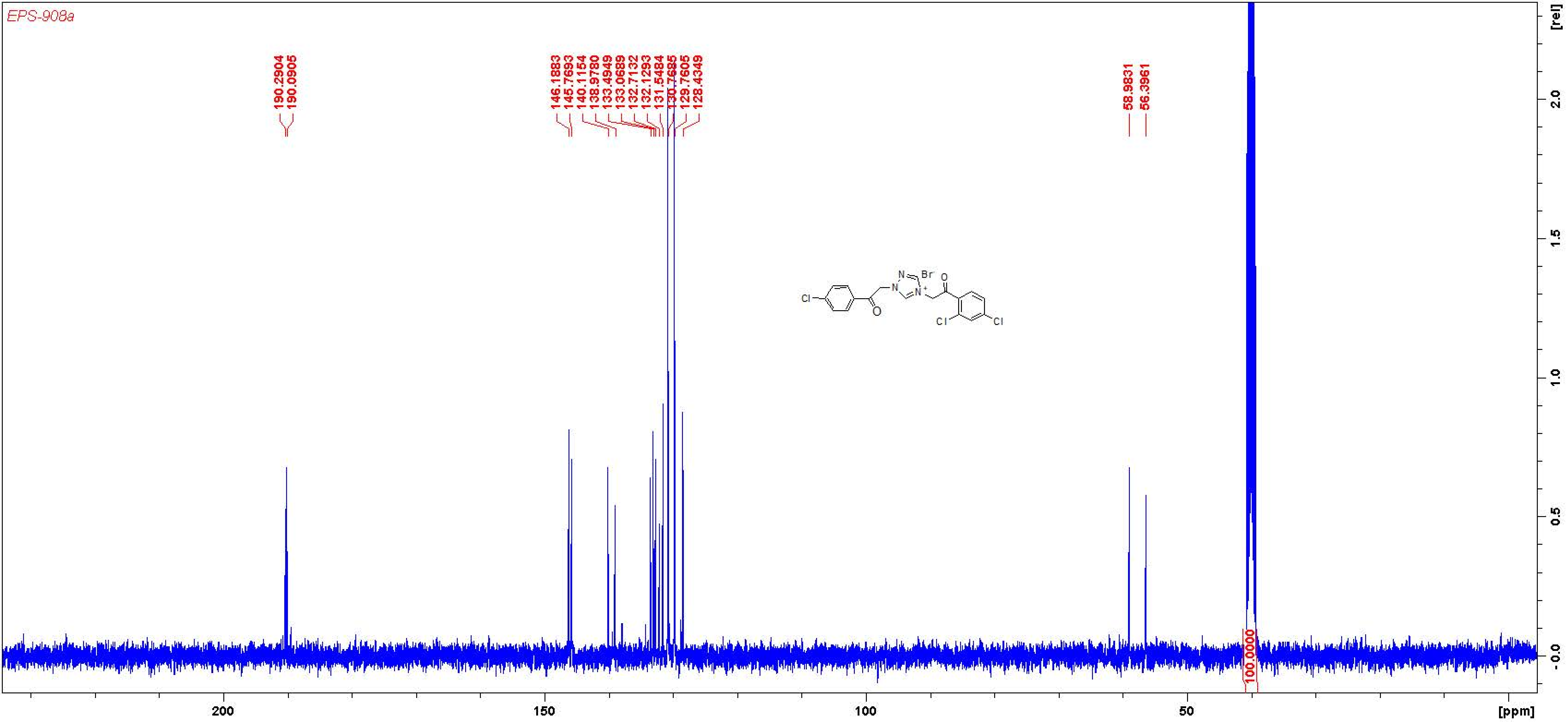
EPS-908a
190.2904
190.0905
146.1883
145.7693
140.1154
138.9780
133.4949
133.0689
132.7132
132.1293
131.5484
130.7685
129.7605
128.4349
58.9831
56.3961
Br⁻
N
N
N⁺
O
O
Cl
Cl
Cl
100.0000
[rel]
2.0
1.5
1.0
0.5
-0.0
200
150
100
50
[ppm]

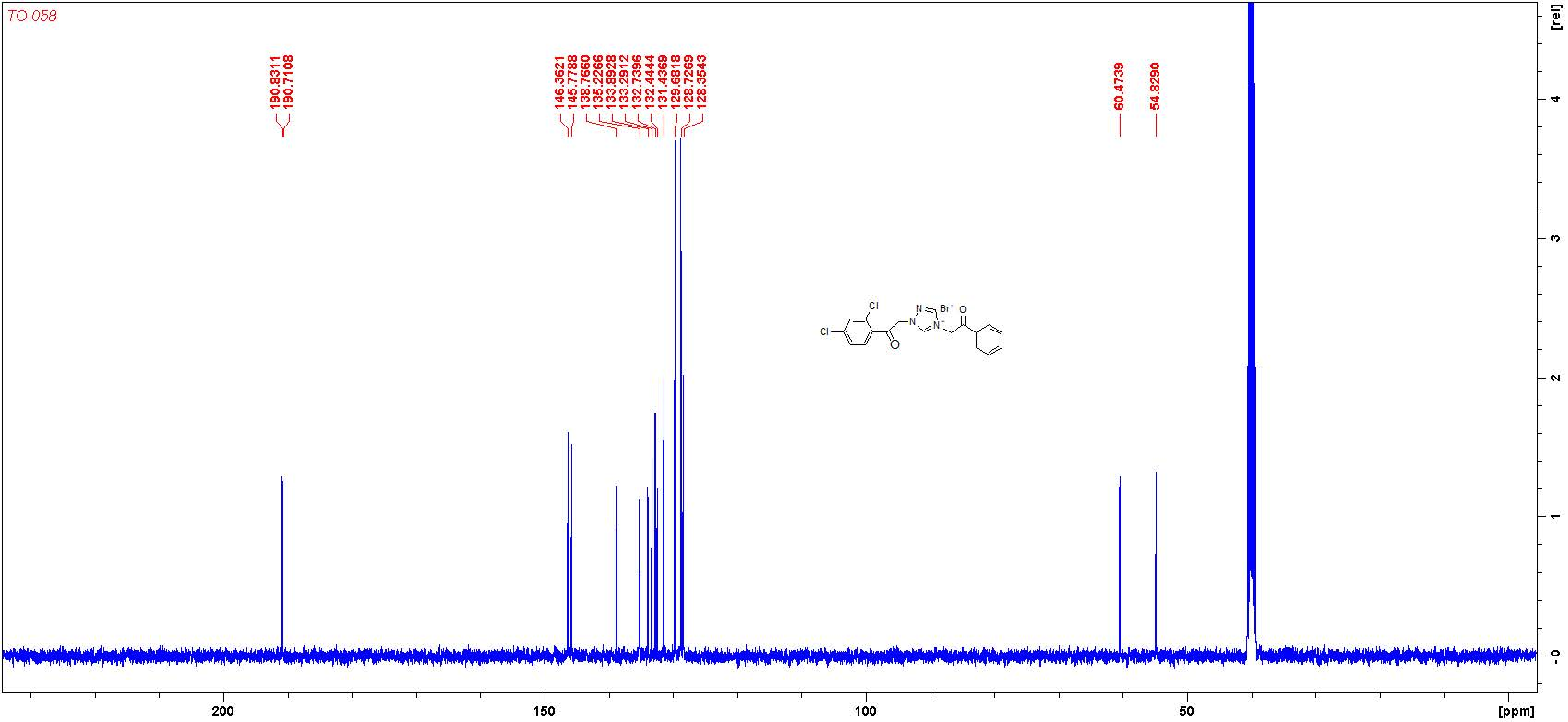
TO-058
190.8311
190.7108
146.3621
145.7788
138.7660
135.2266
133.8928
133.2912
132.7396
132.4444
131.4369
129.6818
128.7269
128.3543
60.4739
54.8290
Cl
Cl
N
Br⁻
N⁺
N
O
O
200
150
100
50
[ppm]
[rel]
0
1
2
3
4

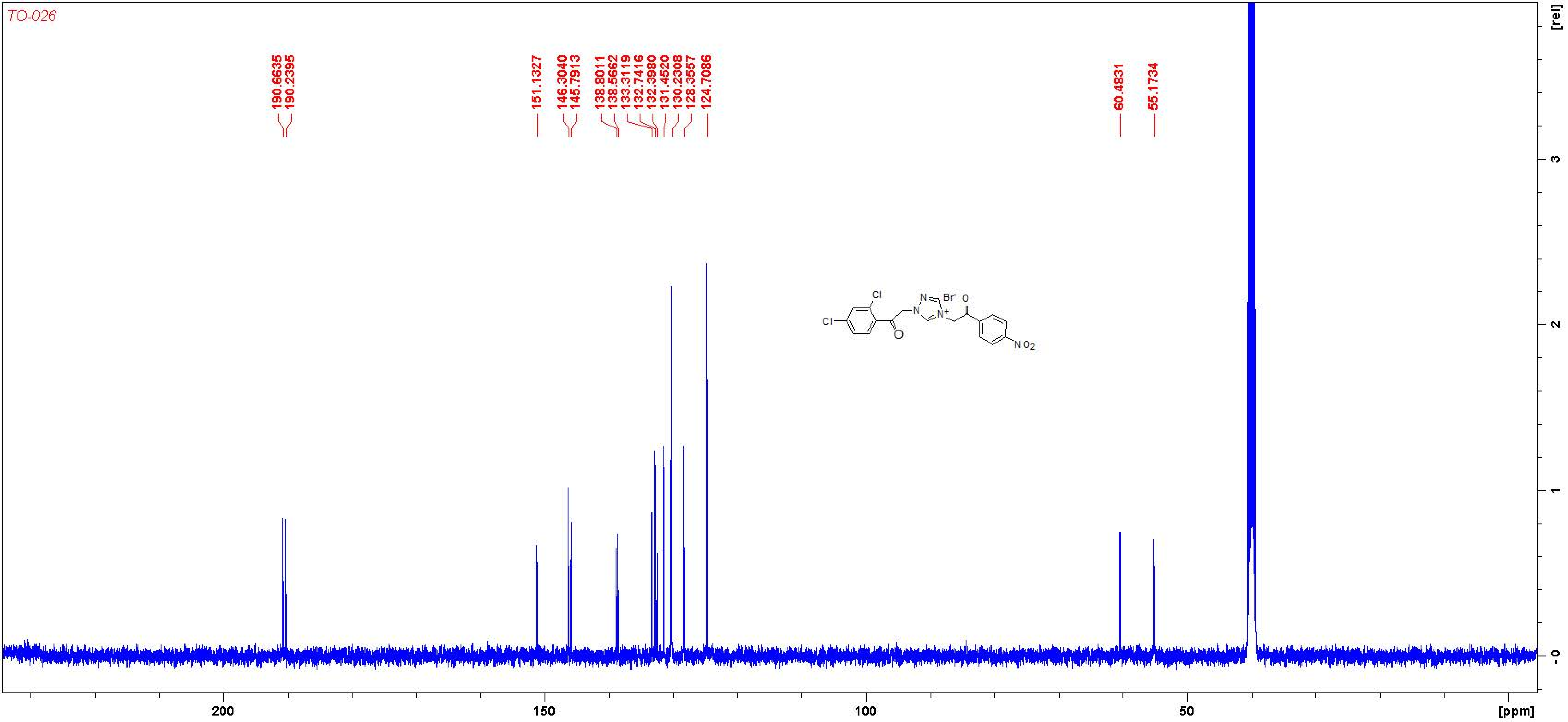
TO-026
190.6635
190.2395
151.1327
146.3040
145.7913
138.8011
138.5662
133.3119
132.7416
132.3980
131.4520
130.2308
128.3557
124.7086
60.4831
55.1734
Cl
Cl
N
N
N+
Br-
O
O
NO2
200
150
100
50
[ppm]
[rel]
3
2
1
0

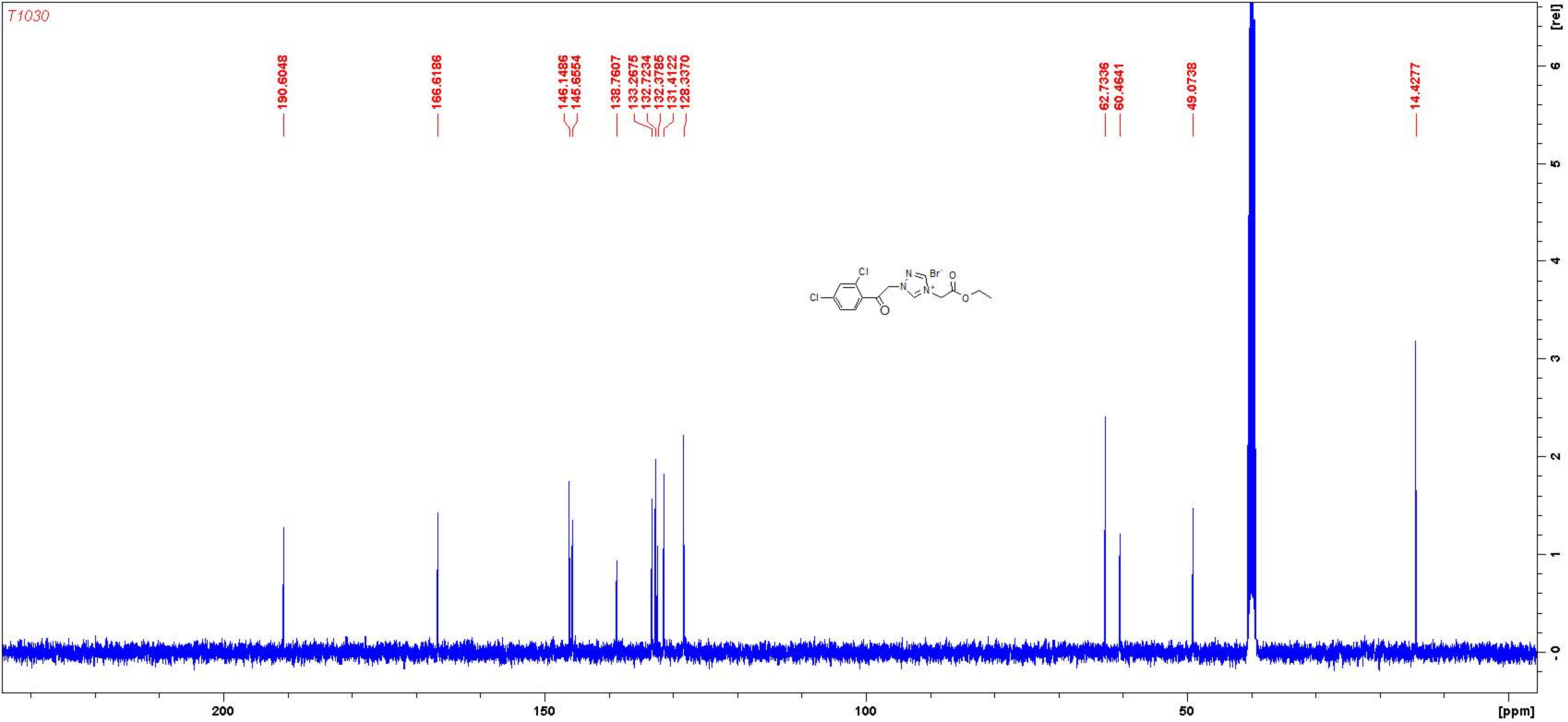
T1030
190.6048
166.6186
146.1486
145.6554
138.7607
133.2675
132.7234
132.3785
131.4122
128.3370
62.7336
60.4641
49.0738
14.4277
Cl
Cl
N
N
N+
Br-
O
O
O
[rel]
6
5
4
3
2
1
0
200
150
100
50
[ppm]

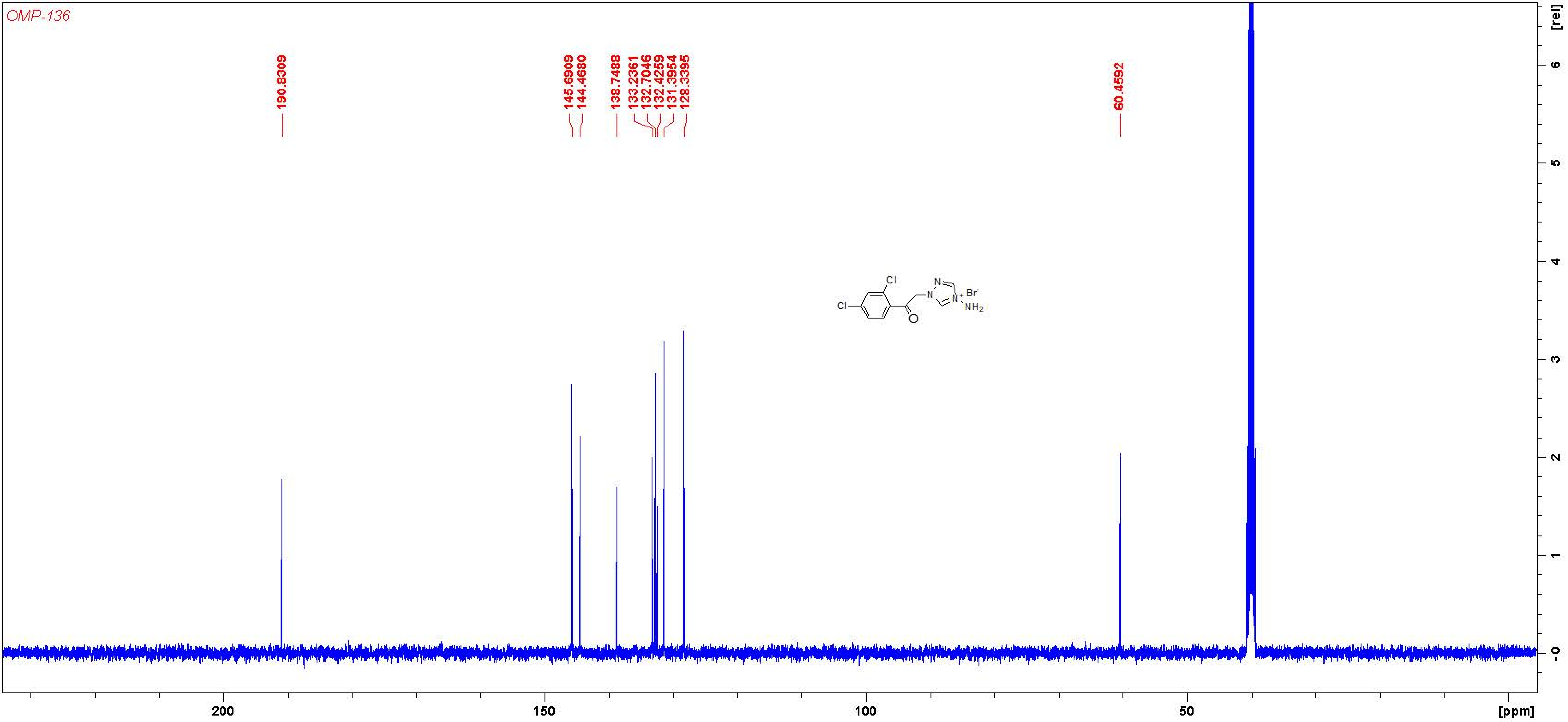
OMP-136
190.8309
145.6909
144.4680
138.7488
133.2361
132.7046
132.4259
131.3954
128.3395
60.4592
Cl
Cl
N
N
N+
Br-
NH2
O
200
150
100
50
[ppm]
[rel]
6
5
4
3
2
1
0

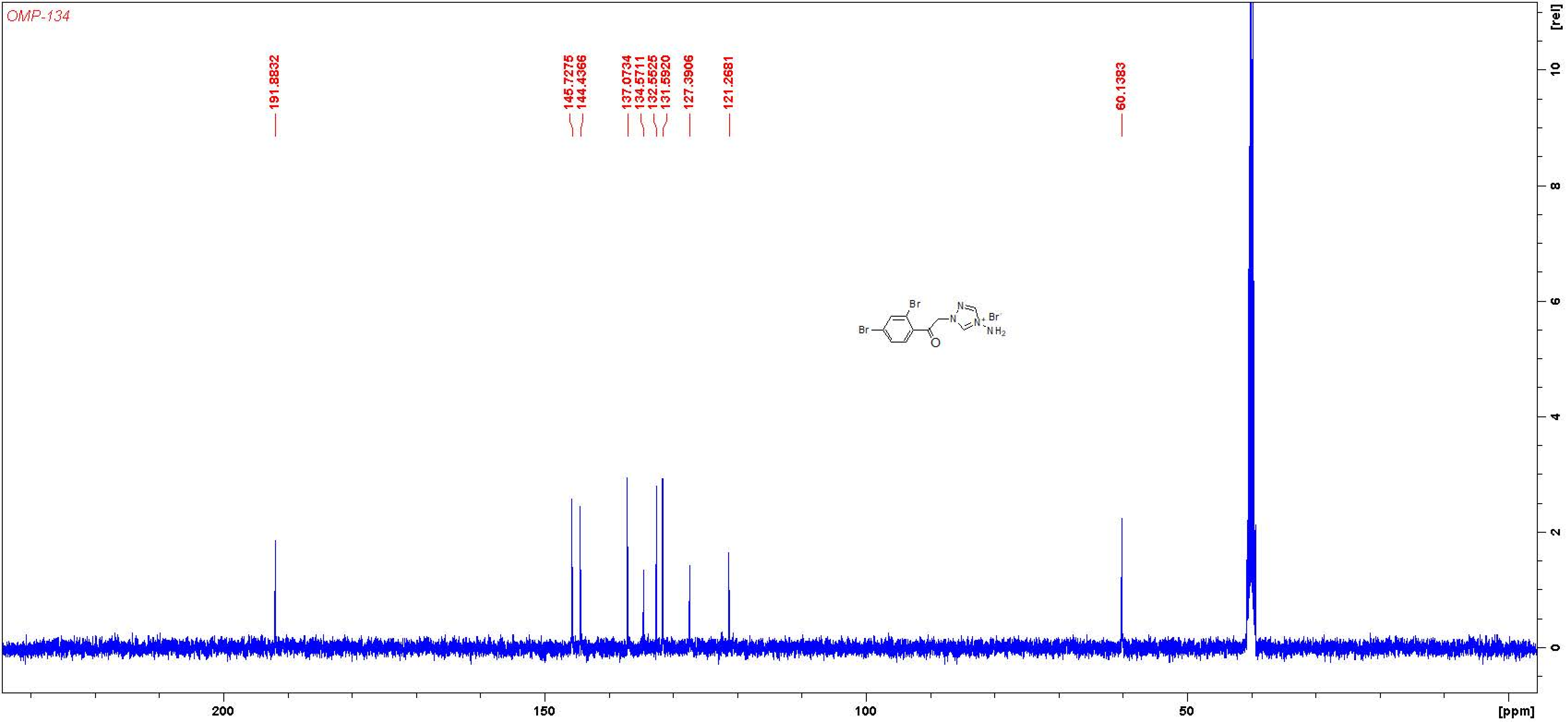
OMP-134
191.8832
145.7275
144.4366
137.0734
134.5711
132.5525
131.5920
127.3906
121.2681
60.1383
Br
Br
N
N
N+
Br-
NH2
O
[rel]
10
8
6
4
2
0
200
150
100
50
[ppm]

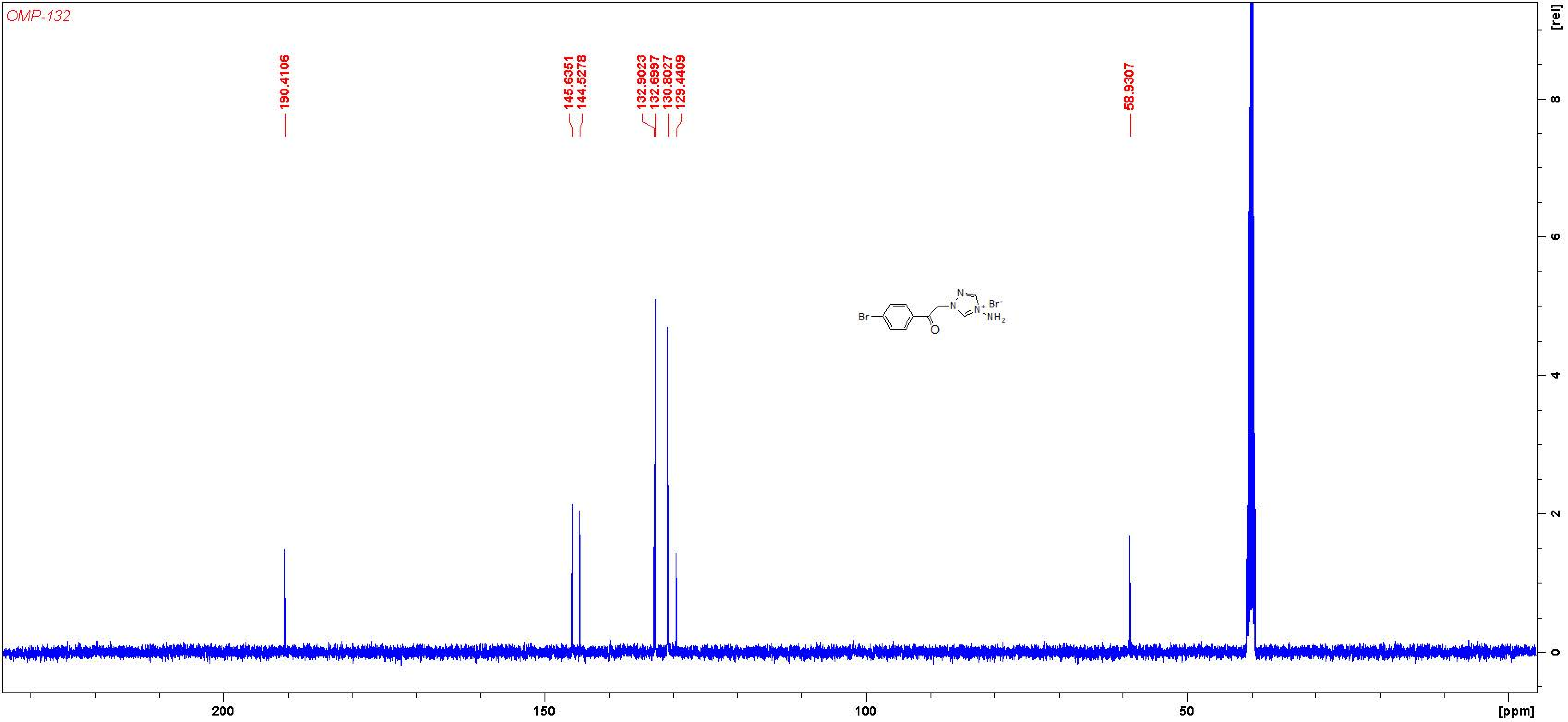
OMP-132
190.4106
145.6351
144.5278
132.9023
132.6997
130.8027
129.4409
58.9307
Br
N
N
N
+
Br⁻
NH2
O
[rel]
8
6
4
2
0
200
150
100
50
[ppm]

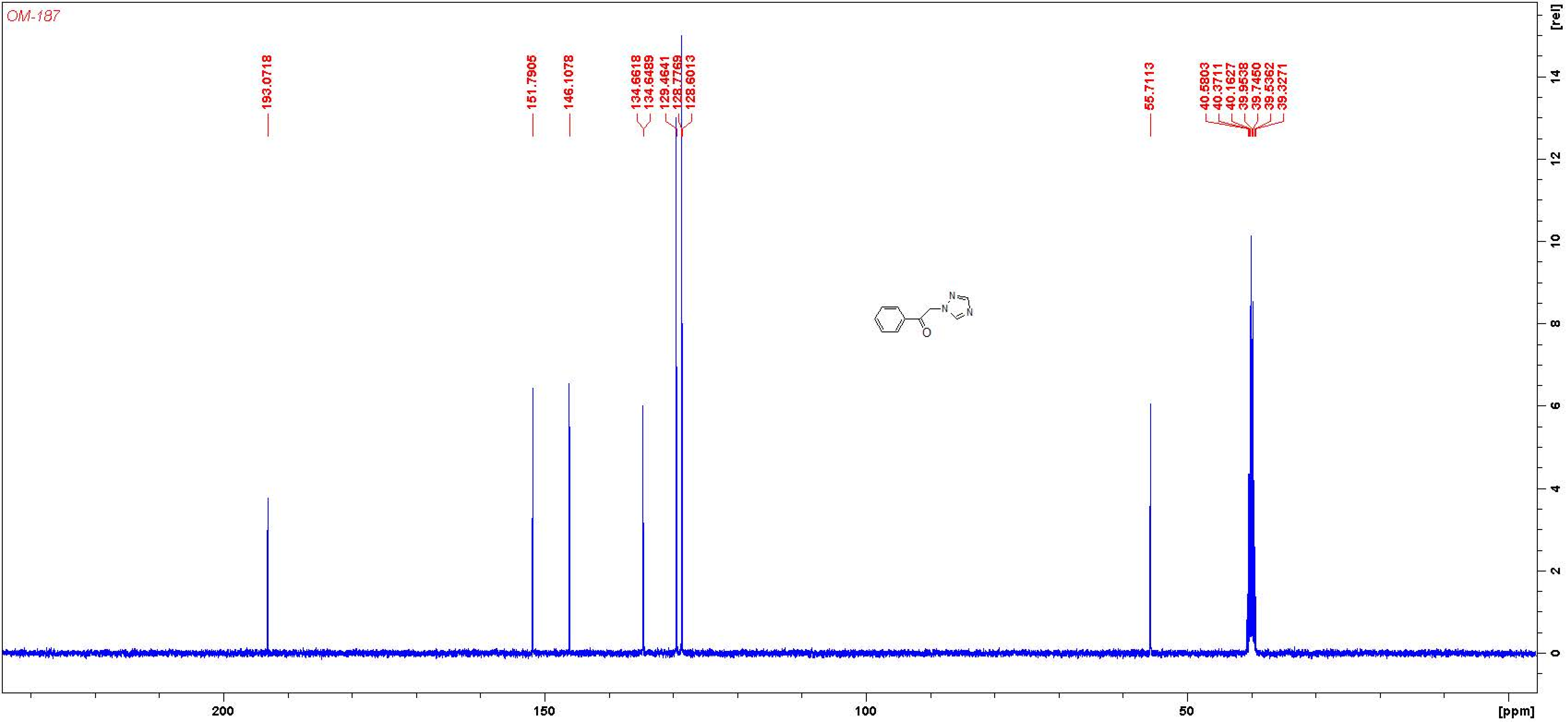
OM-187
193.0718
151.7905
146.1078
134.6618
134.6489
129.4641
128.7769
128.6013
55.7113
40.5803
40.3711
40.1627
39.9538
39.7450
39.5362
39.3271
N
N
N
O
[rel]
[ppm]
200
150
100
50
0
2
4
6
8
10
12
14

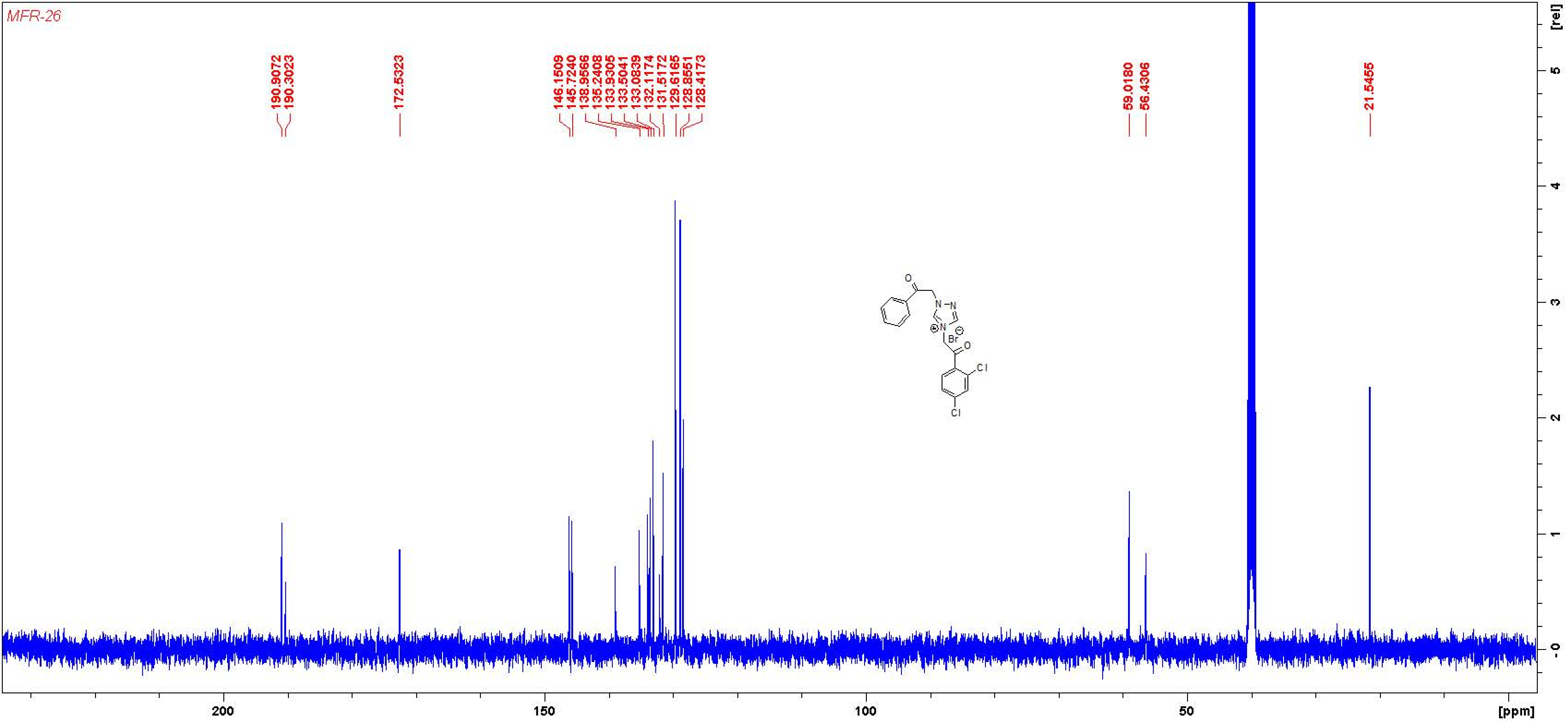
MFR-26
190.9072
190.3023
172.5323
146.1509
145.7240
138.9566
135.2408
133.9305
133.5041
133.0839
132.1174
131.5172
129.6165
128.8551
128.4173
59.0180
56.4306
21.5455
[rel]
[ppm]
200
150
100
50
Br
Cl
Cl

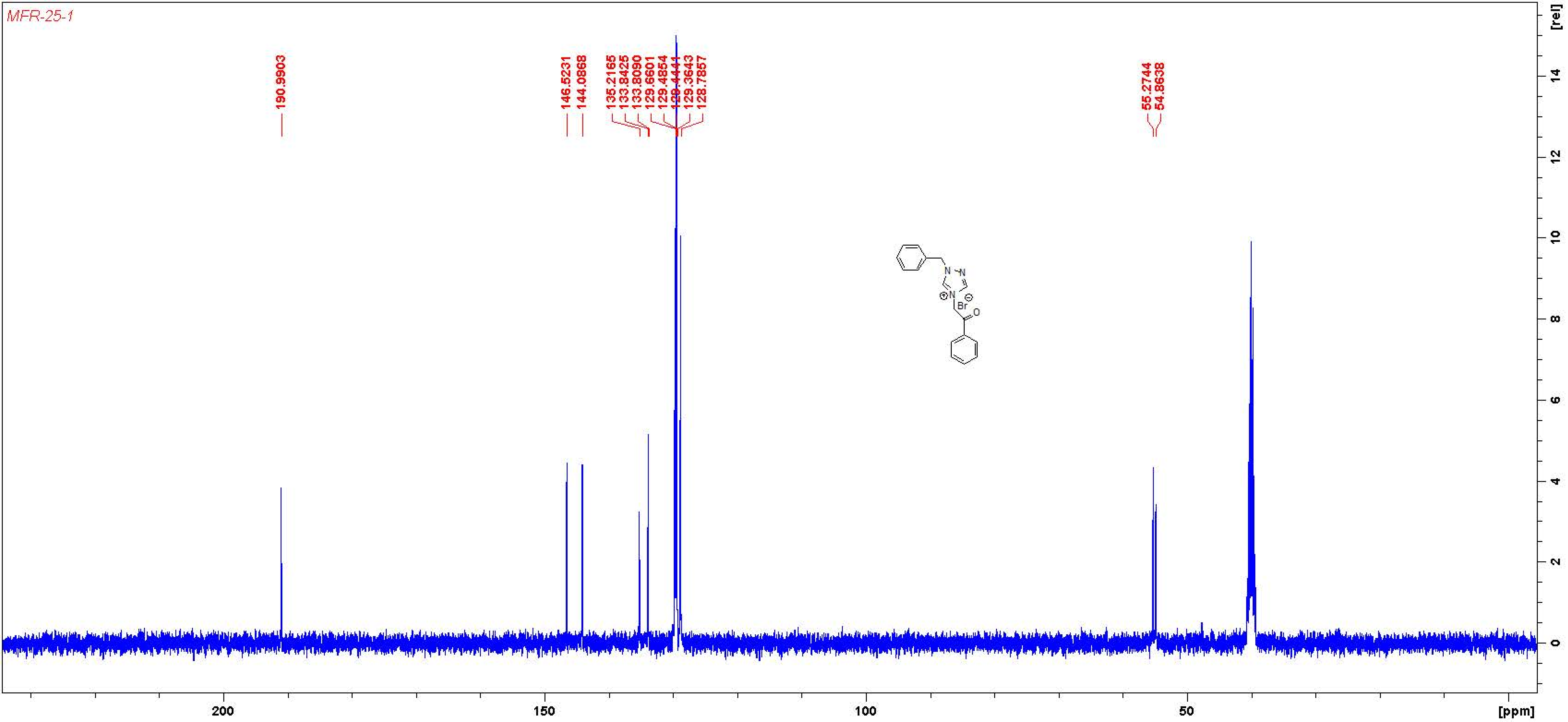
MFR-25-1
190.9903
146.5231
144.0868
135.2165
133.8425
133.8090
129.6601
129.4854
129.4441
129.3643
128.7857
55.2744
54.8638
N
N
N
Br
O
200
150
100
50
[ppm]
[rel]
0
2
4
6
8
10
12
14

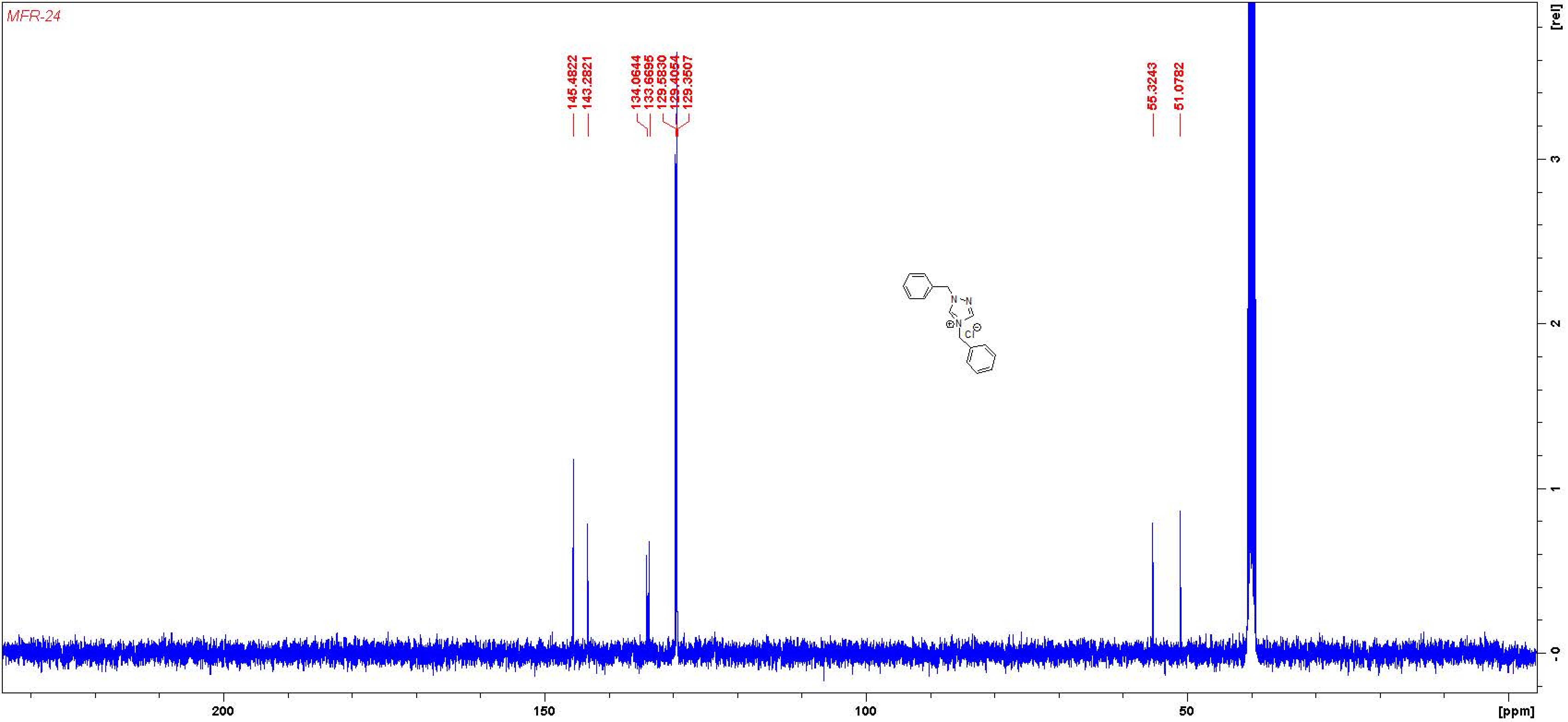

MFR-24
145.4822
143.2821
134.0644
133.6695
129.5830
129.4054
129.3507
55.3243
51.0782
N
N
N
Cl
[rel]
3
2
1
0
200
150
100
50
[ppm]

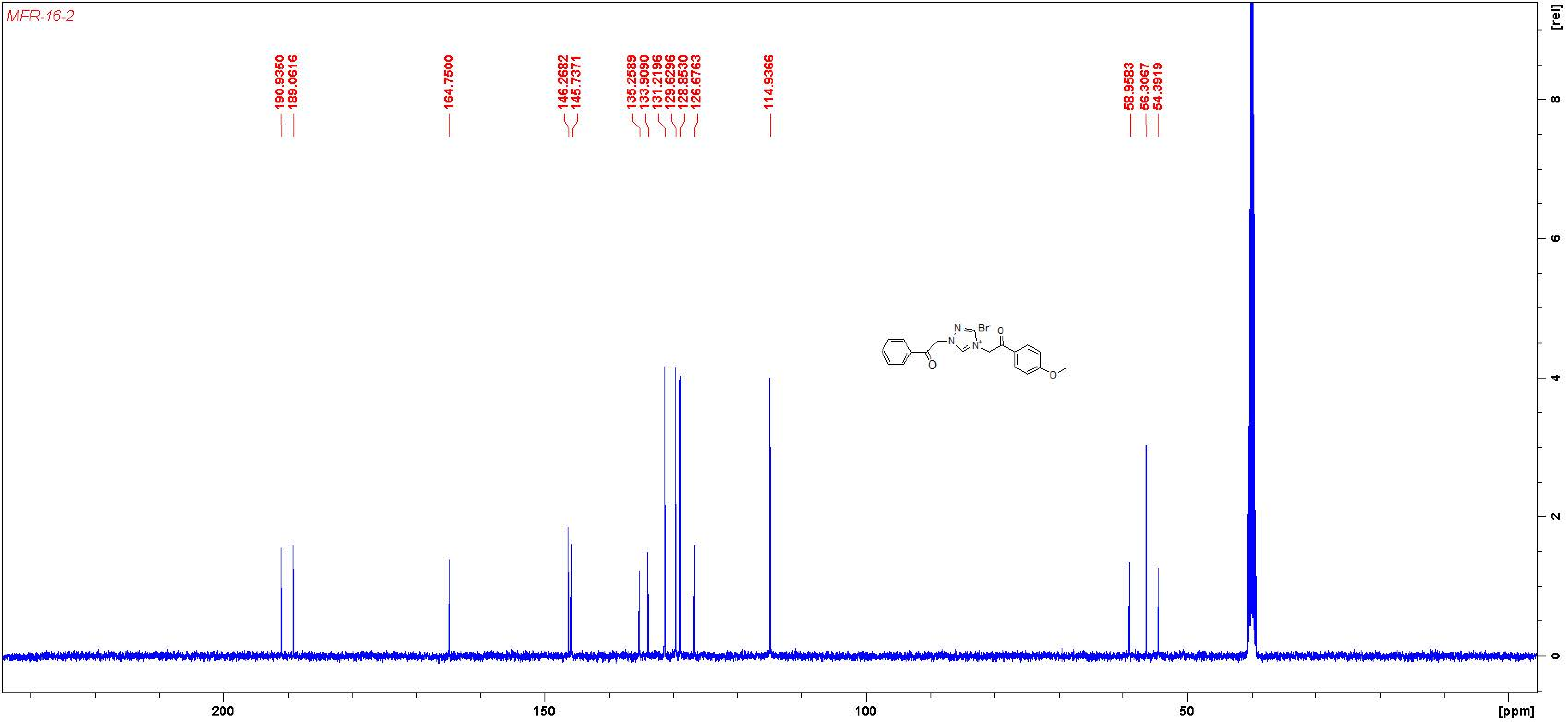
MFR-16-2
190.9350
189.0616
164.7500
146.2682
145.7371
135.2589
133.9090
131.2196
129.6296
128.8530
126.6763
114.9366
58.9583
56.3067
54.3919
[rel]
[ppm]
200
150
100
50

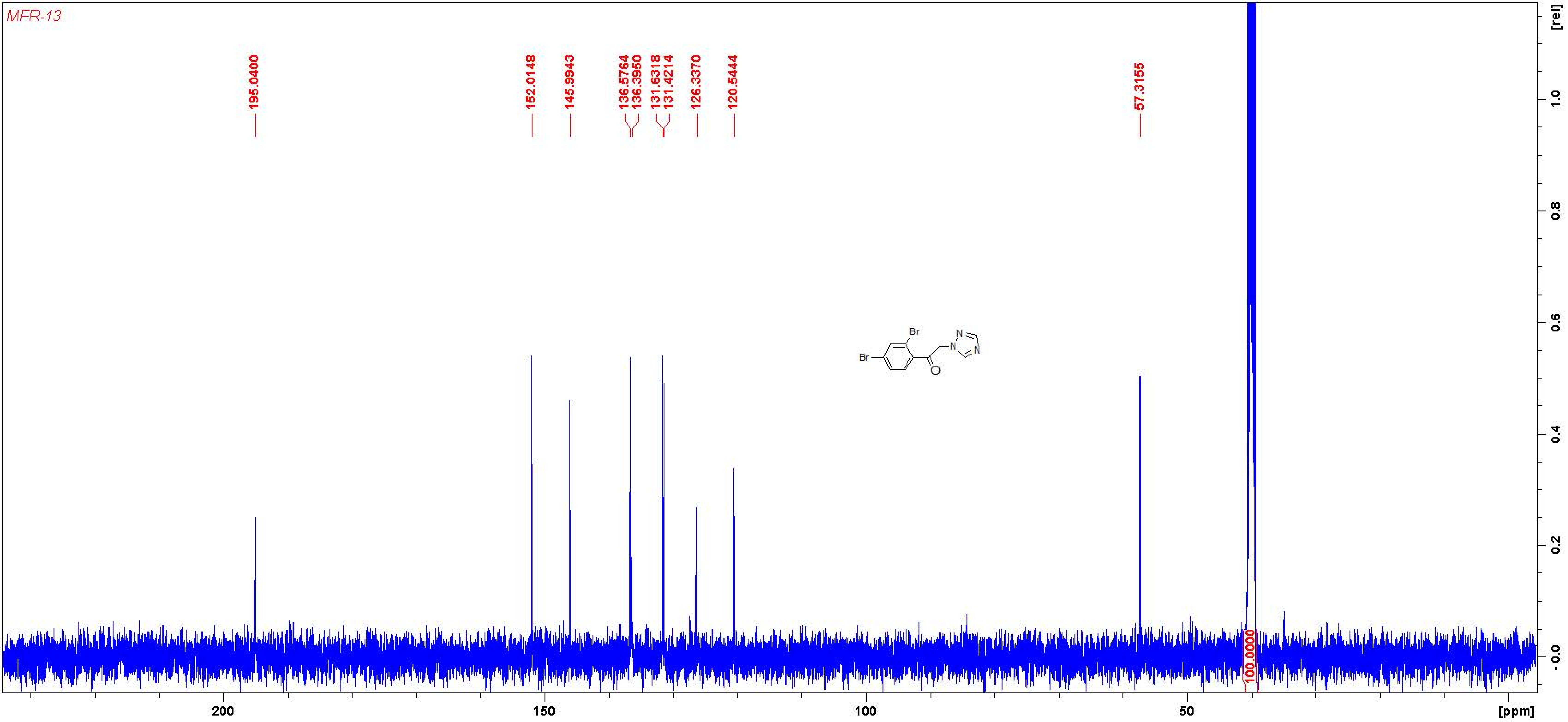
MFR-13
195.0400
152.0148
145.9943
136.5764
136.3950
131.6318
131.4214
126.3370
120.5444
57.3155
100.0000
Br
Br
N
N
N
O
200
150
100
50
[ppm]
[rel]
1.0
0.8
0.6
0.4
0.2
-0.0

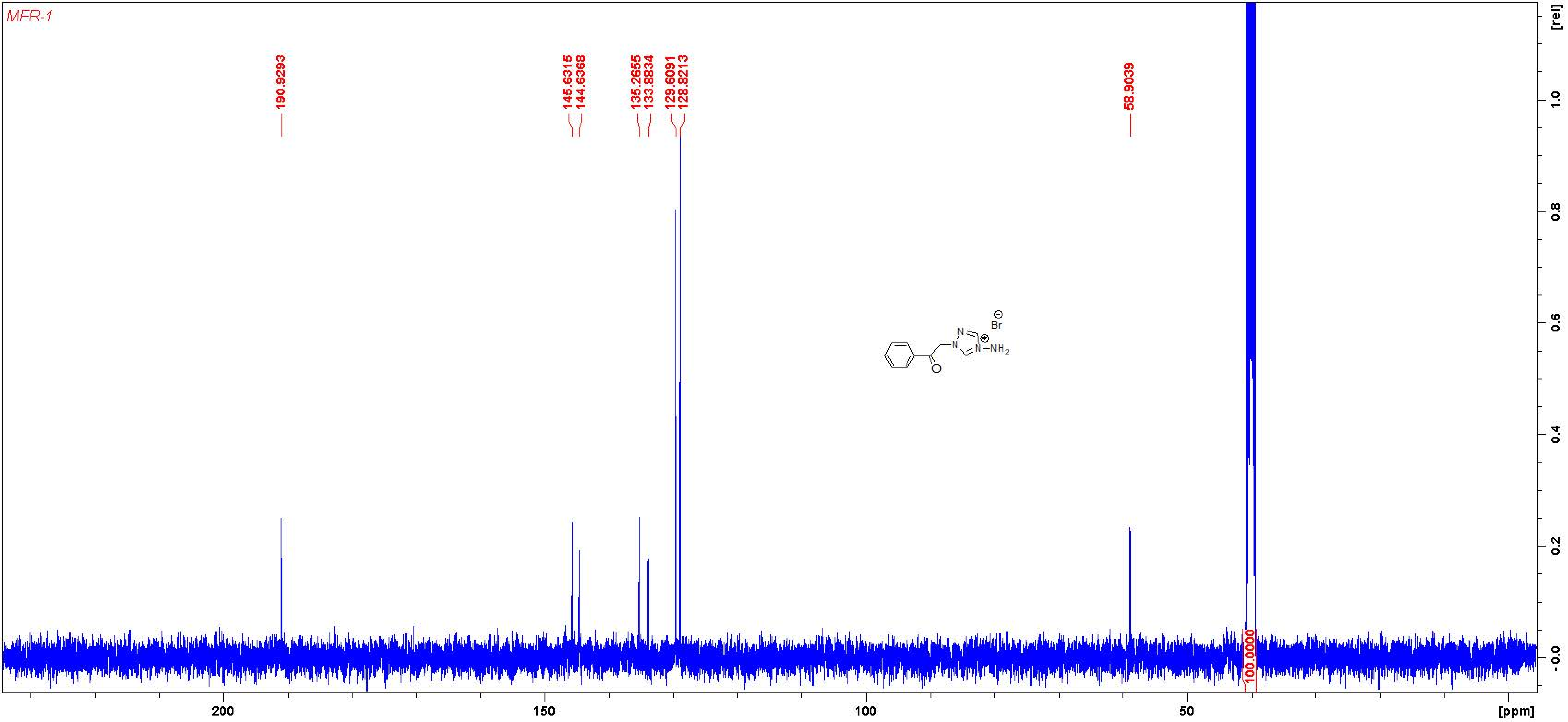
MFR-1
190.9293
145.6315
144.6368
135.2655
133.8834
129.6091
128.8213
58.9039
100.0000
Br
N
N
N
NH2
O
[rel]
[ppm]
200
150
100
50

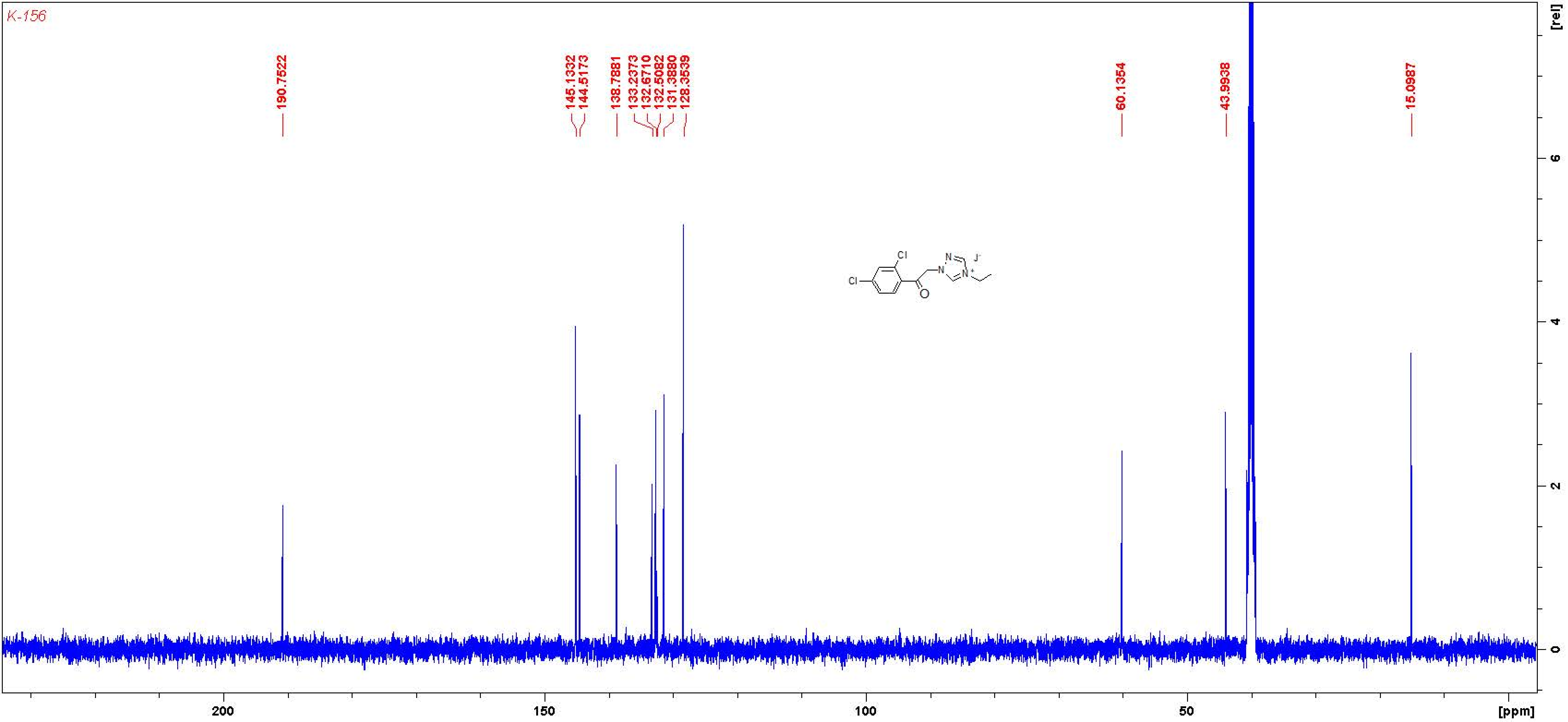
K-156
190.7522
145.1332
144.5173
138.7881
133.2373
132.6710
132.5082
131.3880
128.3539
60.1354
43.9938
15.0987
Cl
Cl
N
N
N+
J-
O
[rel]
6
4
2
0
200
150
100
50
[ppm]

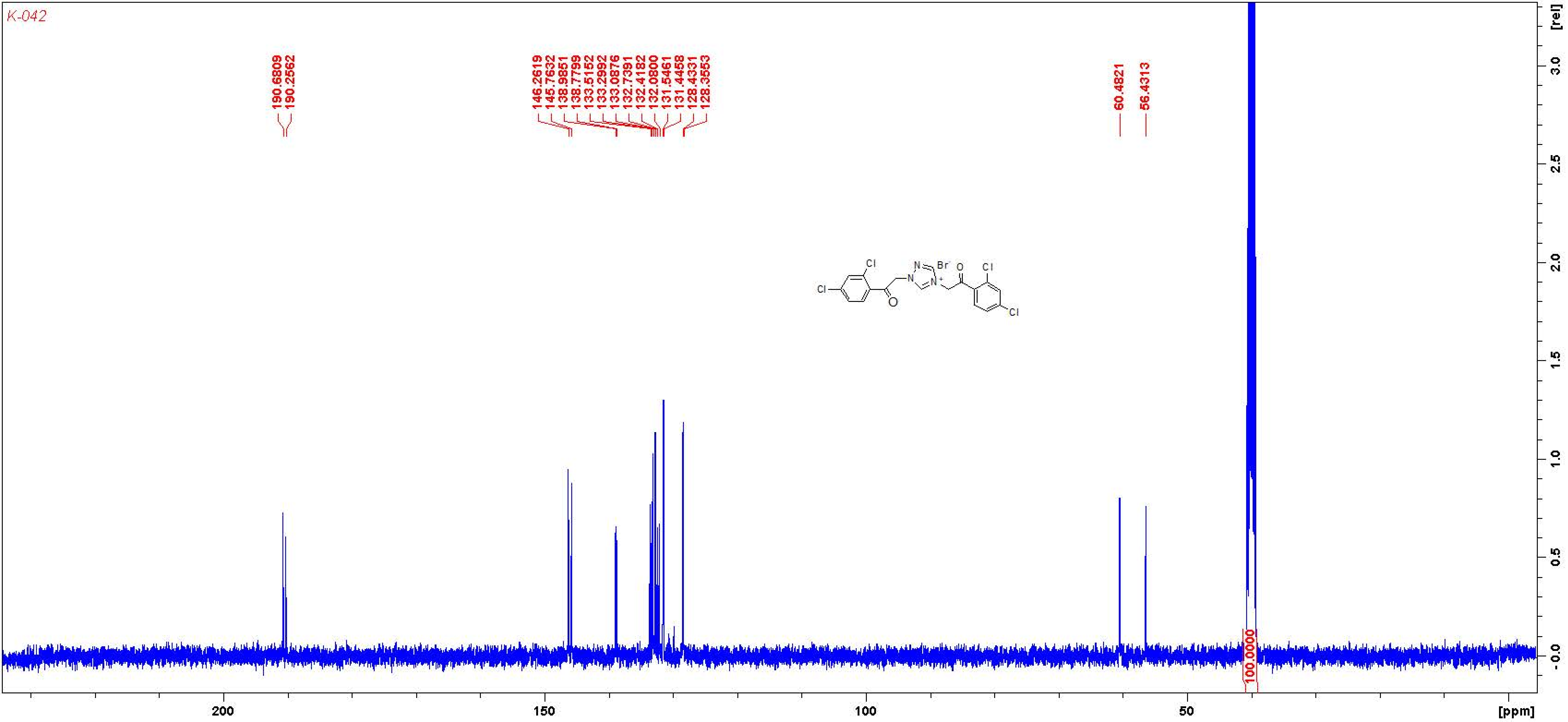
K-042
190.6809
190.2562
146.2619
145.7632
138.9851
138.7799
133.5152
133.2992
133.0876
132.7391
132.4182
132.0800
131.5461
131.4458
128.4331
128.3553
60.4821
56.4313
Cl
Cl
N
Br
O
Cl
N
N
O
Cl
100.0000
200
150
100
50
[ppm]
[rel]
3.0
2.5
2.0
1.5
1.0
0.5
-0.0

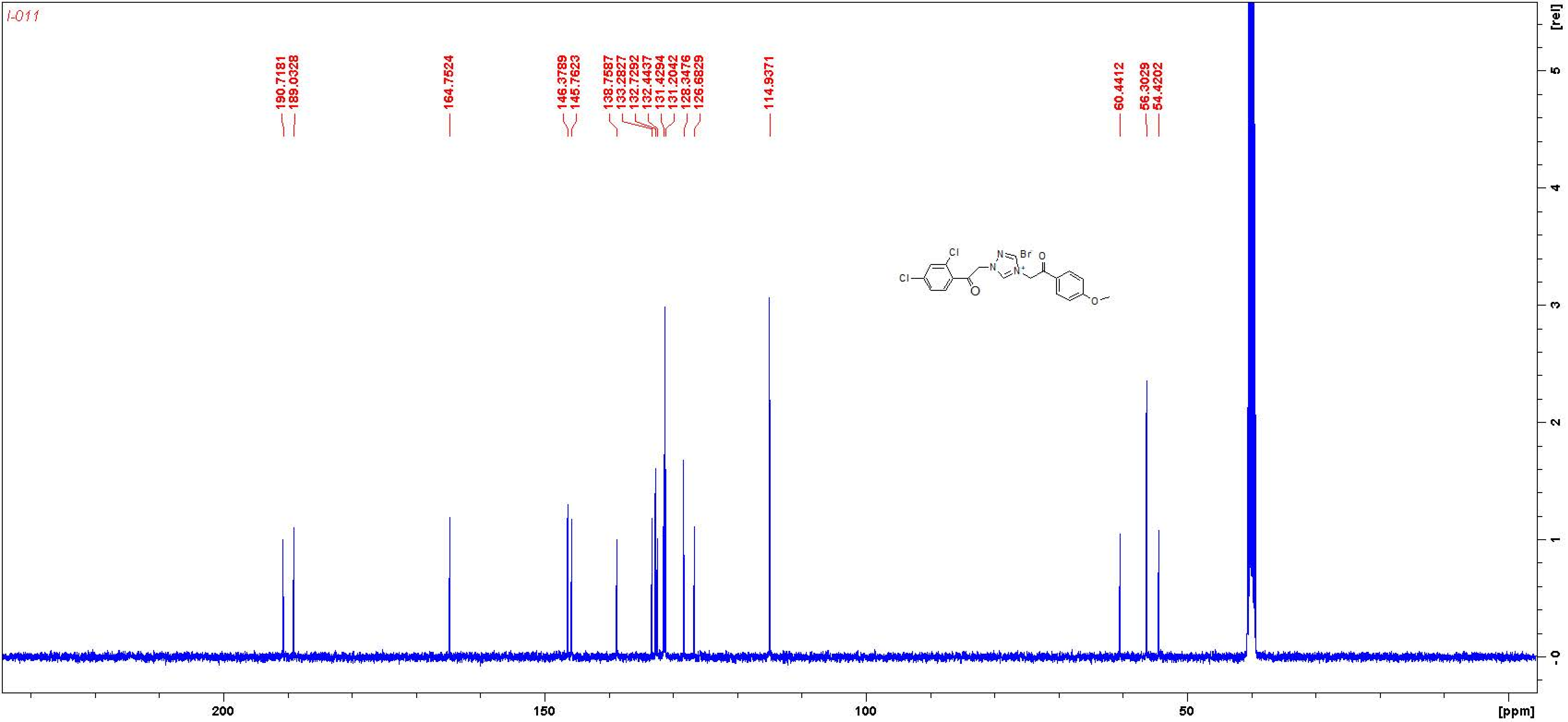
I-011
190.7181
189.0328
164.7524
146.3789
145.7623
138.7587
133.2827
132.7292
132.4437
131.4294
131.2042
128.3476
126.6829
114.9371
60.4412
56.3029
54.4202
Cl
Cl
N
Br
N
N+
O
O
O
[rel]
5
4
3
2
1
0
200
150
100
50
[ppm]

File   :D:\MSDChem\1\DATA\MZF-1-290322.D
Operator   : Veaceslav Kulcitki
Acquired   : 29 Mar 2022 12:50   using AcqMethod FENICOL_MASA450.M
Instrument :   GCMS Online
Sample Name: MZF-1
Misc Info :
Vial Number: 1

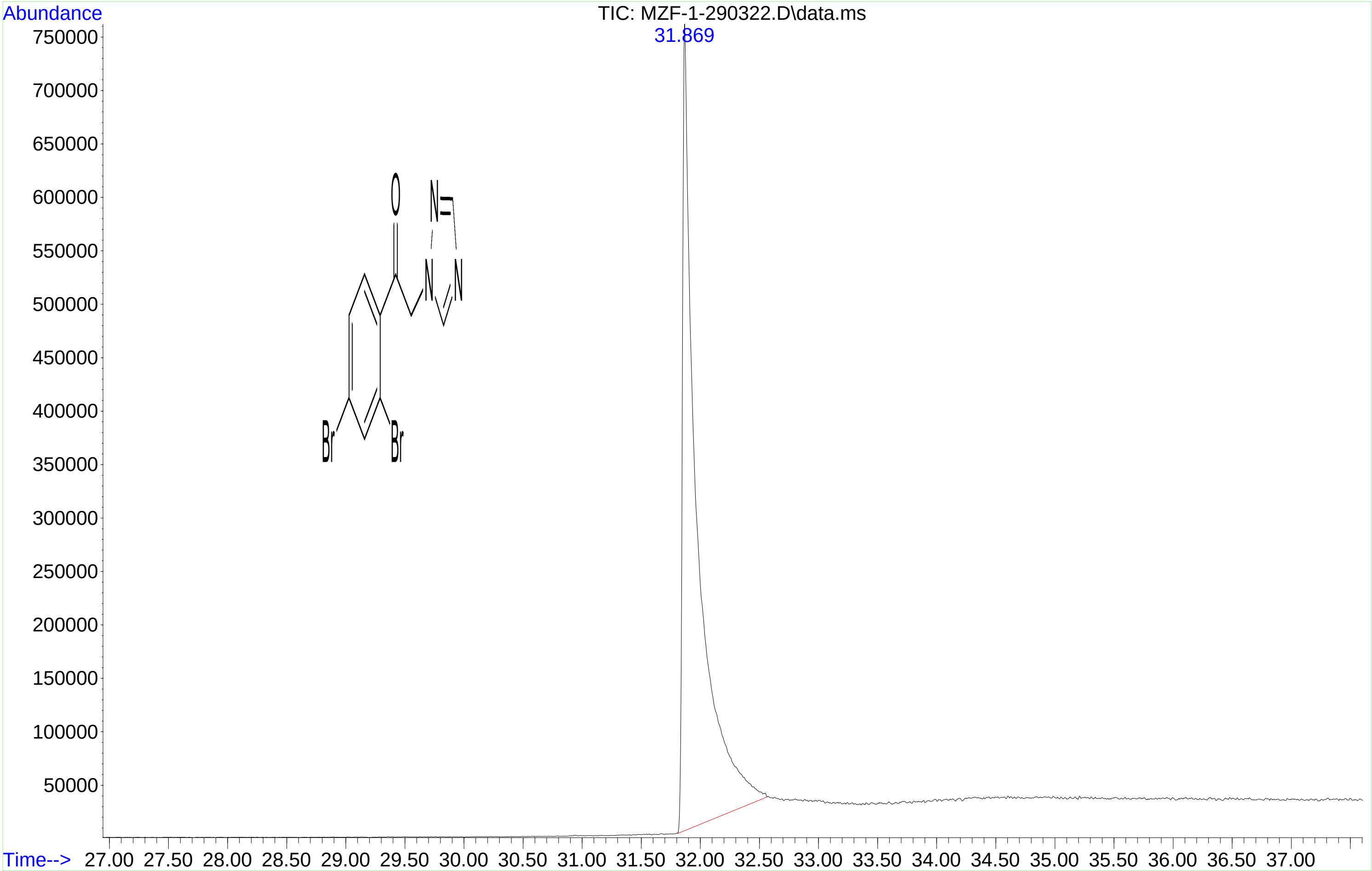

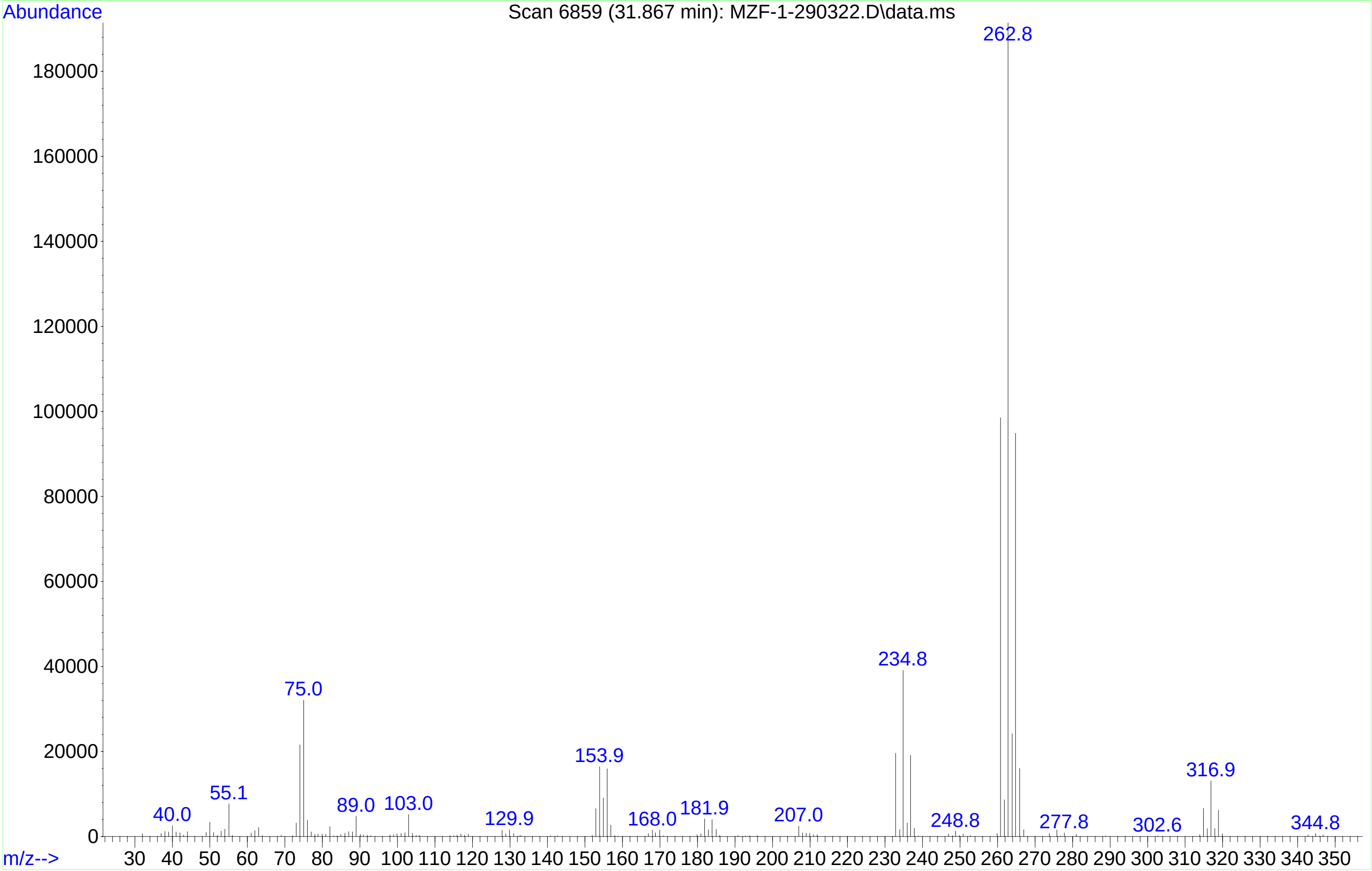

File   :D:\MSDChem\1\DATA\MZF-2-290322.D
Operator   : Veaceslav Kulcitki
Acquired   : 29 Mar 2022 13:34   using AcqMethod FENICOL_MASA450.M
Instrument :   GCMS Online
Sample Name: MZF-2
Misc Info   :
Vial Number: 2

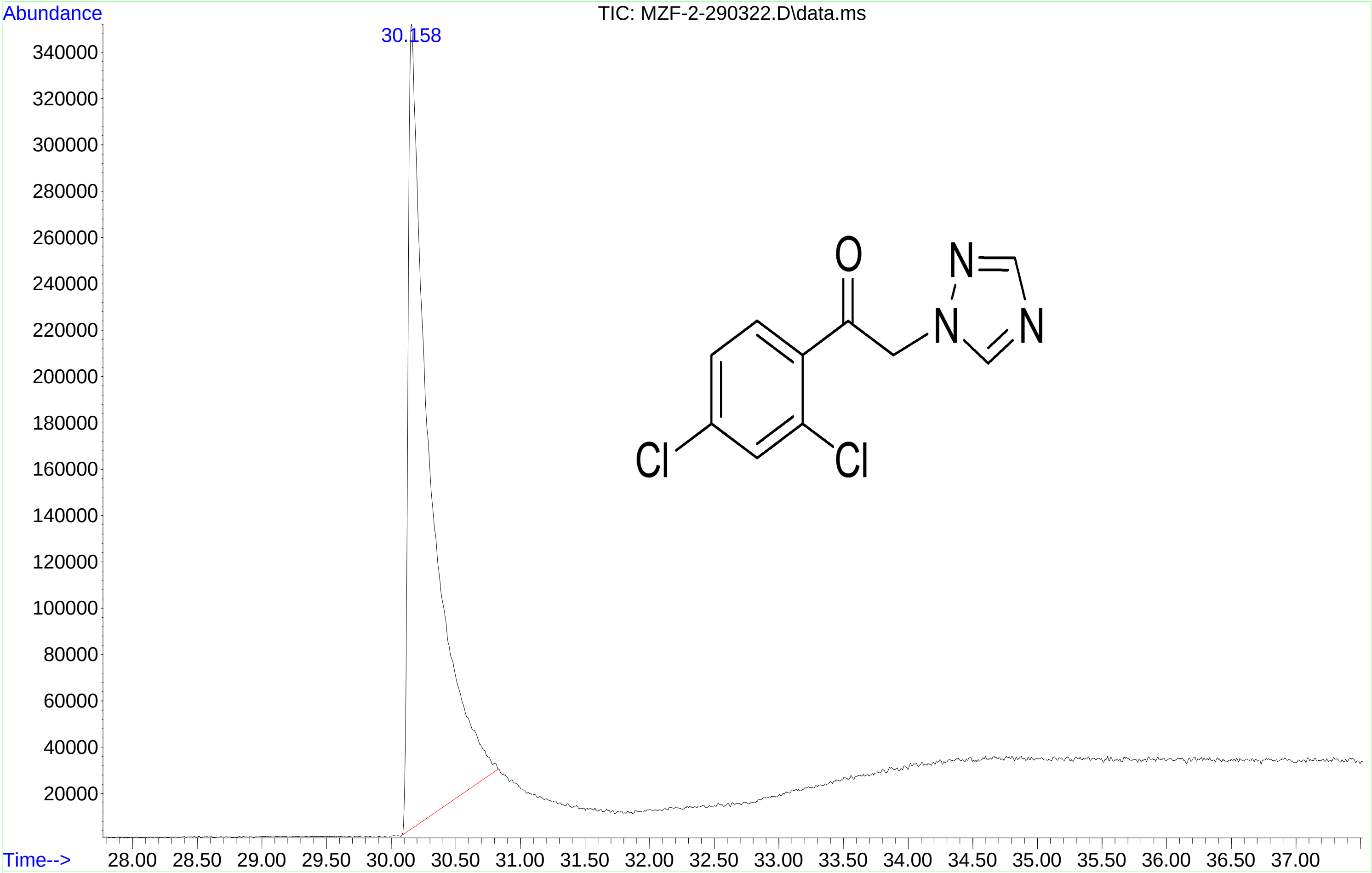

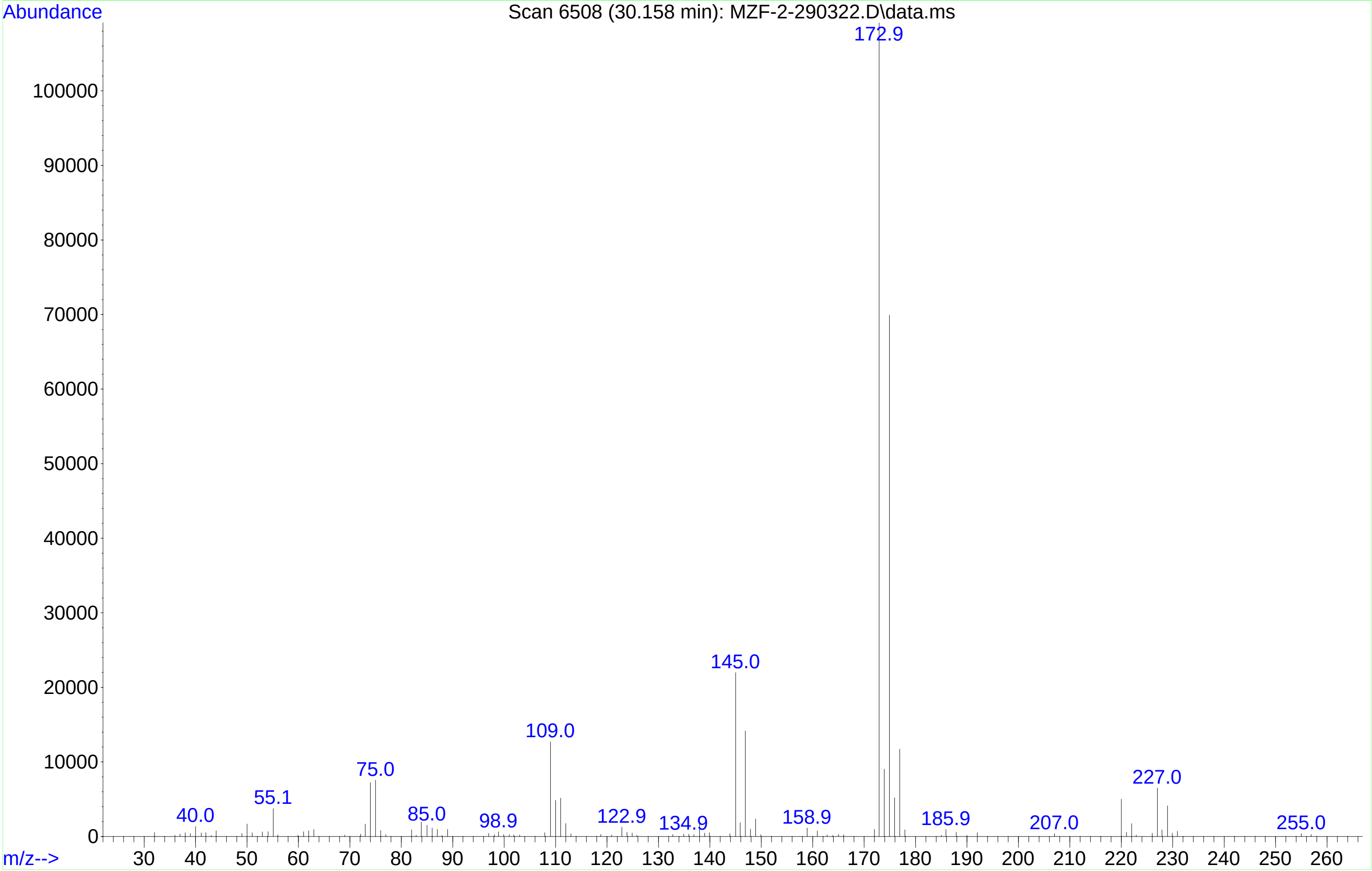

```
File        :D:\MSDChem\1\DATA\MZF-3-290322.D
Operator    : Veaceslav Kulcitki
Acquired    : 29 Mar 2022  14:19     using AcqMethod FENICOL_MASA450.M
Instrument  :    GCMS Online
Sample Name: MZF-3
Misc Info   :
Vial Number: 3
```

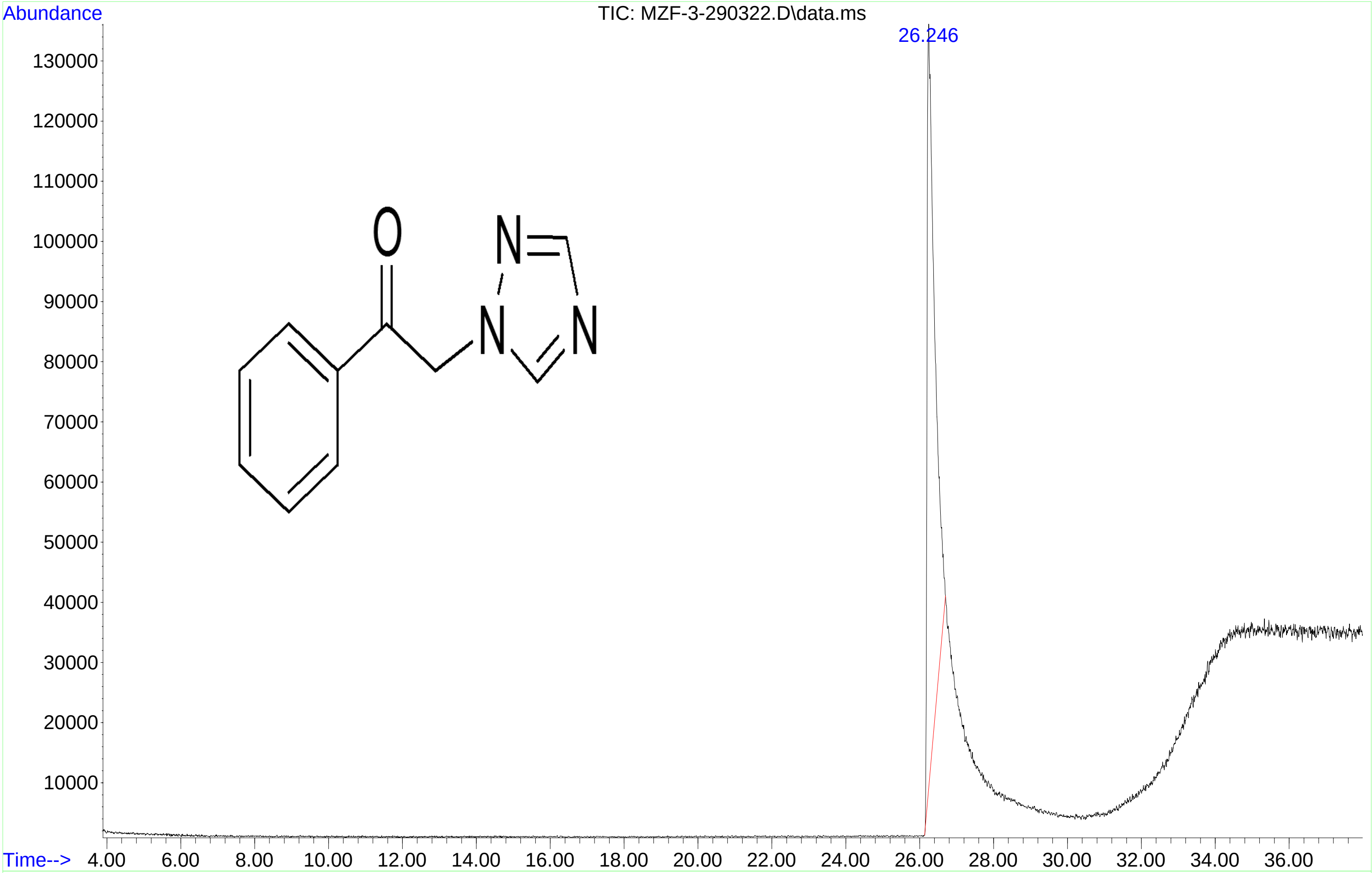

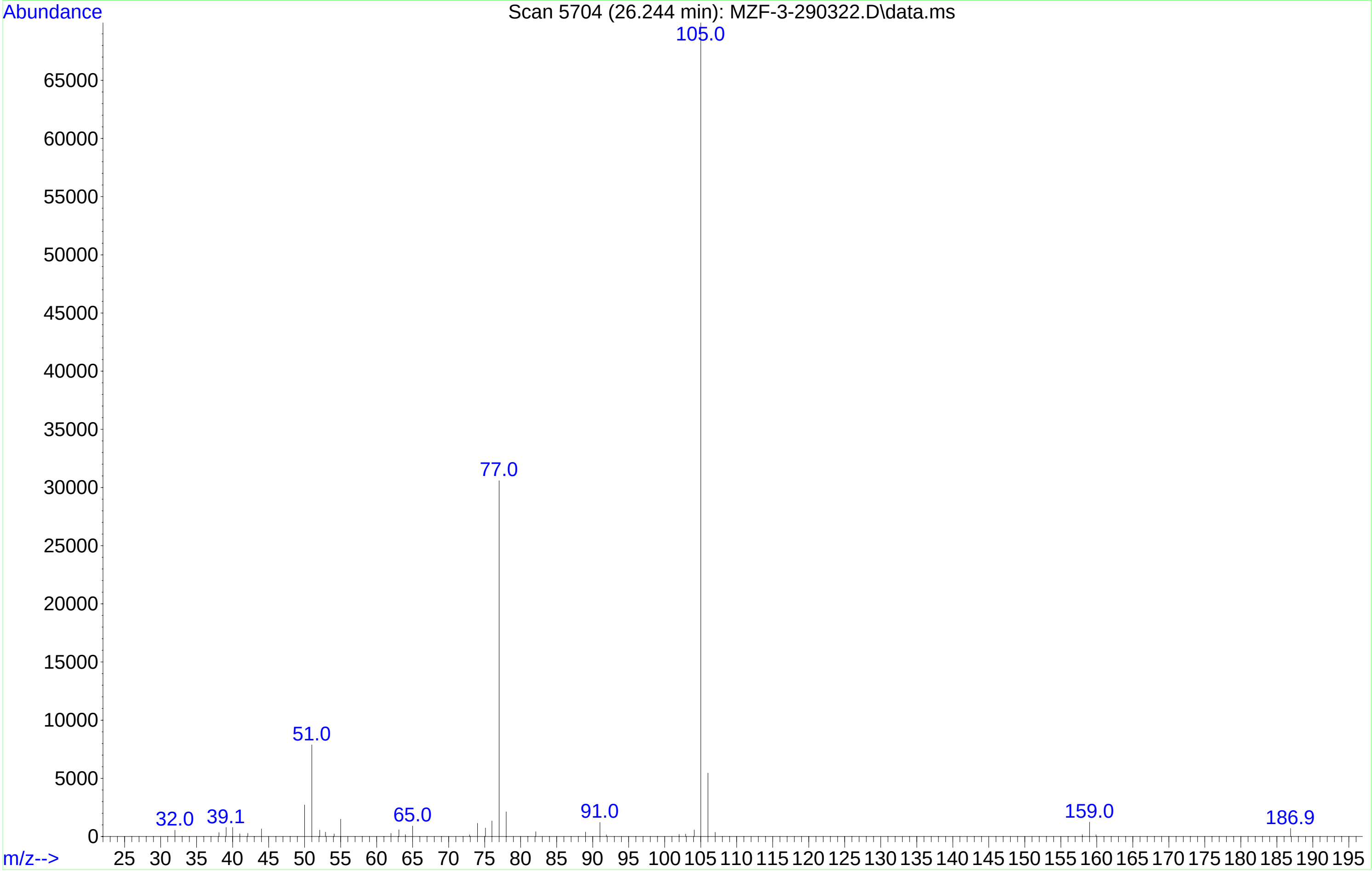

```
File        :D:\MSDChem\1\DATA\MZF-4-290322.D
Operator    : Veaceslav Kulcitki
Acquired    : 29 Mar 2022  15:03     using AcqMethod FENICOL_MASA450.M
Instrument :    GCMS Online
Sample Name: MZF-4
Misc Info   :
Vial Number: 4
```

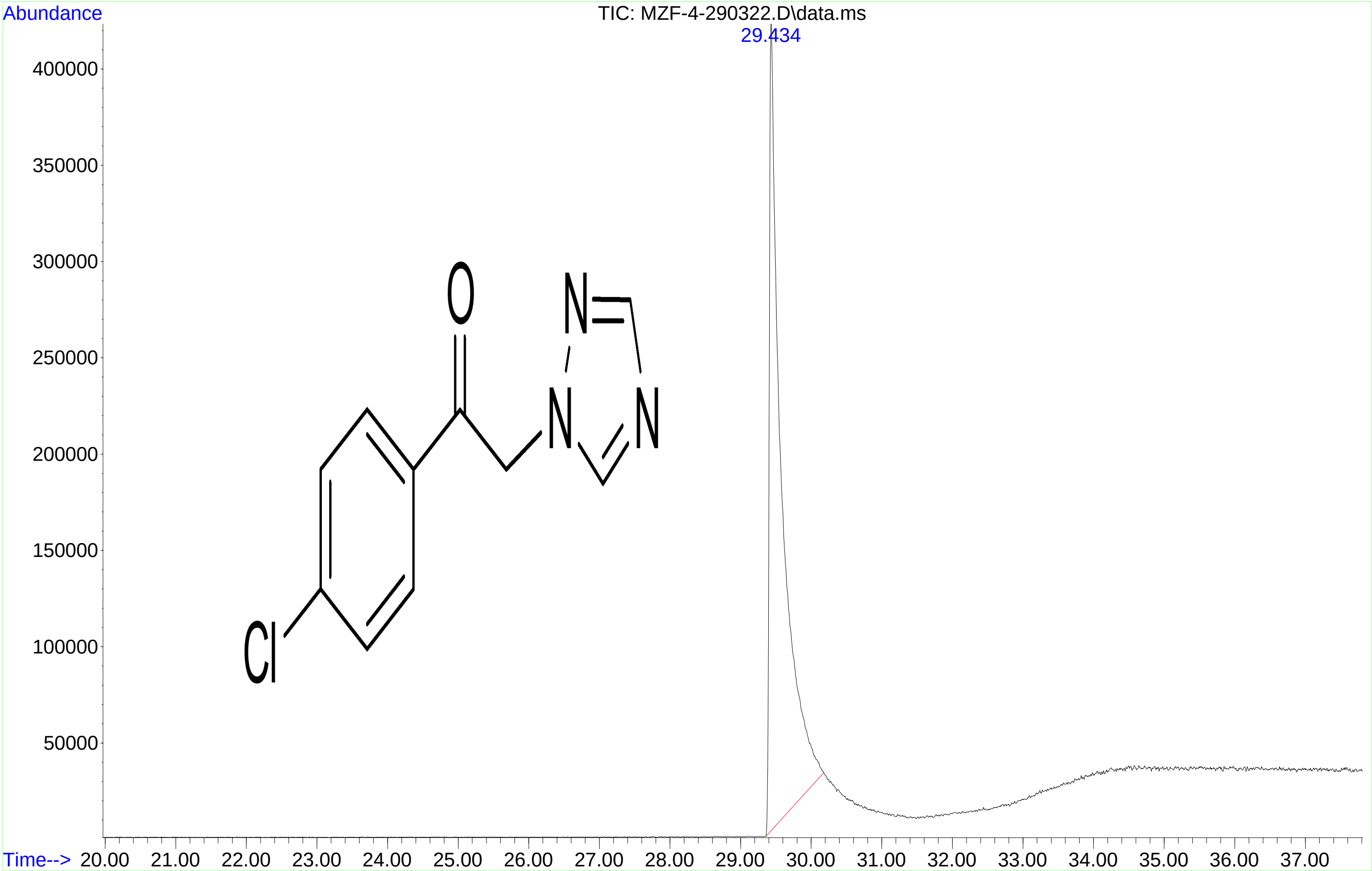

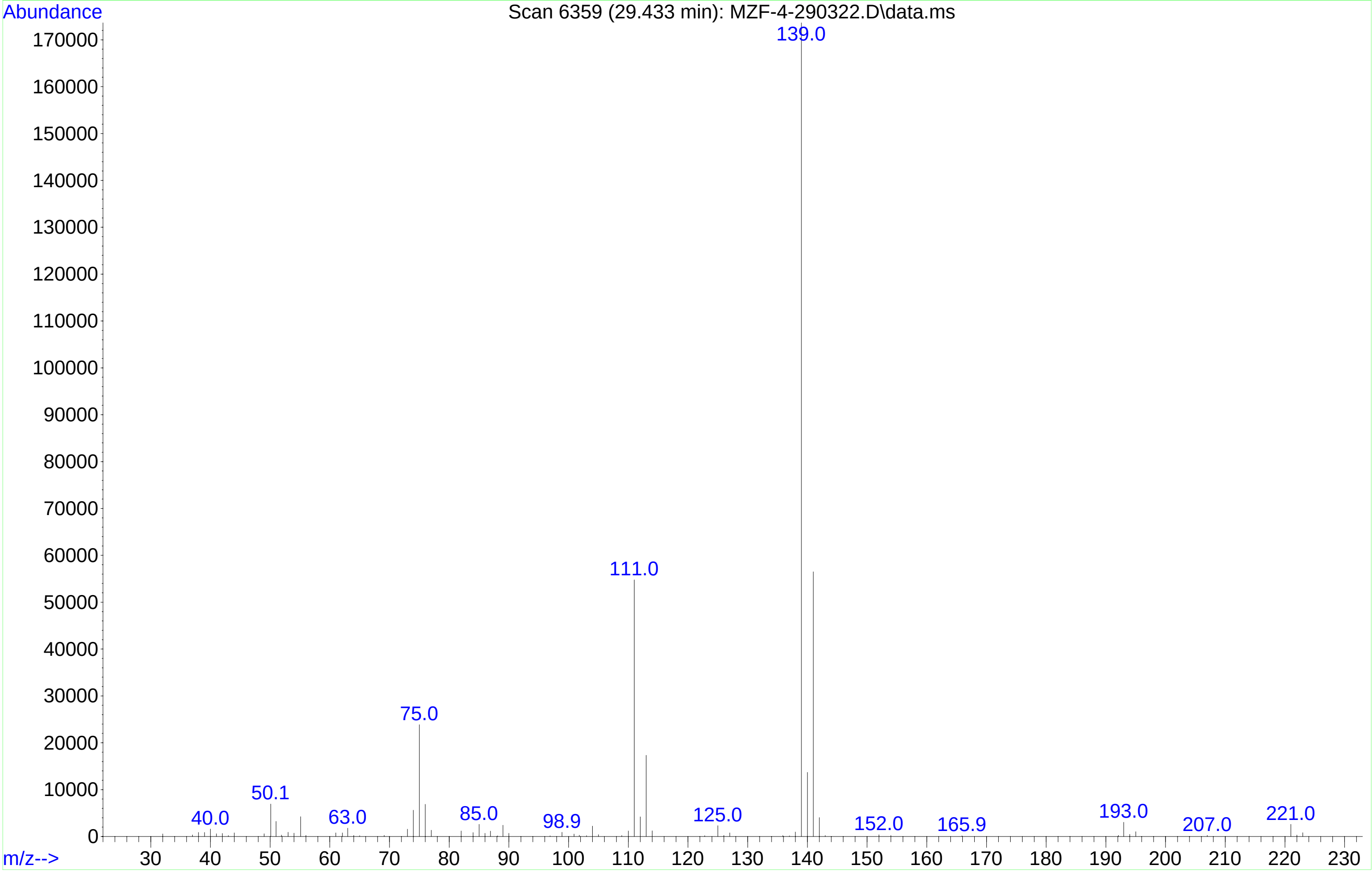

```
File        :D:\MSDChem\1\DATA\MZF-5-290322.D
Operator    : Veaceslav Kulcitki
Acquired    : 29 Mar 2022  15:47     using AcqMethod FENICOL_MASA450.M
Instrument  :    GCMS Online
Sample Name: MZF-5
Misc Info   :
Vial Number: 5
```

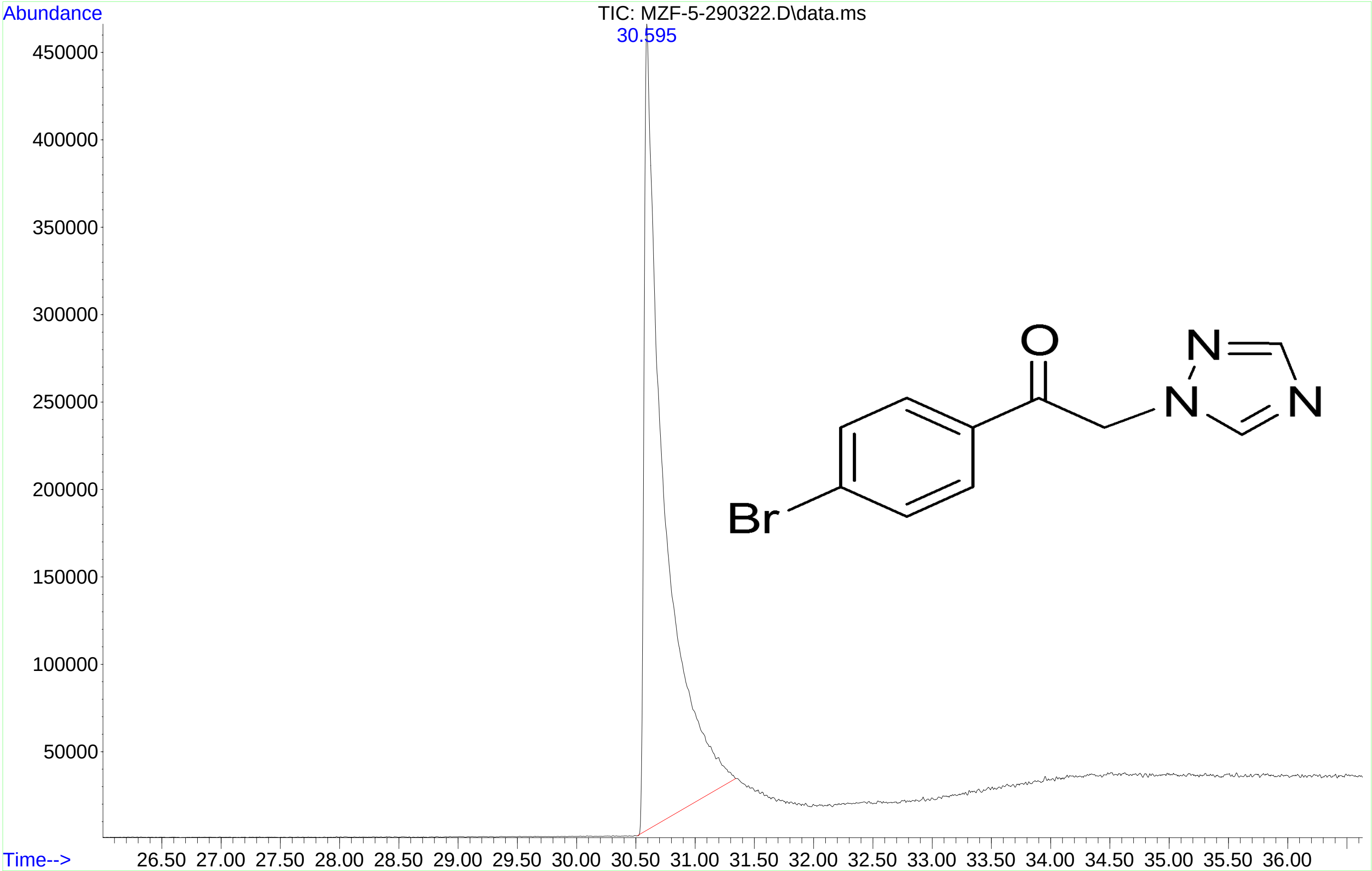

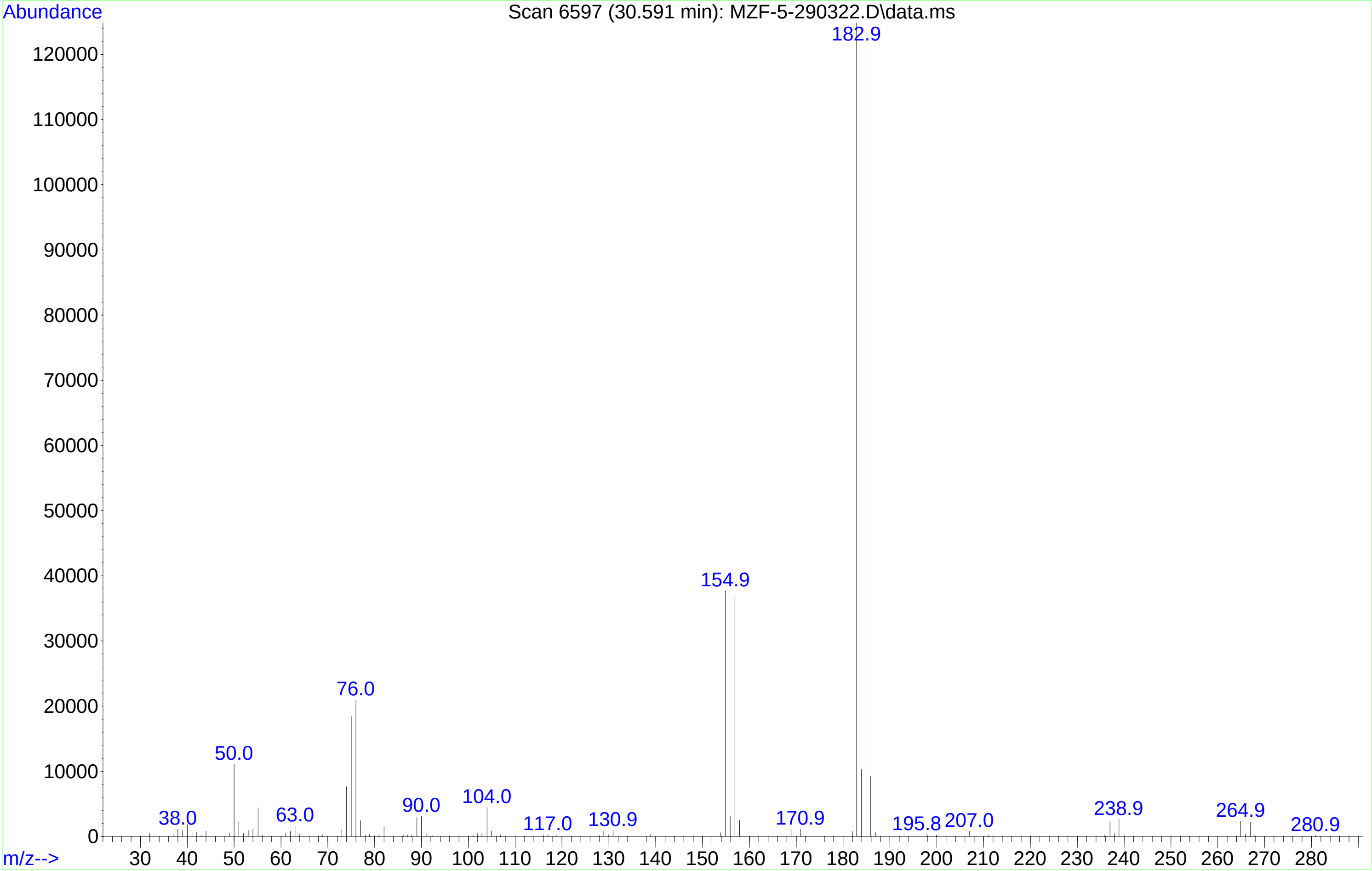

Supplement: Supplementary file 1 [file antibiotics-11-00588-s001.zip › antibiotics-1642229-supplementary.pdf]
